# Near-Infrared Light-Accelerated Bioorthogonal Drug Uncaging and Photothermal Ablation by Anisotropic Pd@Au Plasmonic Nanorods

**DOI:** 10.1021/jacs.5c07261

**Published:** 2025-06-26

**Authors:** M. Carmen Ortega-Liebana, Jana Travnickova, Catherine Adam, Davir González-Calderón, Álvaro Lorente-Macías, Charles Lochenie, Raul Arenal, E. Elizabeth Patton, Asier Unciti-Broceta

**Affiliations:** ‡ Edinburgh Cancer Research, Cancer Research UK Scotland Centre, Institute of Genetics and Cancer, 3124 University of Edinburgh, Crewe Road South, Edinburgh EH4 2XR, U.K.; ⊥ Department of Medicinal and Organic Chemistry and Unit of Excellence in Chemistry Applied to Biomedicine and Environment, Campus Cartuja s/n, 16741 University of Granada, Granada 18071, Spain; ≠ GENYO, Pfizer/University of Granada/Andalusian Regional Government, Avda. Ilustración 114, Granada 18016, Spain; † Instituto de Investigación Biosanitaria ibs.GRANADA, Granada 18071, Spain; # MRC Human Genetics Unit, Institute of Genetics and Cancer, 3124 University of Edinburgh, Edinburgh EH4 2XU, U.K.; ≤ Pandemic Science Hub, Institute for Regeneration and Repair, 3124 University of Edinburgh, Edinburgh EH16 4UU, U.K.; § Laboratorio de Microscopias Avanzadas (LMA), 16765 Universidad de Zaragoza, Zaragoza 50018, Spain; ¥ Instituto de Nanociencia y Materiales de Aragon (INMA) CSIC− University of Zaragoza, Zaragoza 50009, Spain; ± ARAID Foundation, Zaragoza 50018, Spain

## Abstract

Selective activation of chemotherapeutics at the tumor site via bioorthogonal catalysis is a promising strategy to reduce collateral damage to healthy tissues and organs. Despite significant advances in this field, targeted drug activation by transition-metal catalysts is still limited by insufficient spatiotemporal control over the metal-mediated uncaging process. Herein, we report the development of anisotropic Pd@Au plasmonic nanorods with the capacity to accelerate dealkylation reactions under near-infrared (NIR) irradiation, thereby enabling precise control over when and where these catalytic devices are switched on. We also show that the stability and *in cellulo* chemical properties of Pd@Au nanorods are enhanced by Au–S functionalization with PEGylated phospholipids and report the development of a novel masking group for prodyes and prodrugs: the POxOC group, designed to improve physicochemical properties and the rate of the Pd-triggered dye/drug release process. NIR-photoactivation of lipo-Pd@Au nanorods is able to catalyze the uncaging of inactive drug precursors and release heat to the environment, killing cancer cells in culture and xenografted in zebrafish. This work provides a novel targeted strategy for photothermal chemotherapy by NIR-laser focalization.

## Graphical abstract

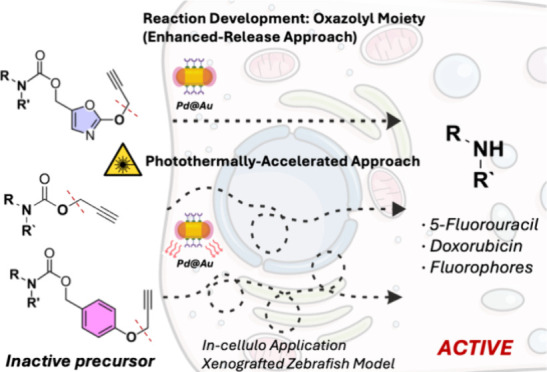

## Introduction

Standard chemotherapeutics have a number of well-known limitations, including systemic adverse effects due to off-tumor toxicities, reduced bioavailability, and short half-life. Drug delivery systems emerged from the need to improve such chemotherapy interventions, sparking the rise of a rich diversity of nanodevices and drug-loading strategies with promising potential to kill cancer cells.^1,2^ Nanocarriers can improve pharmacokinetics and site-specific pharmacodynamics, with notable examples already approved for clinical use.^3^ More recently, researchers have explored the use of nanotechnologies with enzyme-like capabilities, a.k.a. nanozymes, to amplify the impact and prolong the duration of the therapeutic treatment.^4,5^ To avoid reliance on biological mediators, so-called bioorthogonal nanozymes have expanded the nanozyme concept toward the use of abiotic transition-metal catalysts (TMCs) to convert inactive prodrugs into cytotoxic drugs, opening the possibility of “synthesizing” drugs at the site of the disease (e.g., in a tumor) with increased selectivity.^6–8^

The crowded biomolecular environments of the extracellular and intracellular milieu challenge the stability and chemical reactivity of TMCs. Such a fundamental problem has been tackled in various ways; for instance, by loading the catalyst as nanoparticles (NPs) or discrete organometallic complexes into polymeric implants,^9–15^ metal organic frameworks (MOFs) or Au-cored lipid NPs,^16,17^ exosomes or macrophages,^18,19^ mesoporous, biodegradable or single-chained NPs,^20,21^ black phosphorus nanosheets or even into proteins,^22,23^ to name some illustrative examples. However, achieving control over where, when, and how long the catalytic process takes place requires more sophisticated solutions. Rotello designed supramolecularly regulated and thermoresponsive strategies to control substrate access to TMCs,^24,25^ whereas Qu and co-workers proposed the incorporation of photoswitchable azobenzenes activated by UV light and DNA-gated acid-activated nanodevices to regulate substrate-catalyst interactions.^26,27^ Motivated by this challenge, we considered that stimuli-responsive nanozymes whose catalytic properties are remotely accelerated by noninvasive irradiative means could offer interesting advantages, especially with harmless tissue-penetrating NIR light.

Au nanorods are in the spotlight of photoinduced experimental therapies because of their remarkable plasmonic properties. This unique optical feature generates plasmon resonance along the surface of the nanomaterial when exposed to NIR irradiation, resulting in an increment of the nanostructure temperature after plasmon decay.^28^ Work by Lee et al. showed the potential of plasmonic Au nanospheroids to harness NIR light and release fluorescent dyes, although drug uncaging studies in cells were not reported in their study.^29^ Based on our own experience in bioorthogonal Au chemistry,^11,13,20^ we hypothesized that a disadvantage of their approach was to rely on Au metal for both plasmon effects and catalysis, since the chemical reactivity of loosely protected Au-NPs is rapidly deactivated by thiol-rich biomolecules, which are ubiquitous in biological media and cells. Therefore, enthused by the goal of controlling bioorthogonal catalysis on demand, we embarked on an investigation to develop nanozymes comprising a plasmonic Au nanorod core doped with a second transition metal to function as the catalytic unit under NIR-light-induced heating (Figure 1a). From the available catalog of abiotic TMCs, Pd is arguably the most widely applied in bioorthogonal catalysis due to its low toxicity in metallic form and chemical compatibility in biological media^30–37^ and, therefore, a rational partner for the construction of our bimetallic nanosystem. Moreover, Pd is an efficient electron acceptor and has been shown to enhance Au plasmonic features.^38–41^ To minimize nonspecific protein adsorption on the exposed Au surface and intracellular particle aggregation, we protected the Au metal with DSPE-PEG-SH, a thiol-modified PEGylated phospholipid (Figure 1b). Using this multicomponent design, herein we demonstrate the application of NIR light to accelerate depropagylation reactions in cells, including the uncaging of two potent chemotherapeutic drugs and the induction of photothermal effects. We also report the design and development of a novel Pd-labile masking group: POxOC (see Figure 1c). This group incorporates an oxazolyl moiety that accelerates the self-immolation of the masking group upon *O*-depropagylation and enhances the physicochemical properties of the masked prodye/prodrug. The therapeutic scope of these new tools was corroborated in a breast cancer and colorectal cancer xenograft model in zebrafish.

**Figure 1:**
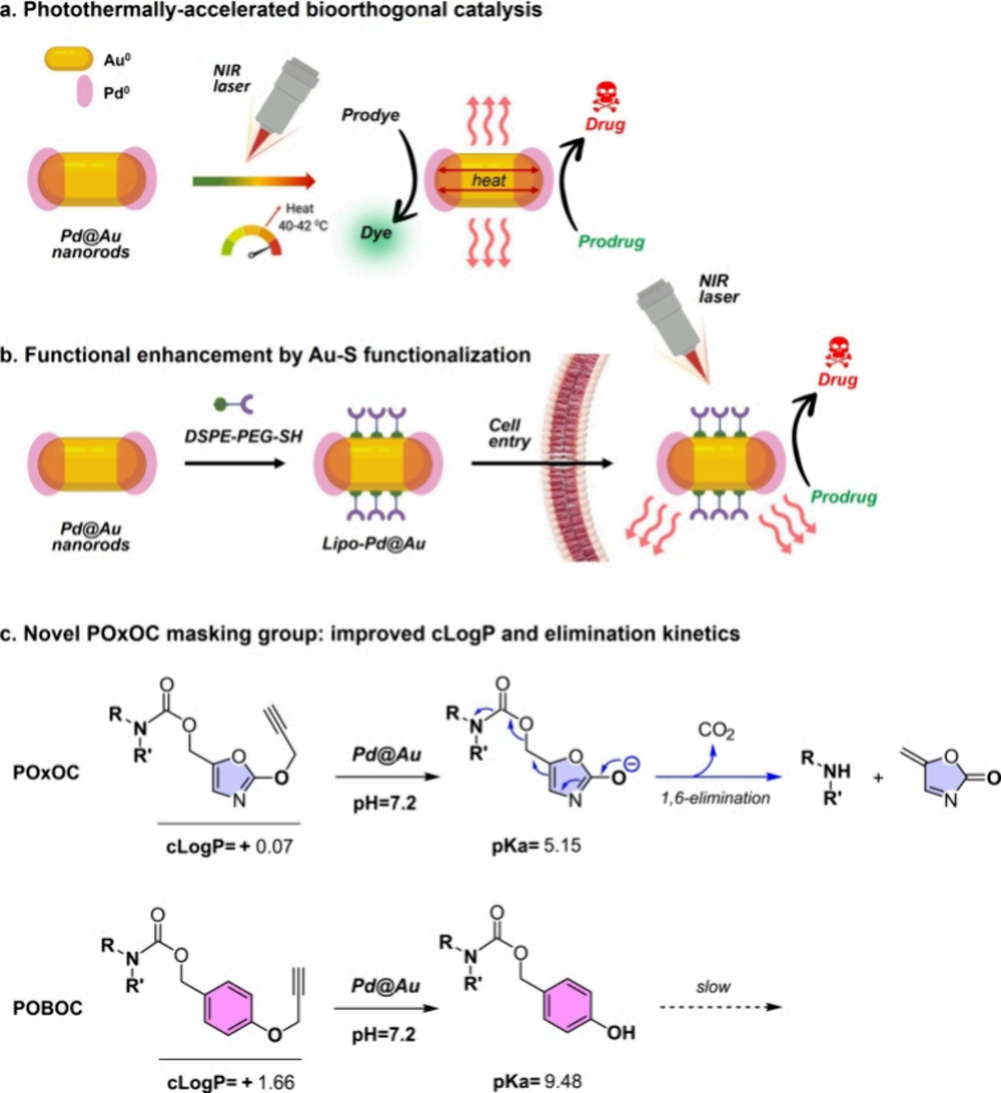
(a, b) NIR-stimulated bioorthogonal catalytic nanodevices. (**c)** Novel (2-propargyloxyoxazole-4-methyl)­oxycarbonyl, a.k.a. POxOC, masking group.

## Results and Discussion

### Synthesis and Functional Screening of Pd@Au Nanorods

Au nanorods were prepared via a seed-mediated sequential growth method^42^ before being doped with Pd. The distribution of Pd on the Au nanostructure can impact the generation and transfer of hot electrons from the Au core to Pd sites by surface plasmon decay, which will thereby affect catalytic performance in aqueous media. Therefore, to investigate the catalytic enhancement provided by the incorporation of Pd metal onto plasmonic Au nanorod, we synthesized three types of **Pd@Au** NPs using different Pd metal deposition methods, which generated Pd-tipped (**NP1**), Pd-shelled (**NP2**) and Pd-spotted (**NP3**) Au nanorods (see TEM images in Figure 2a and Figure S1; and experimental details in the Supporting Information­(SI)). The off–on fluorescent probe **Pro-Res**, which releases strongly fluorescent resorufin after Pd-mediated *O*-propargyl cleavage (Figure 2b),^18^ was used to test the chemical properties of the mono (only Au) and bimetallic nanorods. Reactions were performed at room temperature in physiological media (PBS with/without serum), and the effect of NIR irradiation was investigated using an 808 nm laser diode (model MDL-III-808–2W, Changchun New Industries Optoelectronics Technology Co.). As shown in Figure 2c, no catalytic activity was detected using monometallic Au nanorods, not even under NIR irradiation, which indicates that the nanorod morphology is inefficient for catalytic functions. Remarkably, **Pd@Au NP1** and **NP3**, featuring anisotropic growth of Pd on the Au nanorods, demonstrated high laser-enhanced catalysis upon NIR irradiation (Figure 2c), confirming the effective heat transfer within the bimetallic nanostructures. Interestingly, while **NP2**—which are fully covered by Pd all over the Au core—exhibited higher catalytic efficacy in the dark (no NIR) compared to **NP1** and **NP3** (Figure 2d), the latter ones displayed significantly higher catalytic properties under NIR irradiation, suggesting that anisotropic Pd coating is more favorable to transfer heat to the Pd metal and, thereby, enhance the catalytic reaction. **NP1** (named **Pd@Au** from now on), which features cap-shaped Pd deposited at the tips of the Au nanorod core, achieved >98% yield after 15 min reaction under NIR irradiation, being the best-performing nanostructure. In contrast, in the absence of NIR irradiation, the reaction yielded only 2.5% yield, requiring 24 h to reach an equivalent yield to that obtained under 15 min of NIR irradiation (SI, Figure S2). In agreement with the expected interaction between the naked Au surface and thiol-rich biomolecules, slightly lower yields were obtained in the presence of serum under all conditions, an unfavorable aspect for the use of **Pd@Au** in biological environments that motivated us to consider further investigations of NP shielding.

**Figure 2:**
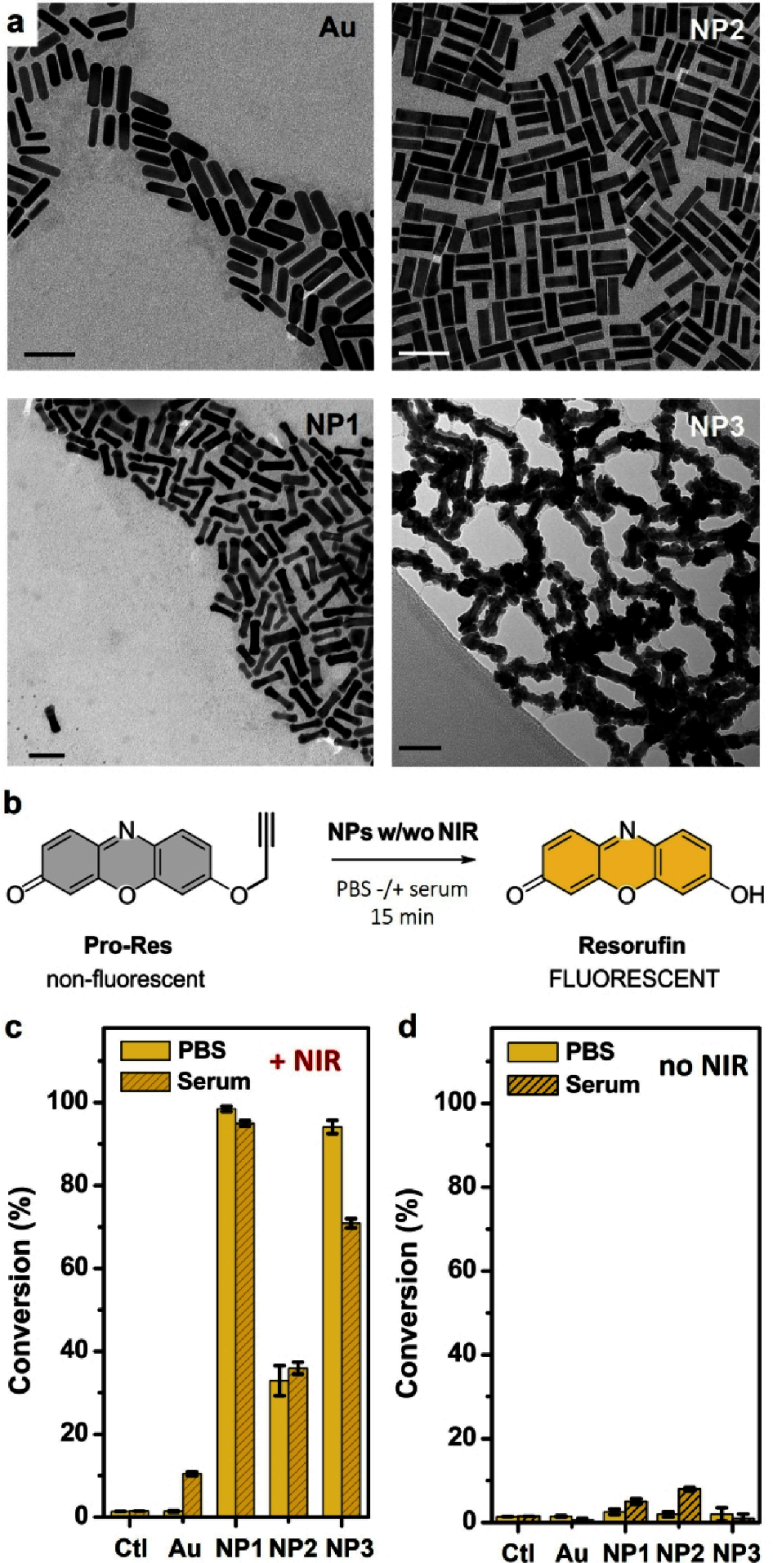
(a) TEM images of Au nanorods and bimetallic NP1, NP2 and NP3. Scale bar: 50 nm. (b), Fluorogenic assay: **Pro-Res** (100 μM) and **Au-NPs** (Au) or **NP1–3** (40 μg/mL) in PBS or PBS + 10% FBS (serum). (c) Comparative study of the conversion efficiencies (in %) after 15 min NIR irradiation. Error bars: ± SD (*n* = 3). (d) Conversion efficiencies (in %) after 15 min incubation in the dark. % calculated from fluorescence intensity at λ_ex/em_ = 550/580 nm using a standard curve of resorufin. Negative control (Ctl): nonfluorescent **Pro-Res** (100 μM) alone. Error bars: ± SD (*n* = 3).

**Pd@Au** nanostructures were fully characterized by high-angle annular dark-field (scanning) transmission electron microscopy (HAADF-STEM) for atomic-scale imaging and energy-dispersive X-ray spectroscopy (EDS) for detailed elemental analysis. As shown in Figure 3a, EDS elemental maps clearly show that Pd is mostly located at the tips of the Au nanorod, confirming a tip-coated nanostructure configuration (see also SI, Figure S3). Pd content was determined to be 28.3% (*w*/*w*) by inductively coupled plasma mass spectrometry (ICP-MS).

**Figure 3:**
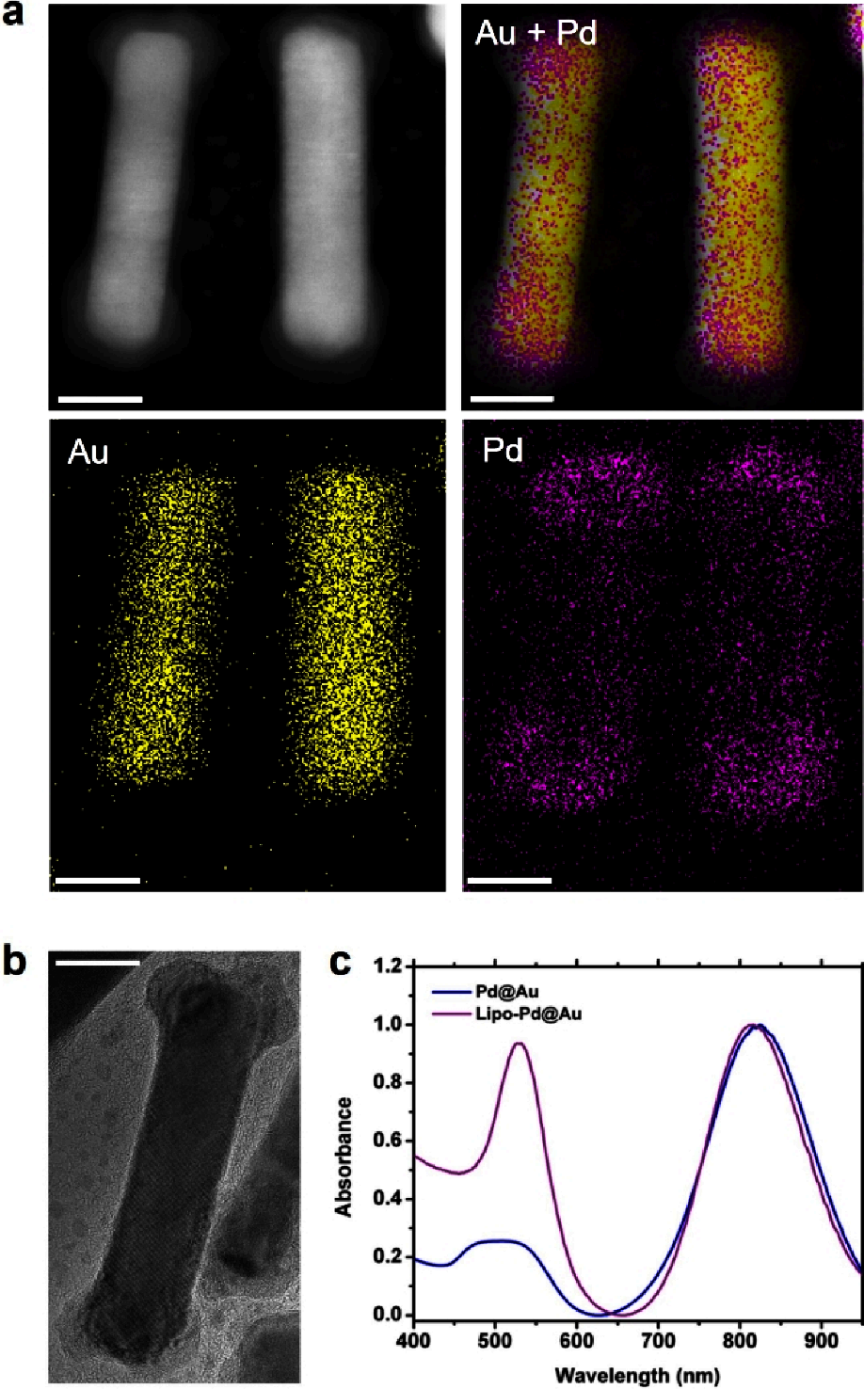
Characterization of **Pd@Au**. (a) HAADF-STEM image and corresponding EDS elemental maps. (b) HR-TEM image of a **Pd@Au**. (c) and UV–vis-NIR absorption spectra of **Pd@Au** and **lipo-Pd@Au**. Scale bars = 10 nm.

High-resolution TEM (HR-TEM) images clearly show the epitaxial growth of Pd on Au nanorods (Figure 3b).^42^ In comparison, Pd-shelled Au nanorods (**NP2**) display a layer of a Pd shell homogeneously deposited around the surface of the Au nanorod (SI, Figure S4). The absorbance spectrum of **Pd@Au** shows a maximum peak centered at 820 nm (Figure 3c), slightly red-shifted compared to the original Au nanorods (SI, Figure S5).

### Design and Synthesis of Novel Pd-Labile Masking Group for Amino-Functionalized Dyes and Drugs

The off–on sensor **Pro-Res** serves as an optimal surrogate model to probe the capacity of novel catalysts to activate *O*-alkylated prodrugs such as 2,4-dipropargyloxy-5-fluoropyridimidine (a.k.a. **diPro-5FU**), a Pd-labile precursor of 5FU.^37^ Yet, many clinically relevant drugs, such as doxorubicin, are modified as carbamate-based prodrugs by masking amino groups that are essential for target binding and anticancer activity.^6^ The propargyloxycarbonyl (POC)-protected NBD prodye **7a** was designed to model the activation of such prodrugs.^13^ However, we and others have shown^12,32,43^ that masking groups larger than POC can further decrease the pharmacological activity of the resulting prodrug, thus enlarging the bioactivity window between the prodrug and the parent drug. An illustrative example is the masking of the primary amino group of the daunosamine moiety of doxorubicin with the larger 4-(propargyloxy)­benzyloxycarbonyl (4-PBC) group, which leads to superior reduction of anticancer activity than the POC group.^12^ The downside of using 4-PBC is its high lipophilicity, which substantially increases the cLogP of the resulting prodrug compared to the POC group (+1.66 versus +0.06, respectively; see Figure 4a), leading to water solubility issues. Another disadvantage of the 4-PBC group is that upon *O*-propargyl cleavage, it has to undergo spontaneous 1,6-elimination to release quinone methide, CO_2_, and the active drug. The rate of this process is dependent on the p*K*_a_ of the phenolic OH, which has a value of over 9, and thus, it is mostly protonated at physiological pH, thereby slowing down drug release. This contrasts with the decarboxylation of carbamic acid, the depropargylated intermediate of POC, which has a p*K*_a_ value of 4.3. For prodrug strategies where small alkyl-carbamates are insufficient to reduce the bioactivity of the drug, it would be advantageous to make use of a masking group with a size close to that of the 4-PBC group, but that minimally increments the lipophilicity of the resulting derivative and rapidly self-immolates after the stimuli-triggered reaction.

**Figure 4:**
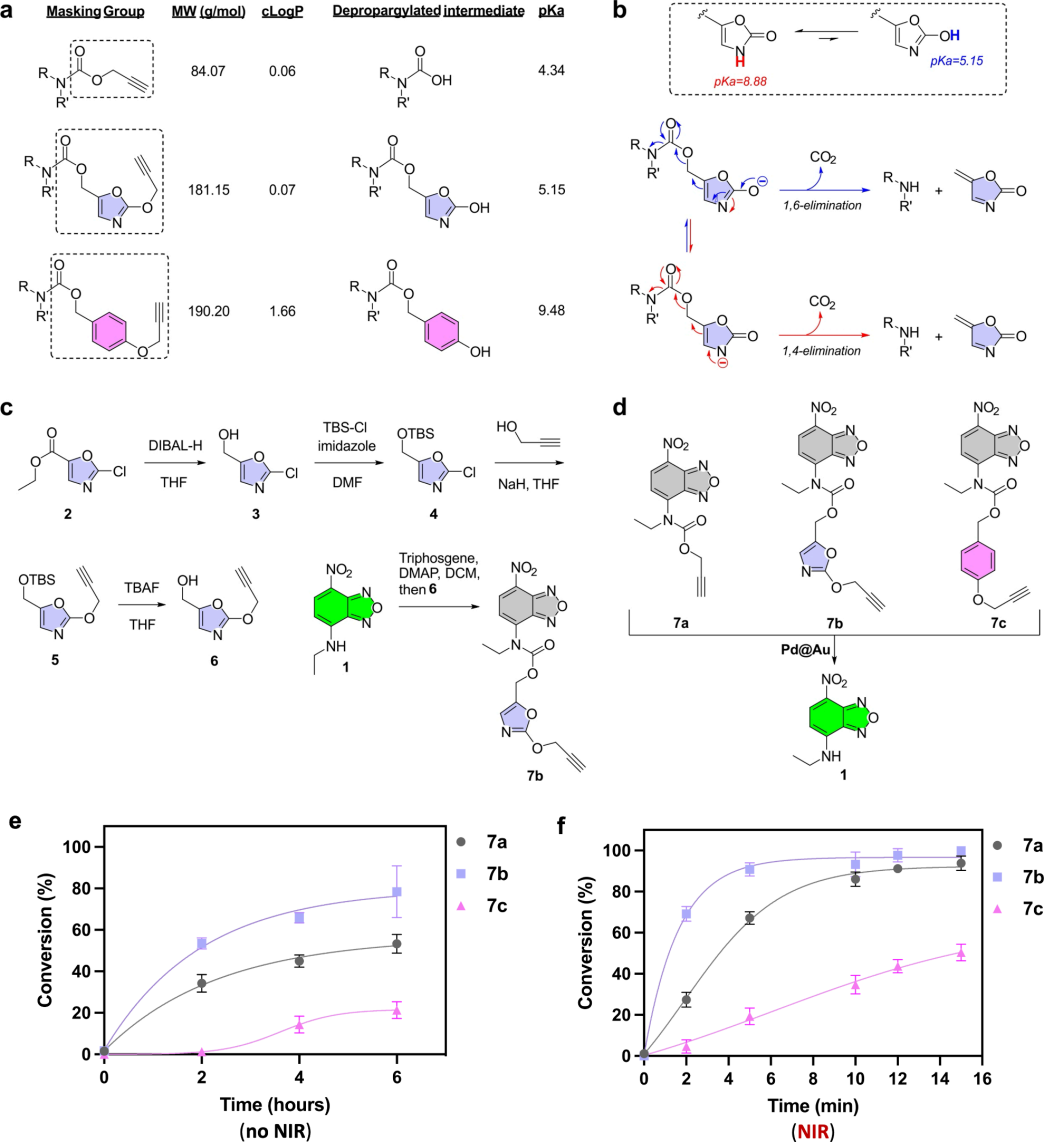
(a) Comparison of the contributions of different masking groups to MW and cLogP, and the estimated p*K*_a_ (calculated with MarvinSketch) of the depropargylated intermediates. (b) Equilibrium between the 2-oxazolone and 2-hydroxyoxazole tautomeric forms and proposed elimination routes. (c) Five-step synthesis of novel masked off-on Pd-activatable sensor **7b** from ethyl 2-chlorooxazole-4-carboxylate. (d) Conversion of off-on probes **7a**, **7b**, and **7c** into fluorescent dye **1** by **Pd@Au** NPs. (e, f) Kinetic study of the conversion of prodyes **7a–c** into fluorescent NBD without (e) and with (f) NIR irradiation.

With this aim in mind, we conceived the incorporation of an electron-deficient oxazole core as part of the self-immolative linker (Figure 4a,b). While 2-oxazolone is the dominant tautomer of the lactam-lactim pair,^44^ 2-hydroxyoxazole has an estimated p*K*_a_ of 5.15 and is susceptible to being trapped as an ether. Upon depropargylation of the newly designed (2-propargyloxyoxazole-4-methyl)­oxycarbonyl (POxOC) group, the low p*K*_a_ of the OH group, along with the irreversible formation of a thermodynamically stable product such as CO_2_, would favor the 1,6-elimination route (blue mechanism, Figure 4b), accelerating the self-immolative process relative to the 4-PBC group. Notably, the predicted contribution of the POxOC group to cLogP is only +0.07, a significant advantage to minimize the increase of the lipophilicity of the resulting probe/prodrug.

Encouraged by the favorable functional properties of the POxOC masking group, we synthesized the novel off-on probe **7b** following the procedure described in Figure 4c. In brief, ethyl 2-chlorooxazole-4-carboxylate, **2**, was reduced by DIBAL-H in THF to alcohol derivative **3**, which was then protected with TBS-Cl. Treatment with *in situ*-generated propargyl alkoxide gave rise to 2-propargyl oxy-4-(*tert*-butyldimethylsilyloxymethyl)­oxazole, **5**, which was deprotected with TBAF to produce alcohol **6**. Treatment of NBD-based dye **1** with triphosgene and DMAP in DCM, followed by the addition of **6**, gave rise to **7b** in moderate yield. 4-PBC-protected prodye **7c** (Figure 4d) was also synthesized (see full protocol in the SI) to test the capacity of **Pd@Au** to activate different probes.

To analyze and rank the uncaging rates of the different masking groups, the reactions were performed with and without NIR irradiation by treating the off-on masked prodyes **7a**–**c** at 37 °C in PBS + 10% serum with **Pd@Au** (Figure 4d). In the dark, fluorescence was measured at 2, 4, and 6 h in a microplate reader, and conversion rates are plotted in Figure 4e. As shown in the Figure, the activation of the novel POxOC-protected **7b** was the fastest among the three prodyes. Notably, the same trend was observed under NIR stimulation (Figure 4f), with >91% conversion of **7b** into fluorescent dye **1** after just 5 min of irradiation, compared with 67% and 19% for **7a** and **7c**, respectively. To confirm the chemoselectivity of the nanocatalysts, Cbz-functionalized prodye **7d** (see SI) was synthesized as a control noncleavable by Pd chemistry. As expected, **Pd@Au** was unable to convert **7d** into **NBD** regardless of the use or not of NIR irradiation (SI, Figure S6).

### Design and Preparation of Lipo-Pd@Au

As mentioned before, Au has a high affinity for thiols. Consequently, the Au exposed surface of **Pd@Au** is liable to bind to biogenic thiols present in the serum, including glutathione and large proteins, which could be detrimental for the stability in circulation and performance of the nanocatalyst in and outside of cells. To address this issue, the dumbbell-shaped nanostructures were engineered with a thiol-functionalized PEG lipid, DSPE-PEG-SH. DSPE-PEG is a key component of PEGylated NPs currently approved by the FDA^45^ and has been shown to confer high stability, allowing the entry and release of NPs into tumors.^46,47^ Importantly, we reasoned that this Au-shielding strategy should not affect catalytic performance as the Pd tips would remain exposed to the environment to facilitate interaction with the substrate. **Lipo-Pd@Au** was successfully synthesized by Au–S functionalization and then characterized by microscopy and analytical instrumentation techniques. Their optical absorption spectra showed two typical plasmon peaks at 529 and 816 nm (Figure 3c). Upon surface modifications with DSPE-PEG-SH, no tailing or broadening of the plasmon peaks were observed, indicating excellent stability of the plasmonic colloids, even in the presence of oxidants (SI, Figure S7). Zeta potential reduced from +42 to +6.3 mV for **Pd@Au** and **lipo-Pd@Au**, respectively, confirming the successful displacement of cetyltrimethylammonium ions by DSPE-PEG-SH. The average hydrodynamic diameter measured by dynamic light scattering (DLS) changed from ∼52 nm for **Pd@Au** to ∼56 nm upon functionalization with DSPE-PEG-SH (SI, Figure S8). The shift in the hydrodynamic diameter provides further evidence of the formation of a phospholipid-PEG layer at the Au surface of **Pd@Au**. The nanocatalyst morphology and size were also studied by TEM after functionalization, demonstrating well-dispersed rod-shaped NPs (SI, Figure S9). Consistent with the requirements for hyperthermia treatment (i.e., local temperature increment in the range of 41–48 °C),^48^ **lipo-Pd@Au** displayed effective photothermal (PT) heating under NIR exposure (SI, Figure S10). Notably, a similar PT effect was observed in different media and pH, indicating that the nanodevices could operate in different tumor microenvironments. These results support the use of **lipo-Pd@Au** to enable catalytic activity modulation by NIR irradiation.

### Prodye Uncaging Study with Lipo-Pd@Au

Next, the uncaging capabilities of naked **Pd@Au** and **lipo-Pd@Au** were contrasted in the presence and absence of serum and NIR irradiation (808 nm, 1.0 W cm^–2^, 15 min). The fluorogenic assay was carried out with prodyes **7a** and **7b**, which, after Pd-mediated cleavage, release the green light-emitting fluorophore NBD, **1** (Figure 4d). To our delight, the catalytic properties of **lipo-Pd@Au** were unaffected by the presence of serum (SI, Figure S11a **-b**), which indicates that the phospholipid-PEG layer protects the NPs from direct interaction with serum proteins. Remarkably, after 8 reactions using recycled NPs, the catalytic properties of **lipo-Pd@Au** remain unaltered in the presence of serum after multiple rounds of laser irradiation (Figure S11c), whereas **Pd@Au** loses catalytic capacity. The reusability of this lipid-protected nanocatalyst demonstrates its high stability and catalytic activity in the presence of complex biomolecules, which is essential to overcome the operability limitations of most nanobased heterogeneous catalysts. The tolerability of human cells to treatment with **lipo-Pd@Au** was then tested in cancer cell lines (colorectal HCT116 and breast cancer MDA-MB-231 cells) and a noncancerous cell line (mammary epithelial MCF-10A cells). Viability assays showed no signs of toxicity in the dark at any of the concentrations tested (SI, Figure S12).

### Prodye Uncaging Study *In Viv* *o*

To assess the *in vivo* potential of the nanocatalyst, we performed a fluorogenic assay in a xenograft model in zebrafish. HCT116 cells were preincubated with **lipo-Pd@Au** for 12 h, to enable NP internalization, and Hoechst 33342, to fluorescently label cell nuclei. Then, cells were implanted in the perivitelline space (PVS) of 2 day old zebrafish casper embryos and the fish incubated with NBD-based prodye **7b** followed by NIR-laser irradiation for 30 s (Figure 5a). Experiments without NPs or without NIR irradiation were used as negative controls (see SI, Figure S13 for the non-NP control). Embryos were anesthetized with tricaine and embedded in 1% (w/v) low-melting-point agarose (in zebrafish water) for confocal imaging analysis. As shown in Figure 5b, zebrafish embryos containing **lipo-Pd@Au**-treated HCT116 xenografts and treated with prodye **7b** followed by NIR irradiation showed an intense green fluorescent signal at the site of cell transplantation. This confirms the local release of dye **1** and the functional compatibility of **lipo-Pd@Au** for *in vivo* studies. Of note, in agreement with the environmental sensitivity of **NBD**, which increases fluorescence intensity in hydrophobic media, green fluorescence emission colocalized with the cancer cells and locally diffused to the nearby area of the lipophilic yolk sac.^49^ The capabilities of the NPs were further confirmed with the use of prodye **7a** in 2-dpf (*fli1:GFP*)^50^ zebrafish larvae (SI, Figure S14).

**Figure 5:**
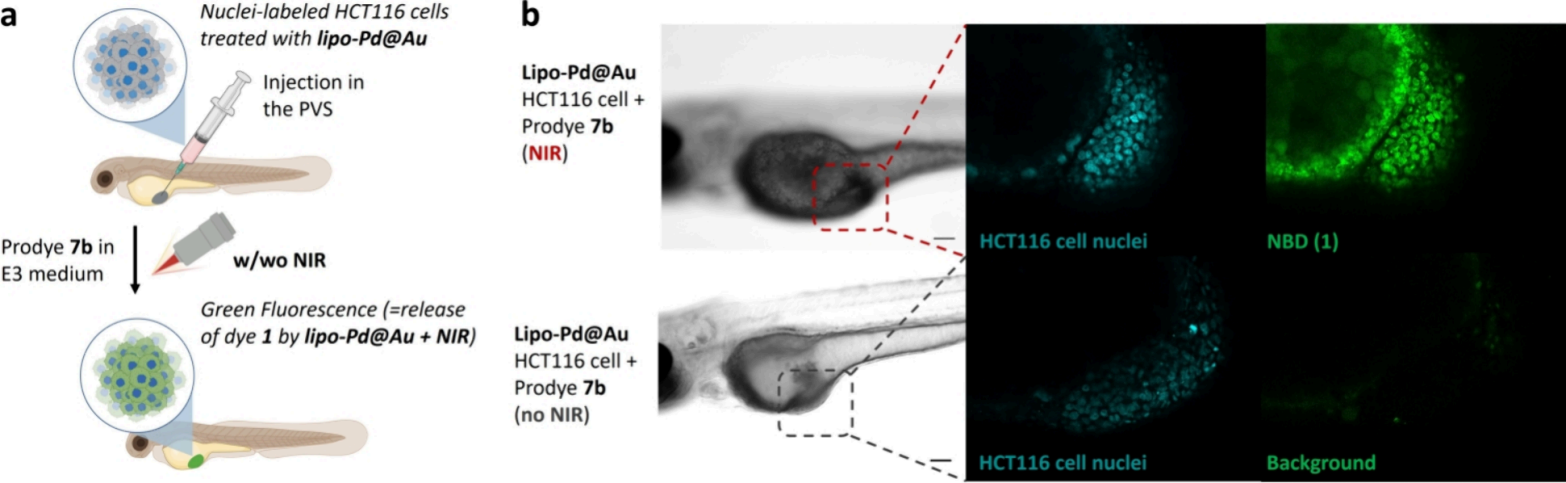
(a) NIR-triggered plasmonic **lipo-Pd@Au**-mediated prodye decaging in zebrafish xenografts. HCT116 cells were preincubated with **lipo-Pd@Au**, fluorescently labeled with Hoechst 33342 (in cyan), and injected into the perivitelline space (PVS) in 2-dpf zebrafish larvae. Zebrafish xenografts were randomly distributed into treatment groups, treated with **7b** in E3 medium, and analyzed after NIR irradiation by confocal microscopy. (b) Confocal analysis of the release of green fluorescent dye **1** from precursor **7b** in the HTC116 xenograft of zebrafish larvae loaded with **lipo-Pd@Au** and Hoechst with NIR irradiation for 30 s (top) or without NIR irradiation (bottom). *n* = 4. Scale bars = 100 μm.

### Drug Uncaging Studies *In Vitro* in Cell Culture and *In Vivo*

Encouraged by the fluorogenic studies, we tested the capacity of the plasmonic NPs to activate caged drugs in the cell culture under NIR irradiation. As a prerequisite, the cell uptake of **lipo-Pd@Au** (80 μg/mL) was quantified by measuring Pd/Au cell internalization by ICP-MS after 6 h incubation with a range of cell lines. Analysis showed **lipo-Pd@Au** were effectively endocytosed by three different cancer cell lines (HCT116, MDA-MB-231, and MCF-10A cells; SI, Figure S15). ICP analysis of HCT116 cells treated with **lipo-Pd@Au** verified the presence of NPs inside the vesicles in the cytoplasm. Interestingly, low levels of intracellular Pd/Au penetration were observed in the noncancerous MCF-10A cell line, an advantageous feature of the novel nanodevices. Then, we investigated the photothermal cell killing induced in cancer cells pretreated with **lipo-Pd@Au** (80 μg/mL) and irradiated with a NIR laser at two power densities (1.0 and 1.5 W/cm^2^) for 2, 5, and 10 min (SI, Figure S16). High reduction of cell viability (87%) was mediated by continuous irradiation after 10 min of irradiation at both power densities. Therefore, we selected the NIR power density of 1.0 W/cm^2^ for 5 min, which just led to approximately 18% reduction of cell viability, as the optimal irradiation protocol to facilitate the observation of the cytotoxic effect of drug uncaging.

Next, we performed combined photothermal chemotherapy experiments in cancer cell culture. As before, **lipo-Pd@Au** (80 μg/mL) were incubated with colorectal HCT116 cells for 6 h, and the excess of noninternalized nanodevices was washed off. The dipropargylated prodrug **8**,^37^ which mimics the chemical masking of **Pro-Res** and releases the clinically approved drug 5-fluorouracil (**5FU**) after double *O*-propargyl cleavage (Figure 6a), was used as the Pd-activatable inactive drug precursor. Intracellular prodrug activation by the nanodevices was tested with or without NIR irradiation (5 min). Cell viability was measured with PrestoBlue 24 h after irradiation. 5FU treatment (100 μM) and untreated cells (0.1% v/v DMSO) were used as positive and negative controls. Cells treated with prodrug **8** (100 μM) in the absence of **lipo-Pd@Au** were also used as a negative control. As shown in Figure 6b, the incubation of HCT116 cells pretreated with **lipo-Pd@Au** and prodrug **8** plus NIR irradiation (treatment E) induced highly potent inhibition of cancer cell proliferation, displaying an anticancer activity superior to that led by the photothermal effects of **lipo-Pd@Au** + NIR (treatment B) and the direct cytotoxic effects of 5FU (treatment D). Of note, incubation of HCT116 cells pretreated with **lipo-Pd@Au** and prodrug **8** in the dark (treatment C) induced lower cytotoxic levels than 5FU treatment, indicating incomplete prodrug activation in the absence of NIR stimulation. Interestingly, incubation of prodrug **8** with nonmalignant MCF-10A cells pretreated with **lipo-Pd@Au** did not show a significant reduction of cell viability, in agreement with their reduced internalization of NPs in this cell line (**SI, Figure S17**).

**Figure 6:**
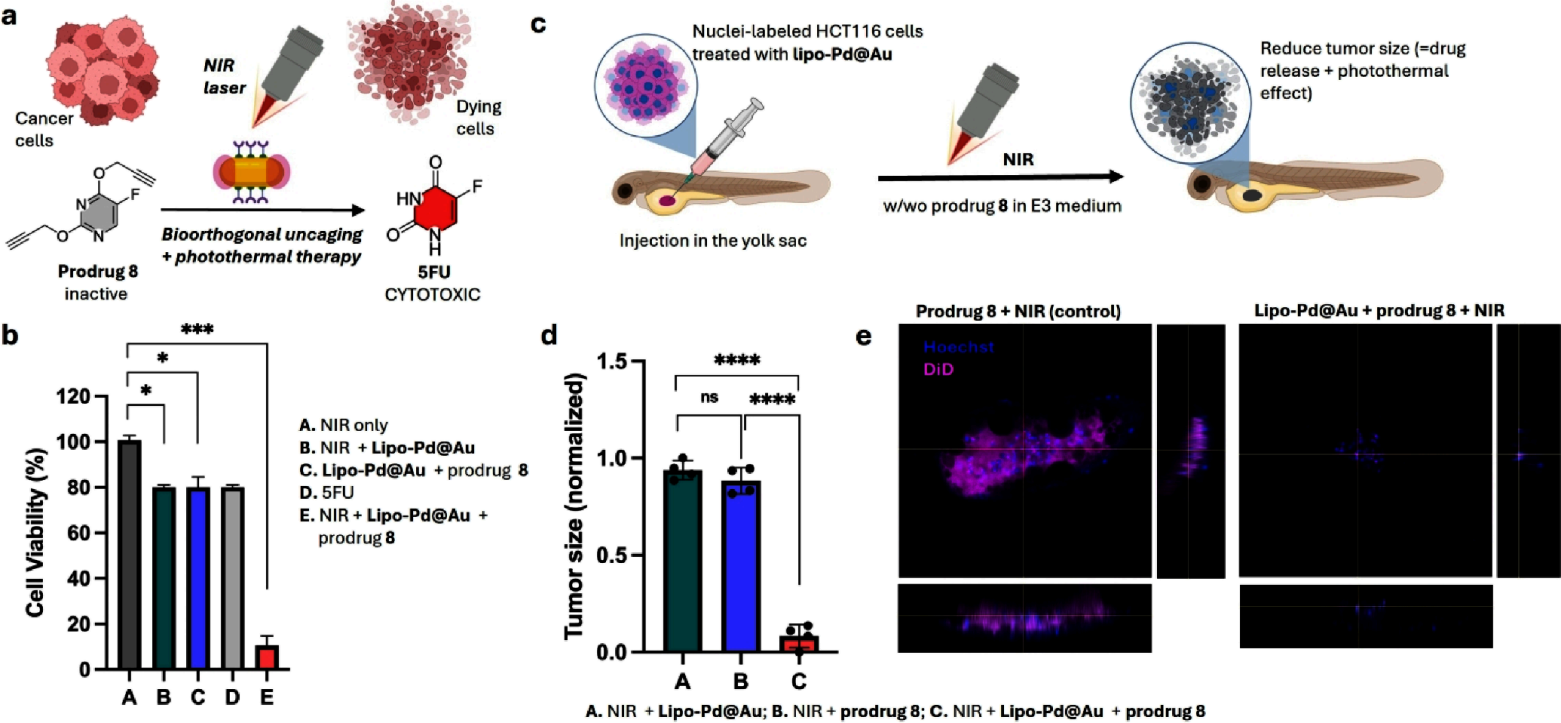
(a) Combined photothermal chemotherapy by plasmonic effects and **lipo-Pd@Au**-mediated conversion of prodrug **8** into **5FU** after NIR irradiation. (b) Cell viability assay in HCT116 colon cancer cells under different treatment conditions. [Prodrug/drug] = 100 μM. Cells were pretreated with **lipo-Pd@Au** for 6 h before prodrug addition, followed by 5 min of NIR irradiation. PrestoBlue viability assay was performed 24 h after NIR irradiation. (c) Schematic representation of the combined therapy using HCT116 xenograft zebrafish larvae model. HCT116 cells are preincubated with plasmonic **lipo-Pd@Au** when indicated and fluorescently stained with lipophilic DiD (magenta) for labeling membrane and Hoechst 33342 (cyan) for labeling nuclei. Cells are injected into the yolk sac in 2-dpf zebrafish larvae, randomly distributed into treatment groups, and incubated with prodrug **8** with or without irradiation NIR. (d) Measurement of tumor size between groups after treatment. (e) Confocal analysis of the HCT116 xenograft (DiD, magenta; Hoechst 33342, cyan) of zebrafish larvae without (left) or with **lipo-Pd@Au** (right) and treated with prodrug **8** in E3 medium followed by NIR irradiation for 30 s. Images were taken at 5-dpf. *N* = 4. Statistical analysis was performed using one-way ANOVA followed by Tukey’s posthoc test or by Dunnett′s multiple comparisons test where appropriate. Statistical results: ns >0.05, *P ≤ 0.05, **P ≤ 0.01, ***P ≤ 0.001; ****P < 0.0001 (ANOVA). **a** and **c,** were created with BioRender.com.

The combination strategy, i.e., plasmonic catalyst + bioorthogonal prodrug + NIR irradiation, was finally tested *in vivo* in the HCT116 xenograft zebrafish larvae model (Figure 6c). Following the protocol used in the fluorogenic study, HCT116 cells were preincubated with **lipo-Pd@Au** for 6 h and treated with Hoechst 33342 and DiD to fluorescently label cell nuclei and membranes, respectively. Then, cells were implanted in the PVS of 2 day old zebrafish embryos, and prodrug **8** (100 μM) was added to the medium, followed by NIR-laser irradiation for 30 s. Experiments without **lipo-Pd@Au** or without NIR irradiation were used as controls. As shown in Figure 6d,e, the combined use of the three-component strategy led to substantial tumor reduction compared to the xenograft treated only with prodrug **8** and NIR irradiation. This proof-of-concept study suggests that plasmon-catalytic devices and caged cytotoxic drugs can be combined to induce tumor cell death synergistically under NIR-laser irradiation.

### Challenging the Pd-Activatable Potential of the POxOC Masking Group

Encouraged by the fast uncaging kinetics of POxOC-masked prodye **7b** treated with bimetallic NPs in the dark (Figure 4e), we envisaged that POxOC-masked prodrugs could be suited for **lipo-Pd@Au**-mediated activation without the need for NIR stimulation. To investigate this, we prepared three prodrugs of the amino-containing chemotherapy drug doxorubicin (**DOX**): POC-protected **9a**, POxOC-protected **9b**, and 4-PBC-protected **9c** (Figure 7a, see syntheses in the SI). Among the prodrugs, it is important to note that **9b** showed higher water solubility than the other two (>1 mg/mL for **9b** versus <0.4 mg/mL for **9a** and **9c**, see Figure S18 of the SI), a valuable feature for *in vivo* use. The DOX precursors (100 μM) were then treated with **lipo-Pd@Au** (80 μg/mL) at 37 °C in 1% DMSO + 10% PBS/water (no laser irradiation), and the prodrug-to-drug conversion efficiency was measured at 0.5, 1, 2, 4, and 8 h (SI, Figures S19–S22). To our delight, **9b** was rapidly converted into DOX (Figure 7b), further supporting the idea that NIR irradiation may not be essential to uncage the POxOC group with **lipo-Pd@Au**. The conversion kinetics of **9b** were far superior to those of **9a** and **9c**, prodrugs that —on the contrary—can benefit from NIR irradiation to accelerate DOX release. Under NIR irradiation, **9b** conversion into DOX by **lipo-Pd@Au** was vastly accelerated, requiring 5 min to achieve >60% conversion (SI, Figure S23).

**Figure 7:**
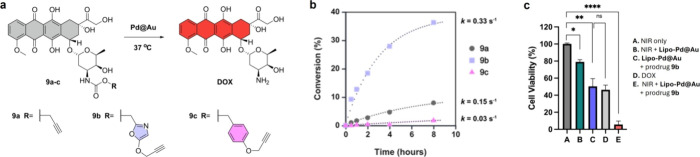
(a) **Lipo-Pd@Au**-mediated conversion of prodrugs **9a–c** into **DOX** under physiological conditions. (b) Study of the conversion kinetics for each of the prodrugs. (c) Cell viability assay in MDA-MB-231 breast cancer cells under different treatment conditions. Cells were pretreated with **lipo-Pd@Au** for 6 h before prodrug addition, followed by 5 min of NIR irradiation. PrestoBlue viability assay was performed 24 h after NIR irradiation. Error bars: ± SEM, *n* = 3. Statistical analysis: one-way ANOVA followed by Tukey’s posthoc test: ns >0.05; *P < 0.05; **P < 0.01; ****P < 0.0001.

Before performing the prodrug activation studies in cancer cell culture, the bioactivity window between prodrugs **9b** and **DOX** was determined in MDA-MB-231 cells. As shown in Figure S24 (SI), > 30-fold reduction in antiproliferative activity was observed for **9b** compared to **DOX**, providing a suitable therapeutic window to study *in situ* prodrug activation by **lipo-Pd@Au**. Next, we investigated the activation of prodrug **9b** by **lipo-Pd@Au** in cancer cell cultures with and without NIR stimulation. Since **DOX** is clinically used in triple negative breast cancer treatment, **lipo-Pd@Au** (80 μg/mL) was incubated with breast cancer MDA-MB-231 cells for 6 h, and the excess of noninternalized nanodevices was washed off, followed by **9b** (10 μM) treatment with or without 5 min NIR irradiation. Cell viability was measured with PrestoBlue after 24 h. **DOX** (10 μM) and untreated cells (0.1%, v/v, DMSO) were used as positive and negative controls. As predicted, the incubation of breast cancer cells pretreated with **lipo-Pd@Au** with prodrug **9b** (treatment C) led to an equivalent antiproliferative effect as the direct treatment with **DOX** (treatment D), corroborating the rapid activation of **9b** (Figure 7c). Further stimulation by NIR irradiation (treatment E) induced enhanced inhibition of cancer cell proliferation, as expected by the added photothermal effect generated by the nanodevices under NIR light. Complete study conditions and controls, including comparative experiments with prodrug **9a**, are described in the SI (Figure S25).

Finally, the activation of prodrug **9b** was tested *in vivo* in an MDA-MB-231 xenograft zebrafish embryo model (Figure 8a). Breast cancer cells were preincubated with **lipo-Pd@Au** for 6 h and labeled with Hoechst 33342 and DiD to facilitate the analysis of the xenografts. Implantation of the NP-containing cells in 2 day old zebrafish embryos was followed by the addition of prodrug **9b** (10 μM) to the E3 medium and incubation for 2 days. Experiments without **lipo-Pd@Au** were used as controls. Notably, even in the absence of NIR stimulation, cotreatment of **lipo-Pd@Au** and prodrug **9b** (treatments D) led to a significant reduction of tumor growth (Figure 8b–e). Instead, if one of these reagents were not present in the experiment (treatments B and C), minimal to no change in tumor growth was observed compared to the untreated animal control (treatment A). This study proves the excellent Pd-activatable properties of the POxOC masking group.

**Figure 8:**
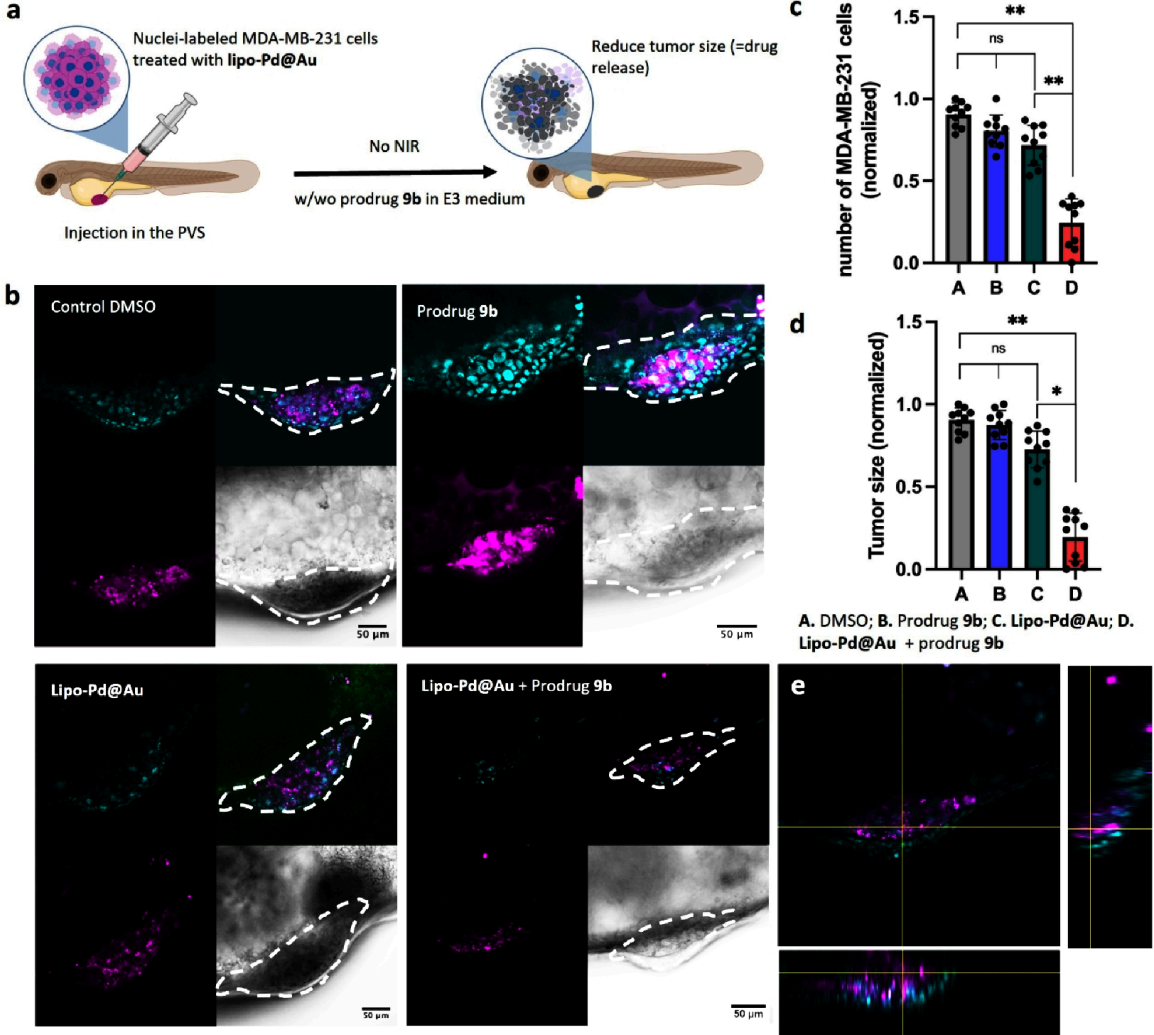
(a) Pd-mediated prodrug activation in an MDA-MB-231 breast cancer xenograft zebrafish embryo model. Cells were treated with **lipo-Pd@Au**, fluorescently stained with lipophilic DiD (magenta, membrane) and Hoechst 33342 (cyan, nuclei), and injected into the PVS in 2-dpf zebrafish larvae. Zebrafish were randomly distributed into treatment groups and incubated with prodrug **9b** for 2 d. (b) Confocal analysis of the MDA-MB-231 xenograft of zebrafish larvae without or with **lipo-Pd@Au** and treated with DMSO or prodrug **9b** in E3 medium (*n* = 10). Images were taken at 4-dpf. Tumor volumes are highlighted by white dotted lines. (c) Measurement of tumor size between groups after treatment. (d) Analysis of the number of nuclei-labeled cancer cells between groups after treatment. (e) Z-stack image of the MDA-MB-231 xenograft (DiD, magenta; Hoechst 33342, cyan) of zebrafish larvae with **lipo-Pd@Au** and treated with prodrug **9b** in E3 medium. Statistical analysis: one-way ANOVA followed by Tukey’s posthoc test: ns >0.05, *P ≤ 0.05, **P ≤ 0.01. Panel (a) was created with BioRender.com.

## Conclusions

With the aim of developing bioorthogonal tools whose catalytic activity can be remotely activated by noninvasive NIR irradiation, we engineered plasmonic bimetallic (**Pd@Au**) nanodevices and optimized their catalytic performance by Pd deposition and by protecting the exposed Au area with a thiol-functionalized phospholipid. Triggered by surface plasmon decay and electron transfer from Au to Pd, NIR-laser irradiation effectively accelerated depropargylation reactions under biocompatible conditions, achieving close to completion yields in minutes (about 100-fold increment in the reaction rate) even in the presence of serum proteins. The hyperthermia generated at the surface and vicinity of the nanomaterial promotes the reaction kinetics,^48,51^ thereby stimulating the catalyst performance. Biological studies demonstrated the capacity of the nanodevices to kill cancer cells in culture and *in vivo* by the synergic activities provided by chemotherapy activation and photothermal ablation, both processes being simultaneously triggered upon NIR irradiation. In addition, we developed a novel Pd-activatable masking group, POxOC, which increases the water solubility of the caged precursors and accelerates the bioorthogonal activation rate of prodyes and prodrugs mediated by **lipo-Pd@Au**. This new chemical cage makes the catalytic NPs suitable for bioorthogonal drug release in both the presence and absence of NIR-laser irradiation. Our studies suggest that the choice of the mask for the prodrug not only can modify the PK properties of the reagent but also can be an important factor to either mediate constant drug release (prodrugs with fast activation kinetics in the dark, e.g., POxOC) or implement a photocontrolled activation strategy (prodrugs with slow activation kinetics in the dark, e.g., 4-PBC). This investigation expands the scope of bioorthogonal catalysis and opens new avenues for the design of nanozymes, prodrugs, and phototherapeutic interventions on demand in a spatiotemporally controlled fashion.

## Supplementary Material



## Notes

Detailed materials and methods, nanomaterials characterization data, compound synthesis and characterization, ^1^H and ^13^C NMR spectra, experimental procedures for biological and *in vivo* studies, supplementary figure, and references, including Schemes S1 and S2, Table S1, and Figures S1–S25 (PDF)

## Abbreviations

5FU: 5-fluorouracil
DOX: doxorubicin
FDA: Food and Drug Administration
NPs: nanoparticles
HAADF: high-angle annular dark-field
(S)­TEM: (scanning) transmission electron microscopy
EDS: energy-dispersive X-ray spectroscopy
ICP-MS: inductively coupled plasma mass spectrometry
HR-TEM: high-resolution transmission electron microscopy
NIR: near infrared
POC: propargyloxycarbonyl group
POxOC: 2-propargyloxyoxazole-4-methyl)­oxycarbonyl group
4-PBC: 4-(propargyloxy)­benzyloxycarbonyl group
