# Supplementary material for: Near-Infrared Light-Accelerated Bioorthogonal Drug Uncaging and Photothermal Ablation by Anisotropic Pd@Au Plasmonic Nanorods
Source: J Am Chem Soc. 2025 Jun 26;147(27):23980–90. doi: 10.1021/jacs.5c07261 (PMC12257503; doi:10.1021/jacs.5c07261)
Supplement: Supplementary file 1 [file ja5c07261_si_001.pdf]

Supporting Information

NIR light-accelerated bioorthogonal drug uncaging and photothermal ablation by anisotropic Pd@Au plasmonic nanorods.

M. Carmen Ortega-Liebana, [⊥,‡,≠,†,*] Jana Travnickova,[‡,#] Catherine Adam,[‡] Davir González-Calderón,[‡] Álvaro Lorente-Macías,[‡] Charles Lochenie,[≤] Raul Arenal,[§,¥,±] E. Elizabeth Patton[‡,#] and Asier Unciti-Broceta[‡,*]

AUTHOR ADDRESS.

[⊥] Department of Medicinal and Organic Chemistry and Unit of Excellence in Chemistry Applied to Biomedicine and Environment, Faculty of Pharmacy, Campus Cartuja s/n, University of Granada, 18071 Granada, Spain.

[‡] Edinburgh Cancer Research, Cancer Research UK Scotland Centre, Institute of Genetics and Cancer, University of Edinburgh, Crewe Road South, EH4 2XU Edinburgh, UK.

[≠] GENYO, Pfizer/University of Granada/Andalusian Regional Government, Avda. Ilustración 114, 18016 Granada, Spain.

[†] Instituto de Investigación Biosanitaria ibs.GRANADA, Granada, Spain.

[#] MRC Human Genetics Unit, Institute of Genetics and Cancer, University of Edinburgh, Edinburgh EH4 2XR, UK.

[≤] Pandemic Science Hub, Institute for Regeneration and Repair, University of Edinburgh, EH16 4UU Edinburgh, UK.

[§] Laboratorio de Microscopias Avanzadas (LMA), Universidad de Zaragoza, 50018 Zaragoza, Spain.

[¥] Instituto de Nanociencia y Materiales de Aragon (INMA), CSIC– University of Zaragoza, 50009 Zaragoza, Spain.

[±] ARAID Foundation, 50018 Zaragoza, Spain.

*correspondence should be addressed to: asier.ub@ed.ac.uk (A.U.B.) or mcortega@ugr.es (M.C.O.L.)

Table of Contents

1. **General Information** ……………………………………………………………………S2

2. **Experimental Procedures and characterizations** ……………………………………..S3

3. **Biological studies** ……………………………………………………………………….S27

4. ***In vivo* experiments** ………………………………………………………………….…S29

5. **Supplementary References** ……………………..……………………….……………S31

6. **Supplementary Figures** ……………..………………………………………………….S32

1. GENERAL INFORMATION

Materials. Chemical and solvents were purchased from Fisher Scientific, Sigma-Aldrich or VWR International Ltd. Resorufin (95% purity), ethyl 2-chlorooxazole-5-carboxylate (**2,** 95% purity) and 5-bromosalicylic acid (90% purity) are commercially available and were purchased from Fluorochem UK. 4-Chloro-7-nitrobenzofurazan (NBD-Cl, 98% purity) was purchased from Alfa Aesar. Gold(III) chloride hydrate (99.9% purity), Hexadecyltrimethylammonium bromide (CTAB, 96% purity), Hexadecyltrimethylammonium chloride (CTAC, 98% purity), sodium borohydride (99,99% purity), L-ascorbic acid (99% purity), 5-bromosalicylic acid (90% purity), silver nitrate (99.9999% purity), Doxorubicin hydrochloride (DOX, 98-102% purity) and 5-Fluorouracil (5-FU, ≥99% purity) were purchased from Sigma-Aldrich. Thiol-terminated 1,2-distearoyl-sn-glycero-3-phosphethanolamine-poly (ethylene glycol) (DSPE-PEG-SH, 1 kDa) was obtained from Abbexa Ltd. **Pro-Res**, **POC-NBD (prodye 7a)**, **prodrug 8** and **9c** was synthesized as previously reported.[1-3]

Characterization. NMR spectra were recorded at 300 K on a 500 MHz Bruker Avance III HD spectrometer. Chemical shifts are reported in parts per million (ppm) relative to the solvent peaks. Data are presented as follows: chemical shift (ppm), multiplicity (s = singlet, d = doublet, t = triplet, m = multiplet, q= quartet), coupling constant *J*, and integration. R_f values were determined on Merck TLC Silica gel 60 F_{254} plates under a 254 nm UV source. Purifications were carried out by flash column chromatography using commercially available Biotage® Sfär silica column (60 µm particle size - 10 g). High-Resolution Mass Spectrometry was performed with a Bruker MicroTOF focus II mass spectrometer. All the compound used in cell and zebrafish studies were >99% pure, as measured by HPLC using an Agilent 1260 Infinity II Preparative LC/MSD system coupled to an Evaporative Light Scattering detector (ELSD). Prodrug-into-drug conversion experiments were conducted using the same HPLC equipment. Millimolar stock solutions of the prodrugs/prodyes were prepared in DMSO. The optical properties of plasmonic nanorods were analyzed by a NanoDrop™ 2000c spectrophotometer (Thermo Scientific™), and the ratio amount of Au and Pd of the plasmonic nanorods was determined by ICP-MS (Agilent 7500ce). Samples were previously digested, with a mixture of aqua regia (2%) and distilled water. The hydrodynamic diameter of the nanoparticles was determined by DLS using a Malvern Zetasizer Nano-S.

Electron microscopy analyses. Scanning transmission electron microscopy (STEM) and energy dispersive X-ray spectroscopy (EDS) measurements have been performed using a Thermo Fisher Scientific Titan Low-Base probe-corrected microscope. The analyses were conducted at 80 kV and at 300 kV in the Titan Low-Base using also high-angle annular dark field (HAADF)-STEM imaging. High resolution TEM images have been acquired using the Thermo Fisher Scientific Titan Cube Cs-corrected microscope, working at 300 kV.

Near-infrared (NIR) laser irradiation set-up. The NIR irradiation set-up consisted of an 808 nm laser module with 400 µm@1m fiber coupling (MXL-III model; Changchun New Industries Optoelectronics Technology Co., Ltd. = CNI laser) connected to a fixed focus collimator (SMA905 model; CNI laser). The laser diode was driven using a variable power supply unit (PSU-III-LED model, CNI laser). The photothermal effect under NIR irradiation was performed using an experimental setup based on a laser diode emitting at 808 nm. Temperature changes during NIR irradiation were monitored by an infrared thermometer with a trigger grip and a thermocouple jack (Fisherbrand™).

2. SYNTHESES AND CHARACTERIZATIONS

Synthesis of gold nanorods. Au nanorods were prepared following the seed-mediated method.[4] To prepare gold seeds, a $HAuCl_4{\cdot}3H_2O$ solution (29 µL, 0.086 M) was added into the CTAB solution (10 mL, 0.1 M) with vigorous stirring. An ice-cold freshly prepared $NaBH_4$ solution (0.6 mL, 0.01 M) was then injected. The mixture changed from yellow to brown, indicating the creation of nuclear gold. After 5 min of stirring, the solution was stored for 1 h before use.
The growth solution was prepared by dissolving CTAB (3.6 g) and 5-bromosalicylic acid (0.44 g) in 100 mL of warm water (Millipore, 55 °C). To this solution, 1.92 mL of 0.01 M fresh $AgNO_3$ was added, and after keeping it undisturbed at 25 °C for 15 min, 100 mL of 1 mM $HAuCl_4{\cdot}3H_2O$ solution was added. After gentle mixing of the solution for 15 min, 0.512 mL of 0.1 M ascorbic acid (AA) was added under vigorously stirring for 30 s until the mixture became colourless. At this point, 0.32 mL of seed solution was added to the entire growth solution. The mixture was stirred for 30 s and left undisturbed at 27 °C for 12 h. The colour of the growth solution slowly changed from colourless to violet (growth of Au nanorods) and then to a deep wine-red colour, indicating the formation of Au nanorods. The as-made Au nanorods were labelled as **Au-1** and used for subsequent Pd metal incorporation onto this Au nanorods template without purification treatment. Another Au nanorods template, labelled as **Au-2**, was also made following a similar procedure except eliminating 5-bromosalicylic acid and increasing the concentration of CTAB to 0.1 M.

Synthesis of Pd@Au nanoparticles. Pd@Au NPs were prepared as previously described[5, 6] with slight modifications. H_2PdCl_4 solution (0.01 M) was prepared by dissolving 0.089 g of $PdCl_2$ powder in 50 mL of 0.02 M HCl solution at 60 °C (stable solution for more than 1 year). 15 mL glass vial was cleaned with aqua regia to dissolve and lift any traces of metal deposits off the glass surfaces. **Pd-tipped (NP1)** were prepared by using **Au-1 nanorods** as a template. Briefly, 1.136 mL of 0.1 M ascorbic acid was added into 10 mL of as-made Au nanorods suspension. Then, 198 µL of 0.01 M H_2PdCl_4 and subsequently 80 µL of 0.1 M HCl were added to the reaction mixture. The mixture was left undisturbed for 12 h at 27 °C. **Pd-shelled (NP2)** were prepared by using **Au-2 nanorods** as a template following the same procedure. Finally, to synthesize **Pd-spotted (NP3)**, the **Au-1 nanorods** stock solution (200 µL) was added to the CTAC solution (3 mL, 0.025 M). An H_2PdCl_4 solution (26 µL, 10 mM) and AA solution (13 µL, 0.1 M) were added sequentially, and the mixture was kept undisturbed at 25 °C for 0.5 h (see **scheme S1**).

Scheme S1. Schematic illustration of the formation of Pd@Au nanoparticles using different Pd metal deposition methods onto plasmonic Au nanorods by adjusting one or two reaction parameters.

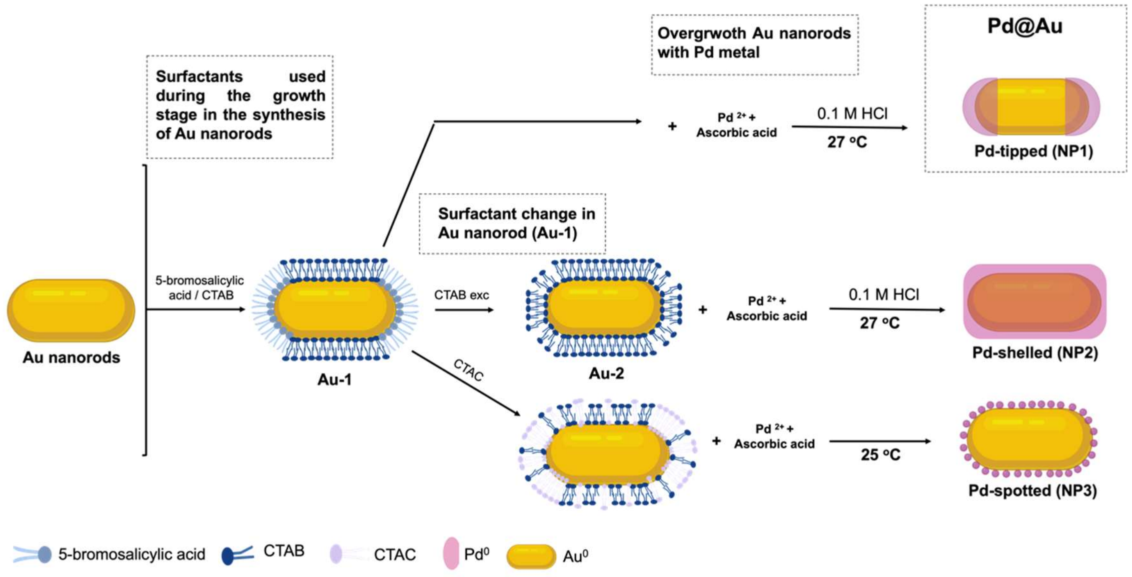

Subsequently, all plasmonic NPs were collected by centrifugation (10 min at 8000 rpm), washed several times with Milli-Q water, and re-dispersed in water until further use. Monodisperse plasmonic NPs with different characteristics were prepared and analyzed by DLS, UV-vis and HAADF-STEM. Full characterization of plasmonic NPs is shown in **Figure S1, S3-5**.

The catalytic properties of **Au nanorods**, **NP1, NP2** and **NP3** described above were tested using the off/on fluorescent probe **Pro-Res**, which upon *O*-propargyl cleavage releases strongly fluorescent resorufin. For this screening, a concentration of nanoparticles of 40 μg/mL was added to an aqueous solution of **Pro-Res** at 100 μM. Either PBS or PBS + 10% of serum (FBS) were used as reaction media. The photoactivation properties for metal-mediate O-propargyl cleavage of the NPs were evaluated under laser irradiation using an 808 nm laser diode (model MDL-III-808-2W), coupled to an optical fiber of 400 μm of diameter and a fixed focus collimator (Changchun New Industries Optoelectronics Technology Co., Ltd., Changchun, P. R. China). Reactions were either irradiated for 15 min, operating at 1 W cm^{-2} while keeping a room temperature or were shaken at 700 rpm and 37 °C in a Thermomixer for 15 min and after 24 h to compare the effect of irradiation and non-irradiation. Fluorescence intensities were analysed in a PerkinElmer EnVision 2101 multilabel reader (Ex/Em: 550 /580 nm). Samples were repeated in triplicate. The results are shown in **Figure S2**. The anisotropic heterostructure of **NP1** demonstrated superior catalytic properties in comparison with **NP2**, **NP3** and the **Au nanorods** under the same conditions.

Based on these the fluorogenic studies, **NP1** (named **Pd@Au** in the main manuscript) were selected for the further in vitro and in vivo experiments. Subsequently, the resulting **Pd@Au** were engineered with a thiol-functionalized PEGylated phospholipid (DSPE-PEG-SH), which has both hydrophilicity and hydrophobility, to obtain **lipo-Pd@Au** using an excess of DSPE-PEG-SH. A solution of **Pd@Au** was brought into contact with a dilution of DSPE-PEG-SH during one hour under magnetic stirring. Any excess of unbound DSPE-PEG-SH was removed by dialysis. The concentration of the final dispersion was adjusted by centrifugation of 10.000 rpm for 10 minutes.

Table S1. Quantification of Au and Pd content by inductively coupled plasma mass spectrometry (ICP-MS). Plasmonic NPs of different batches (0.2 mg of sample) were digested with 1 mL of freshly-prepared aqua regia and the samples were left for digestion for 30 min. Afterwards, 40 μL of each sample plus 40 μL of aqua regia was diluted to 4 mL with Mili-Q water to achieve a final concentration of acid of 2 % *v/v*. Au/Pd quantification was done on a Agilent 7500ce ICP-MS. Calibrations were performed employing Au/Pd standards in the same background solution (2 % aqua regia) with excellent correlations. Samples were measured in triplicate.

Sample	ICP-MS 197 Au [He] (ppb)	% rsd	ICP-MS 105 Pd [He] (ppb)	% rsd	Pd % *wt/wt*
**Au nanorods**	1.577	1.5%	0.00		-
**Au nanorods**	1.897	2%	0.00		-
**Mean**					**0 % Pd**
**Pd-tipped (NP1)**	163,102	0.4%	65.296	1.1%	28.59%
**Pd-tipped (NP1)**	158,632	2.6%	61.537	1.1%	27.95%
**Mean**					**28.3 % Pd**
**Pd-shelled (NP2)**	145.026	0.2%	52.715	2.8%	29.59%
**Pd-shelled (NP2)**	138.24	1.5%	48,482	1.2%	28,80%
**Mean**					**29.2 % Pd**
**Pd-spotted (NP3)**	433.543	1.7%	123.265	1%	22,14%
**Pd-spotted (NP3)**	445.574	1.2%	116.167	0.8%	20,68%
**Mean**					**21,41 % Pd**
Blank	0.000				
1 ppb AuPd Check	0.964	4.4%			
10 ppm AuPd Check	9.627	1.7%			

Synthesis of Pro-Res

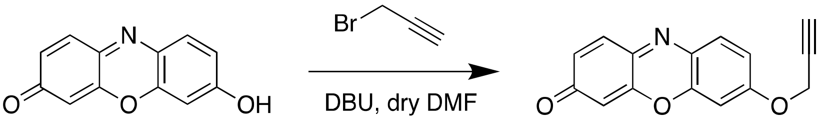

Pro-Res was synthesized as previously reported.[2]
^{1}H NMR (500 MHz, DMSO-d$_6$) δ = 7.82 (d, J = 8.9 Hz, 1H), 7.55 (d, J = 9.9 Hz, 1H), 7.18 (d, J =2.7 Hz, 1H), 7.11 (dd, J = 8.9, 2.7 Hz, 1H), 6.81 (dd, J = 9.8, 2.1 Hz, 1H), 6.30 (d, J = 2.0 Hz, 1H), 5.01(d, J = 2.4Hz, 2H),3.70 (t, J = 2.5 Hz, 1H). NMR data was in accordance with the literature.[2]

Synthesis of prop-2-yn-1-yl ethyl(7-nitrobenzo[c][1,2,5]oxadiazol-4-yl)carbamate (POC-NBD, prodye 7a)

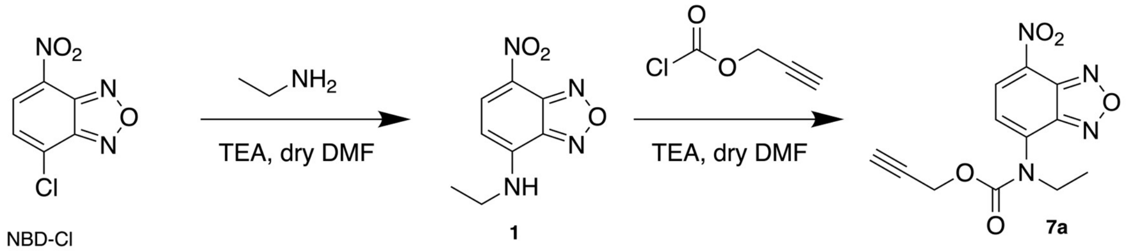

Poc-NBD (7a) was synthesized as previously reported.[2]
Compound 1: ^{1}H NMR (500 MHz, DMSO-d$_6$) δ = 9.50 (s, 1H), 8.49 (d, J = 10 Hz, 1H), 6.38 (d, J =10 Hz, 1H), 3.51 (s, 2H), 1.27 (t, J = 5 Hz, 3H) ppm. **^{13}C NMR** (126 MHz, DMSO-d$_6$) δ = 144.9, 144.4, 144.1, 120.5, 98.9, 38.2, 12.3 ppm. **HRMS (ESI) (*m/z*)**: [M]$^+$ calcd. for $C_8H_8N_4O_3$, 208.06; found, 209.06 [M + H]$^+$ and 231.06 [M + Na]$^+$. **Compound 7a: ^{1}H NMR** (500 MHz, DMSO-d$_6$) δ = 8.73 (d, J = 10 Hz, 1H), 7.40 (d, J = 5 Hz, 2H), 7.78 (d, J =5 Hz, 1H), 4.78 (d, J = 2.5 Hz, 2H), 3.94 (q, J = 7 Hz, 2H), 3.56 (t, J = 2.5 Hz, 1H), 1.18 (t, J = 7 Hz, 3H) ppm. **^{13}C NMR** (126 MHz, DMSO-d$_6$) δ = 152.8, 147.9, 143.7, 136.6, 134.1, 133.0, 126.5, 78.1, 78, 53.8, 45.4, 13.6 ppm. **HRMS (ESI) (*m/z*)**: [M]$^+$ calcd. for $C_{12}H_{10}N_4O_5$, 290.07; found, 291.0754 [M + H]$^+$ and 313.0642 [M + Na]$^+$. NMR and HRMS data were in accordance with the literature.[2]

Synthesis of POxOC-NBD, prodye 7b: (2-(prop-2-yn-1-yloxy)oxazol-5-yl)methyl ethyl(7-nitrobenzo[c][1,2,5]oxadiazol-4-yl)carbamate

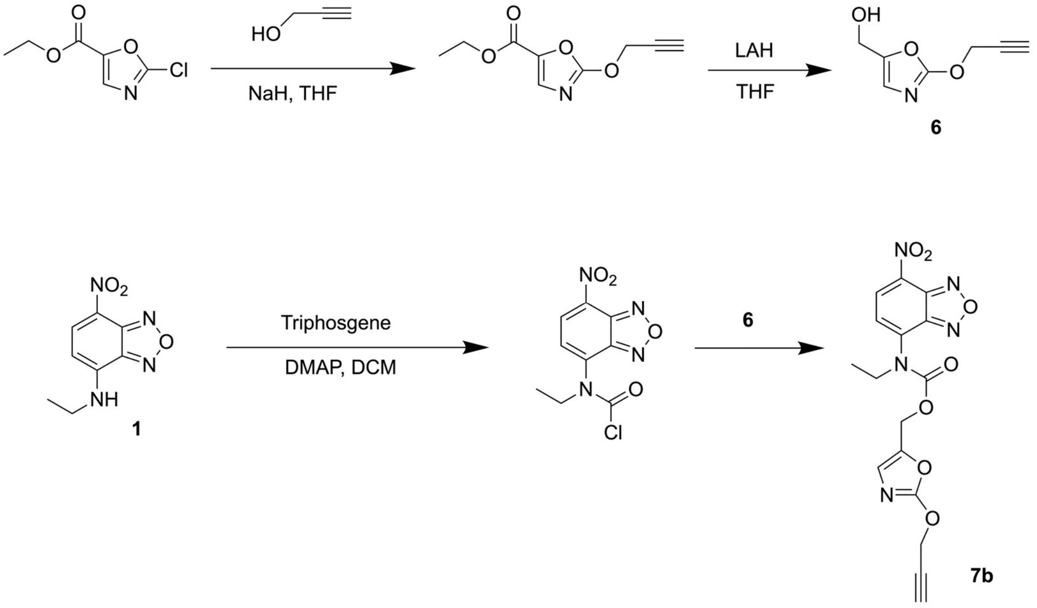

Synthesis of compound 6. To a solution of propargyl alcohol (888.3 uL, 15.4 mmol, 9 eq) in THF (9 mL) was added NaH (205 mg, 5.12 mmol, 60% purity, 3 eq) in batches at 0 °C. The mixture was stirred at this temperature for 1 hr, ethyl 2-chlorooxazole-5-carboxylate (300 mg, 1.71 mmol, 1 eq) was then added. The mixture was stirred at 20 °C for 1 hr. The reaction mixture was adjusted to pH 6 with aqueous HCl (1 N, 150 mL), and then extracted with EtOAc (60 mL x 2). The combined organic layer was washed with brine (50 mL), dried over Na_2SO_4, and then filtered and concentrated under reduced pressure. Ethyl 2-(prop-2-yn-1-yloxy)oxazole-5-carboxylate (0.32 mg, crude) was obtained as a yellow oil. The crude product was used for the next step without further purification. To an ice-cooled solution of ethyl 2-(prop-2-yn-1-yloxy)oxazole-5-carboxylate (200 mg, 1.02 mmol, 1 eq) in dried THF (0.5 ml) was added LAH (44 mg, 1.2 mmol, 1.1 eq) in batches. The reaction mixture was stirred at 0-20 °C for 1h. TLC (Petroleum ether: EtOAc = 3:1) showed one spot with higher polarity. The reaction mixture was quenched with water (0.2 mL), NaOH (15% in water, 0.2 mL) and water (0.7 mL). The mixture was then filtered. The filter cake was washed with EtOAc (20 mL x 5). The combined filtrate was dried over Na_2SO_4, and then filtered and concentrated under reduced pressure. Compound **6** (140.1 mg, 0.915 mmol, 89% yield) was obtained as a yellow oil, which was used fort he next step without purification. **LRMS (ESI) (*m/z*)**: $[M]^+$ calcd. for $C_7H_7NO_3$, 153.14; found, 154.00 $[M + H]^+$.

Synthesis of prodye 7b. Compound **1** (25 mg, 0.12 mmol, 1 eq) and DMAP (36.5 mg, 0.30 mmol, 2.5 eq) were dissolved in dry DCM (2 ml) under nitrogen atmosphere. The reaction was cooled to 0 °C using an ice bath before a triphosgene solution (21.4 mg, 0.07 mmol, 0.6 eq) in toluene was added dropwise. The reaction mixture was allowed warm to room temperature and stirred for 4h. The reaction mixture was reduced to dryness by bubbling with N_2 before the residue was redissolved in anhydrous DCM and cooled to 0 °C (2 mL). Compound **5** (27.6 mg, 0.18 mmol, 1.5 eq) in 1 mL of anhydrous DCM was added dropwise. The reaction was allowed to return to room temperature and stirred under nitrogen atmosphere. The reaction was quenched with water and extracted with DCM. The resulting organic layer was dried over $MgSO_4$, filtered and removed in vacuo to afford the crude product as a yellow oil. The crude was purified *via* semipreparative TLC chromatography (30% EtOAc in hexane) to yield prodye **7b** as a yellow solid (31.7 mg, 0.12 mmol, 98% yield).

^{1}H NMR (500 MHz, DMSO-d$_6$): δ = 8.70 (d, J = 7.9 Hz, 1H), 7.75 (d, J = 8.0 Hz, 1H), 6.99 (s, 1H), 5.11 (s, 2H), 5.04 (d, J = 2.5 Hz, 2H), 3.92 (q, J = 7.1 Hz, 2H), 3.72 (t, J = 2.4 Hz, 1H), 1.15 (t, J = 7.1 Hz, 3H) ppm. **^{13}C NMR** (126 MHz, DMSO-d$_6$) δ = 160.77, 153.05, 147.89, 143.62, 141.90, 136.75, 133.93, 133.04, 127.04, 125.97, 79.35, 77.39, 58.76, 57.42, 45.44, 13.61 ppm. **HRMS (ESI) (*m/z*)**: [M]$^+$ calcd. for $C_{16}H_{13}N_5O_7$, 387.080; found, 388.0851 [M + H]$^+$.

Synthesis of PBC-NBD, prodye 7c

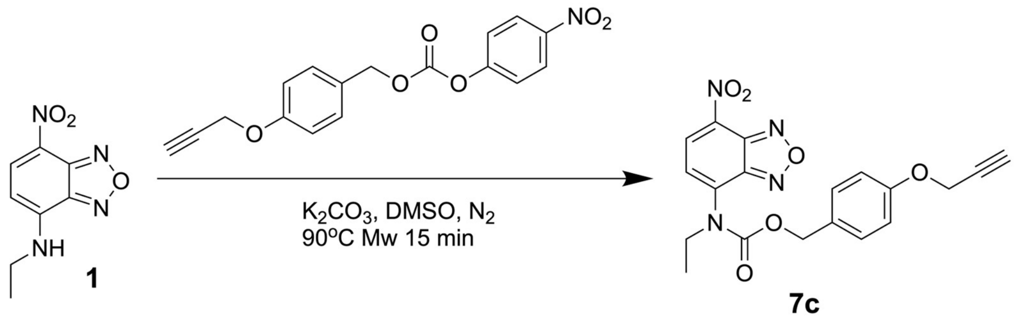

Compound **1** (200 mg, 1 mmol, 1 Eq), potassium carbonate (414 mg, 3 mmol, 3 Eq) and 4-nitrophenyl[4-(prop-2-yn-1-yloxy)phenyl]methylcarbonate[7] (490 mg, 1.5 mmol, 1.5 Eq) were placed in a 20 mL microwave vial equipped with a magnetic stirrer. The atmosphere was exchanged for N2, then 10 mL dry degassed DMSO were added. The mixture was then heated to 90°C by microwave for 15 min. After cooling to RT, **7c** was purified by preparative HPLC (XB-C18/H2O+0.1%TFA/MeCN+0.1%TFA), then the fractions lyophilised to give pure **7c** as beige powder (12 mg, 0.03 mmol, 3% yield).

^{1}H NMR (601 MHz, CDCl$_3$) δ 8.20 (d, J = 8.2 Hz, 1H), 7.37 (d, J = 8.2 Hz, 1H), 7.02 (d, J = 8.4 Hz, 2H), 6.96 (d, J = 8.4 Hz, 1H), 5.10 (m, 4H), 4.83 (qua, J = 8.1 Hz, 2H), 2.53 (s, 1H), 1.25 (t, J = 8.1 Hz, 4H).**^{13}C NMR** (151 MHz, CDCl$_3$) δ δ 163.74, 157.78, 141.67, 130.21, 129.28, 128.46, 125.95, 115.23, 114.85, 77.24, 77.03, 76.82, 70.43, 69.41, 55.88, 55.83, 29.73, 29.71. **HRMS (ESI) (*m/z*)**: [M+Na]$^+$ calcd. for $C_{19}H_{16}N_4NaO_6^+$, 419.0963; found 419.0979.

Synthesis of benzyl ethyl(7-nitrobenzo[c][1,2,5]oxadiazol-4-yl)carbamate (compound 7d)

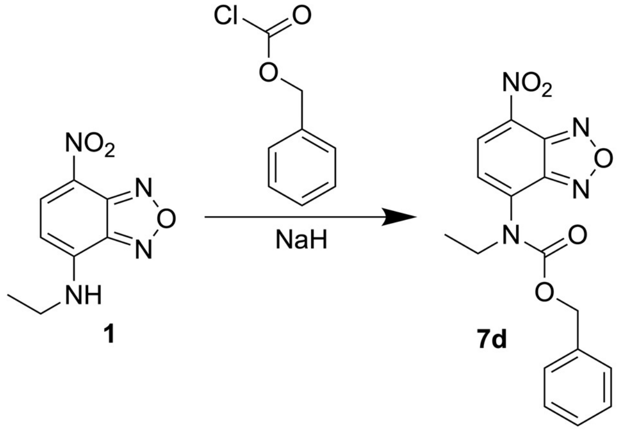

A 2 mL vial equipped with a stir bar was charged with compound **1**(10 mg, 48 µmol, 1 eq) and DMF (0.3 mL). To this solution was added NaH (3.8 mg, 60% Wt, 96 µmol, 2 eq) portionwise and the reaction stirred at room temperature for 15 minutes. Then, benzyl carbonochloridate (12 mg, 10 µL, 72 µmol, 1.5 eq) was added, the vial was sealed and the reaction stirred for 2 days at room temperature. The reaction was diluted with EtOAc, washed with water and brine.

The organic phases were dried over $MgSO_4$, filtered and concentrated under reduced pressure. The crude product was purified by flash column chromatography (Isolera, 10 g column, 15 mL/min), (EtOAc/hexane = 0 - 30%) to afford a brown oil (5.1 mg, 15 μmol, 31% yield).

^{1}H NMR (500 MHz, $CDCl_3$) δ 8.48 (d, J = 8.0 Hz, 1H), 7.50 (d, J = 8.0 Hz, 1H), 7.38 – 7.27 (m, 5H), 5.24 (s, 2H), 4.14 (q, J = 7.1 Hz, 2H), 1.28 (t, J = 7.1 Hz, 3H). **^{13}C NMR** (126 MHz, $CDCl_3$) δ 154.19, 147.67, 143.76, 138.30, 135.25, 133.82, 131.44, 128.83, 128.79, 128.51, 124.23, 68.98, 45.81, 14.36. **LRMS (ESI) (*m/z*)**: $[M]^+$ calcd. for $C_{16}H_{14}N_4O_5$, 342.31; found, 343.30 $[M + H]^+$.

Synthesis of 5-fluoro-2,4-bis(prop-2-yn-1-yloxy)pyrimidine (prodrug 8)

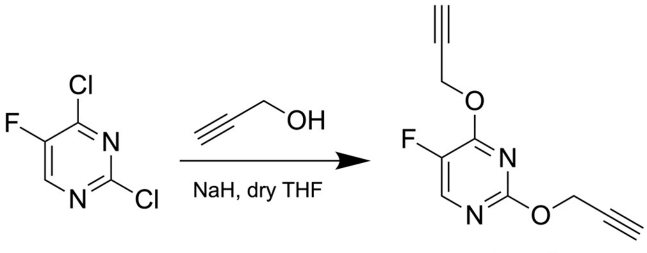

prodrug 8

Prodrug 8 was synthesized as previously reported.[1]
^{1}H NMR (500 MHz, $CDCl_3$) δ = 8.14 (d, J_{H-F} = 2.3 Hz, 1H), 5.07 (d, J = 2.4 Hz, 2H), 4.95 (d, J = 2.4 Hz, 2H), 2.54 (t, J = 2.4 Hz, 1H), 2.47 (t, J = 2.4 Hz, 1H). NMR data was in accordance with the literature.[1]
Purity: 99.6% (HPLC-MS).

Synthesis of *N*-Poc-DOX (prodrug 9a)

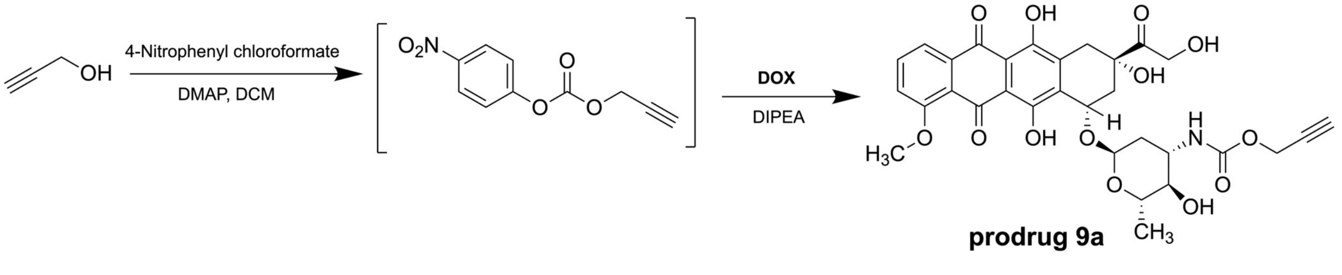

prodrug 9a

Prodrug 9a was prepared using a modified reported protocol.[8] Propagyl alcohol (31 μL, 0.535 mmol, 10 eq) and DMAP (130.8 mg, 1.07 mmol, 20 eq) was dissolved in anhydrous DCM (1 mL) and stirred at 0 °C. To the solution was added 4-nitrophenyl chloroformate (161.8 mg, 0.803 mmol, 15 eq) dissolved in 1 mL of DCM dropwise. The reaction was allowed to return to room temperature and stirred in the dark overnight. The reaction mixture was quenched with ice and extracted with DCM. The combined organic layer was washed with water and brine until no more yellow colour was observed in the organic phase, dried over $MgSO_4$ and removed in vacuo to afford the carbonate intermediate as yellow solid (93.5 mg, 0.423 mmol, 53%). Doxorubicin hydrochloride (DOX, 30 mg, 0.052 mmol, 1 eq) and DIPEA (75 μL, 0.43 mmol) were dissolved in DMF and cooled on ice. To this mixture, the carbonate intermediate in DMF (16 mg in 1 mL) was added dropwise. The reaction mixture was stirred in the dark at room temperature overnight. The mixture was diluted with DCM, washed with water and brine. The organic layers were combined, dried with $MgSO_4$, filtered, and removed in vacuo to afford red solid as crude product. Purification was carried out (0-10% MeOH in DCM) using column chromatography to afford the **9a** as a red solid (25.9 mg, 0.041 mmol, 79%).

^{1}**H NMR** (500 MHz, $CDCl_3$): δ = 13.96 (s, 1H), 13.21 (s,1H), 8.03 (d, *J* = 7.7, 1H), 7.77 (t, *J* = 8.1, 1H), 7.38 (d, *J* = 8., 1H), 5.50 (d, *J* = 3.9, 1H), 5.28 (s,1H), 5.16 (d, *J* = 8.5, 1H), 4.74 (dd, *J* = 4.8, 1.8, 2H), 4.59 (d, *J* = 2.1, 2H), 4.50 (s, 1H), 4.12 (q, *J* = 6.5, 1H), 4.06 (s, 3H), 3.85 (s, 1H), 3.65 (d, *J* = 6.4, 1H), 3.26 (dd, *J* = 18.8, 1.5, 1H), 3.02 - 2.96 (m, 2H), 2.41 (s, 1H), 2.31 (d, *J* = 14.7, 1H), 2.19 - 2.12 (m, 1H), 1.87 (dd, *J* = 16.9, 6.5, 2H), 1.75 (td, *J* = 13.2, 4.2, 1H), 1.27 (d, J = 6.6, 3H). ^{13}**C NMR** (126 MHz, $CDCl_3$): δ= 213.96, 187.30, 186.90, 161.24, 156.32, 155.83, 154.74, 135.93, 135.69, 133.72, 133.63, 121.08, 120.03, 118.62, 111.81, 111.63, 100.79, 78.23, 74.80, 69.84, 69.68, 67.36, 65.68, 56.85, 52.67, 47.25, 35.80, 34.19, 30.32, 16.97. **HRMS (ESI) (*m/z*)**: $[M]^+$ calcd. for $C_{31}H_{31}NO_{13}$, 625.18; found, 648.1677 $[M + Na]^+$. HRMS data were in accordance with the literature.[8]

Synthesis of POxOC-DOX (prodrug 9b)

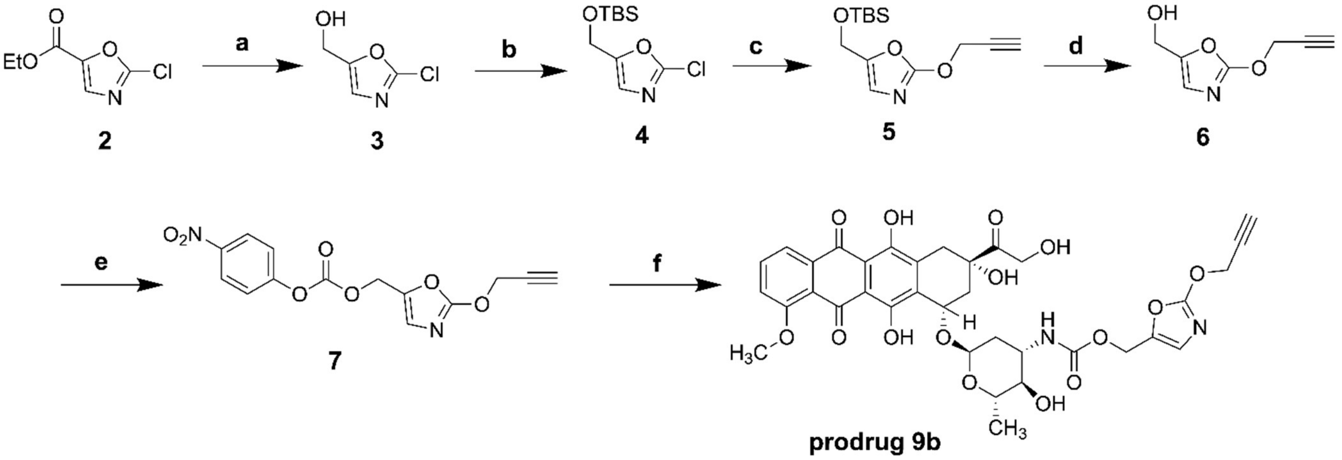

Scheme S2. Reagents and conditions: (a) DIBAL-H (3.0 eq), THF anh., -78 °C→r.t., N_2, 12h, 50%. (b) TBSCl (1.1 eq), imidazole (2.5 eq) DMF anh., r.t., N_2, 12 h, 86%. (c) Propargyl alcohol (3.0 eq), NaH (1.5 eq), THF anh., 0°C→r.t., N_2, 36 h, 90%. (d) TBAF (1.5 eq), THF, r.t., 60 min. 89%. (e) 4-nitrophenyl chloroformate (1.5 eq), pyridine anh. (1.5 eq), THF anh., r.t., N_2, 12 h, 60%. (f) Doxorubicin hydrochloride (0.83 eq), Et_3N (5.0 eq), dioxane/MeCN and. 1:1, r.t., N_2, 48 h, 49%.

To provide the proposed trigger/linker/effector core, the alcohol **5** was envisioned and prepared (**Scheme S2**) in order to introduce the carbonate precursor **6** into the drug (doxorubicin), furnishing the corresponding prodrug **7**. Commercial ethyl 2-chlorooxazole-5-carboxylate (**2**) was used as starting material. In contrast with the use of LAH (total decomposition of starting material), DIBAL-H afforded acceptable yields (50%) of alcohol **3** which was protected (**4**, 86%) to allow a high efficient insertion of propargyl alcohol (**5**, 90%). Conventional *O*-silyl cleavage, provided desired alcohol **6** (89%) which was functionalized in carbonate **8** (60%). *N*-protection of doxorubicin achieved the obtaining of prodrug **9** (49%).

(2-Chlorooxazol-5-yl)methanol (**3**): In a well-dried round-bottom flask equipped with a magnetic stirring bar was dissolved ethyl 2-chlorooxazole-5-carboxylate (**2**) (1.0 g, 5.69 mmol) in anhydrous THF (10.0 mL). After purging the reaction system with N_2, the solution was stirred at -78 °C under inert atmosphere, then diisobutylaluminum hydride (2.42 g, 17.07 mmol) solution in tetrahydrofuran was added drop-wise. The reaction mixture was stirred overnight at -78 °C allowing to reach the room temperature throughout the reaction time. The flask is placed in an ice bath and the reaction mixture was quenched saturated aqueous ammonium chloride (50.0 mL, carefully added) stirring vigorously for 15 min. The solid is filtered off washing with ethyl acetate. The organic layer is separated and dried ($MgSO_4$). The drying agent is filtered off and the solvent is evaporated under reduced pressure. Column chromatography afforded 0.37 g (50%) of desired alcohol **3** as slightly yellow liquid (thermosensitive and slightly volatile). R_f = 0.2 (EtOAc/Hex 3:7; $KMnO_4$-TLC stain; UV-inactive). ^{1}**H NMR** (500 MHz, Chloroform-d) δ = 6.99 (s, 1H), 4.64 (s, 2H), 2.25 (s, 1H) ppm.

^{13}**C NMR** (126 MHz, CDCl3) δ = 153.47, 147.18, 126.45, 54.99 ppm. **HRMS (ESI)**: calcd. for $C_4H_4Cl_1N_1O_2$ = 132.99251[M + H]$^+$; found mass = 132.992867.

5-(((*tert*-butyldimethylsilyl)oxy)methyl)-2-chlorooxazole (**4**): To a solution of alcohol **3** (0.67 g, 5.03 mmol) in anhydrous DMF (47 mL) was added imidazole (0.86g, 12.59 mmol) and *tert*-butyldimethylsilyl chloride (0.83 g, 5.51 mmol). The reaction mixture was stirred overnight at room temperature under inert atmosphere. The most of solvent was removed in vacuo and the crude was partitioned between brine (150 mL) and EtOAc (40.0 mL). The organic layer was collected, and the aqueous layer was washed with EtOAc (2 × 40 mL). The organic fractions were combined and dried over $MgSO_4$ to be concentrated under reduced pressure. The crude product was purified by flash column chromatography. The appropriate TLC-fractions were combined and concentrated to obtain the desired silyl ether **4** (1.07 g, 86%) as a colourless oil. R_f = 0.67 (EtOAc/ Hex 3:7; $KMnO_4$-TLC stain; UV-inactive). ^{1}**H NMR** (500 MHz, Chloroform-d) δ = 6.91 (s, 1H), 4.63 (s, 2H), 0.91 (s, 9H), 0.11 (s, 6H) ppm. ^{13}**C NMR** (126 MHz, CDCl3) δ = 153.74, 146.60, 125.99, 55.84, 25.77, 18.35, -5.31 ppm. **HRMS (ESI)**: calcd. for $C_{10}H_{18}Cl_1N_1O_2Si_1$ = 247.07898 [M + H]$^+$; found = 247.078435.

5-(((*tert*-butyldimethylsilyl)oxy)methyl)-2-(prop-2-yn-1-yloxy)oxazole (**5**): Sodium hydride [0.17g (60 % dispersion in mineral oil), 4.16 mmol] was placed in a well-dried round-bottom flask with magnetic stirring. The flask was immediately sealed and purged with N_2. After adding anhydrous tetrahydrofuran (7.0 mL), the flask was set up at 0 °C. Propargyl alcohol (0.49 mL, 8.49 mmol) is added dropwise to the sodium hydride suspension stirring the reaction mixture for 1 h at 0 °C under inert atmosphere. After this time, maintaining the same low temperature, a solution of 2-chlorooxazole **4** (0.7g, 2.83 mmol) in anhydrous THF (7.0 mL) was added slowly. The reaction mixture was stirred at room temperature under inert atmosphere for 36 h. Then, the flask is placed in an ice bath and the reaction mixture was quenched with NH_4Cl solution (80.0 mL). Using a separatory funnel, the aqueous solution is washed with EtOAc (3×40 mL). The organic fractions were combined and dried over $MgSO_4$. The drying agent is filtered off and the solvent was evaporated in vacuo. Desired *O*-propargyl derivative **5** (colourless oil, 0.68 g, 90%) was isolated from silica gel column chromatography. R_f = 0.33 (EtOAc/ Hex 1:9; $KMnO_4$-TLC stain; UV-inactive). ^{1}**H NMR** (500 MHz, Chloroform-d) δ = 6.65 (s, 1H), 4.96 (d, *J* = 2.4 Hz, 2H), 4.54 (s, 2H), 2.58 (t, *J* = 2.4 Hz, 1H), 0.89 (s, 9H), 0.09 (s, 6H) ppm. ^{13}**C NMR** (126 MHz, CDCl3) δ = 160.91, 146.65, 123.47, 76.74, 76.33, 58.34, 55.86, 25.83, 18.38, -5.26 ppm. **HRMS (ESI)**: calcd. for $C_{13}H_{22}N_1O_3Si_1$ = 268.13635[M + H]$^+$; found = 268.1364.

(2-(prop-2-yn-1-yloxy)oxazol-5-yl)methanol (**6**): Tetrabutylammonium fluoride solution [4.65 mL (1.0 M in THF), 4.65mmol) is added dropwise to a cold solution (0 °C) of silyl ether derivative **5** (0.83 g, 3.1 mmol) in tetrahydrofuran (12.0 mL). Continuous stirring for 60 min at room temperature was found to be a suitable setup for an efficient *O*-silyl cleavage reaction. After this time, the mixture reaction was poured into brine (100 mL). Using a separatory funnel, the aqueous solution is washed with EtOAc (3×40 mL). The organic fractions were combined and dried over $MgSO_4$. After filtering off the drying agent, the solvent is removed under reduced pressure. The silica gel column chromatography yielded 0.42 g (89 %) of desired benzyl alcohol **6** as a slightly yellow oil. R_f = 0.5 (EtOAc/ Hex 7:3; $KMnO_4$-TLC stain; UV-inactive). ^{1}**H NMR** (500 MHz, Chloroform-d) δ = 6.68 (s, 1H), 4.94 (d, *J* = 2.4 Hz, 2H), 4.50 (s, 2H), 2.99 (s, 1H), 2.59 (t, *J* = 2.4 Hz, 1H) ppm. ^{13}**C NMR** (126 MHz, CDCl3) δ = 161.11, 146.58, 123.94, 76.60, 76.53, 58.50, 55.07 ppm. **HRMS (ESI)**: calcd. for $C_7H_7N_1O_3$ = 153.04204 [M + H]$^+$; found = 153.042050.

4-Nitrophenyl ((2-(prop-2-yn-1-yloxy)oxazol-5-yl)methyl) carbonate (**7**): In a well-dried round-bottom flask with magnetic stirring, was placed benzyl alcohol derivative 6 (0.33 g, 2.15 mmol). The flask was immediately sealed and purged with N_2. Then, anhydrous tetrahydrofuran (7.0 mL) and anhydrous pyridine (0.26 mL, 3.23 mmol) was added into the flask followed by a solution of 4-nitrophenyl chloroformate (0.65 g, 3.23 mmol) in anhydrous tetrahydrofuran (7.0 mL). The reaction mixture was stirred at room temperature under inert atmosphere for 12 h. After this time, TLC indicated the disappearance of the starting material. In order to remove the pyridine, the mixture reaction was poured into a separatory funnel containing a solution of citric acid (4.0 g/100 mL water). Such aqueous solution was washed with EtOAc (3×40 mL). The organic fractions were combined and dried over $MgSO_4$. The drying agent is filtered off and the solvent was evaporated in vacuo. The residue was purified by silica gel column chromatography to furnish the corresponding carbonate **8** (0.41 g, 60%) showing being a thick oil which turns as a white solid in cold storage. R_f = 0.35 (EtOAc/ Hex 3:7; UV-active). ^{1}**H NMR** (500 MHz, Chloroform-d) δ = 8.31–8.25 (m, 2H), 7.42–7.35 (m, 2H), 6.97 (s, 1H), 5.18 (s, 2H), 5.01 (d, *J* = 2.4 Hz, 2H), 2.62 (t, *J* = 2.4 Hz, 1H) ppm. ^{13}**C NMR** (126 MHz, CDCl3) δ = 161.84, 155.33,

152.17, 145.56, 140.73, 128.44, 125.36, 121.76, 76.73, 76.38, 60.10, 58.78 ppm. **HRMS (ESI)**: calcd. for $C_{14}H_{11}N_2O_7$ = 319.05608 [M + H]$^+$; found = 319.0554. Calcd. for $C_{14}H_{10}N_2O_7Na_1$ = 341.03802 [M + Na]$^+$; found = 341.0381.

POxOC-DOX (**9b**): In a well-dried round-bottom flask with magnetic stirring, was placed doxorubicin hydrochloride (45 mg, 0.083 mmol). The flask was promptly sealed and purged with N_2. Then, anhydrous dioxane/MeCN 1:1 (3.0 mL) and anhydrous triethylamine (0.057 mL, 0.415 mmol) was added into the flask followed by a solution of carbonate **8** (0.031g, 0.099 mmol) in anhydrous dioxane/MeCN 1:1 (1.0 mL) (added dropwise). The reaction mixture was stirred at room temperature under inert atmosphere for 2 days. After this time, TLC indicated the disappearance of doxorubicin. The solvent is evaporated under reduced pressure. The crude was purified by column chromatography eluting with DCM/MeOH 9:1 to give 0.029 g, (49%) of a red solid corresponding to desired prodrug **9b**. R_f = 0.42 (DCM/MeOH 9:1). **^{1}H NMR** (500 MHz, DMSO-d6) δ = 14.00 (s, 1H), 13.24 (s, 1H), 7.94 – 7.83 (m, 2H), 7.62 (m, 1H), 6.97 – 6.90 (m, 2H), 5.43 (s, 1H), 5.21 (m, 1H), 5.02 (d, *J* = 2.4 Hz, 2H), 4.92 (m, 1H), 4.87 (s, 2H), 4.84 (t, *J* = 5.9 Hz, 1H), 4.69 (d, *J* = 5.7 Hz, 1H), 4.58 (d, *J* = 5.9 Hz, 2H), 4.15 (p, *J* = 7.5, 6.9 Hz, 1H), 3.98 (s, 3H), 3.76 – 3.70 (m, 1H), 3.69 (t, *J* = 2.5 Hz, 1H), 3.50 – 3.40 (m, 1H), 3.03 – 2.86 (m, 2H), 2.27 – 2.06 (m, 2H), 1.84 (td, *J* = 12.7, 4.1 Hz, 1H), 1.47 (dd, *J* = 12.7, 4.3 Hz, 1H), 1.24 (s, 2H), 1.13 (d, *J* = 6.7 Hz, 3H) ppm. **^{13}C NMR** (126 MHz, DMSO) δ = 213.66, 186.38, 186.27, 160.67, 160.47, 155.99, 154.60, 154.39, 142.99, 136.07, 135.40, 134.52, 133.97, 126.17, 119.87, 119.60, 118.87, 110.64, 110.51, 100.14, 79.15, 77.38, 74.84, 69.72, 67.77, 66.52, 63.58, 58.57, 56.47, 55.05, 47.13, 36.48, 31.97, 29.63, 16.90 ppm. **HRMS (ESI)**: calcd. for $C_{35}H_{35}N_2O_{15}$ = 723.20319 [M + H]$^+$; found = 723.2033. Calcd. for $C_{35}H_{34}N_2O_{15}Na_1$ = 745.18514 [M + Na]$^+$; found = 745.1852.

NMR spectra of prodye 7b

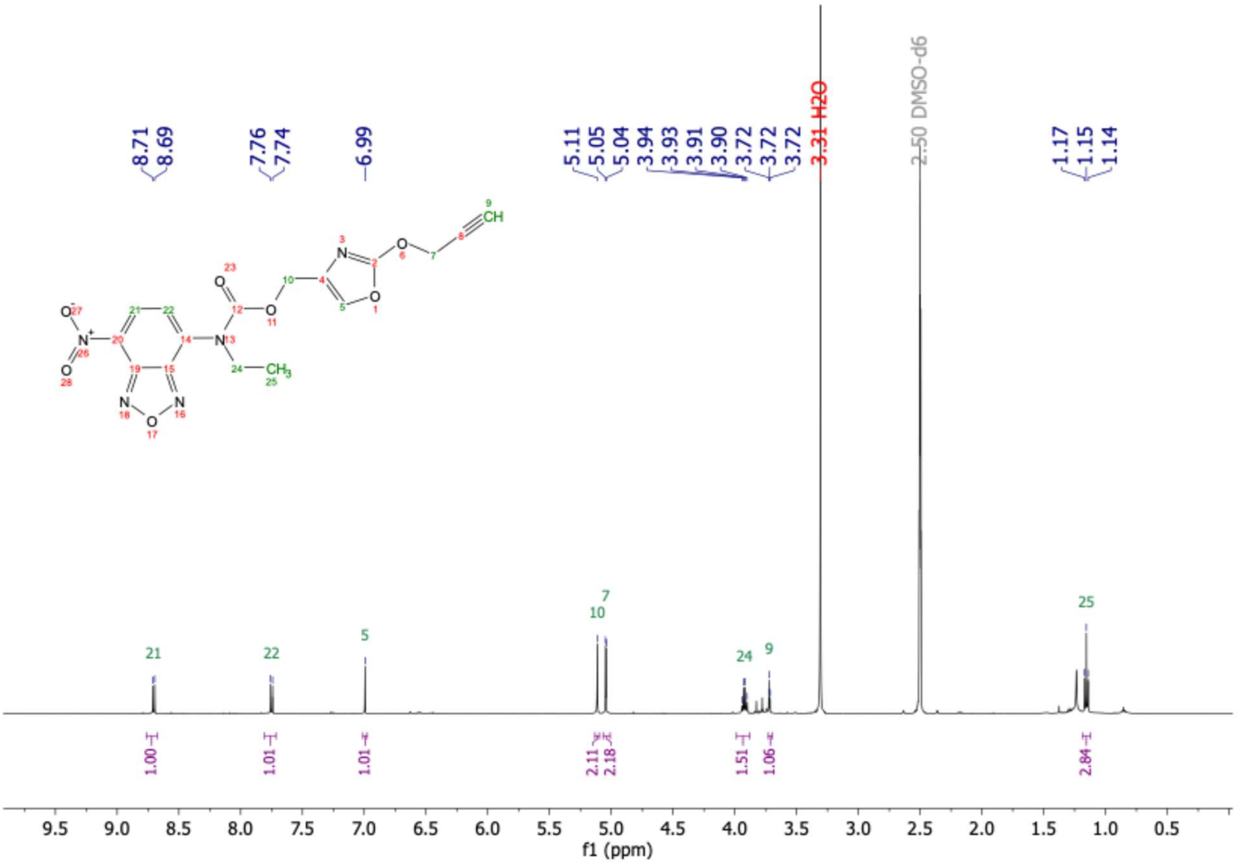

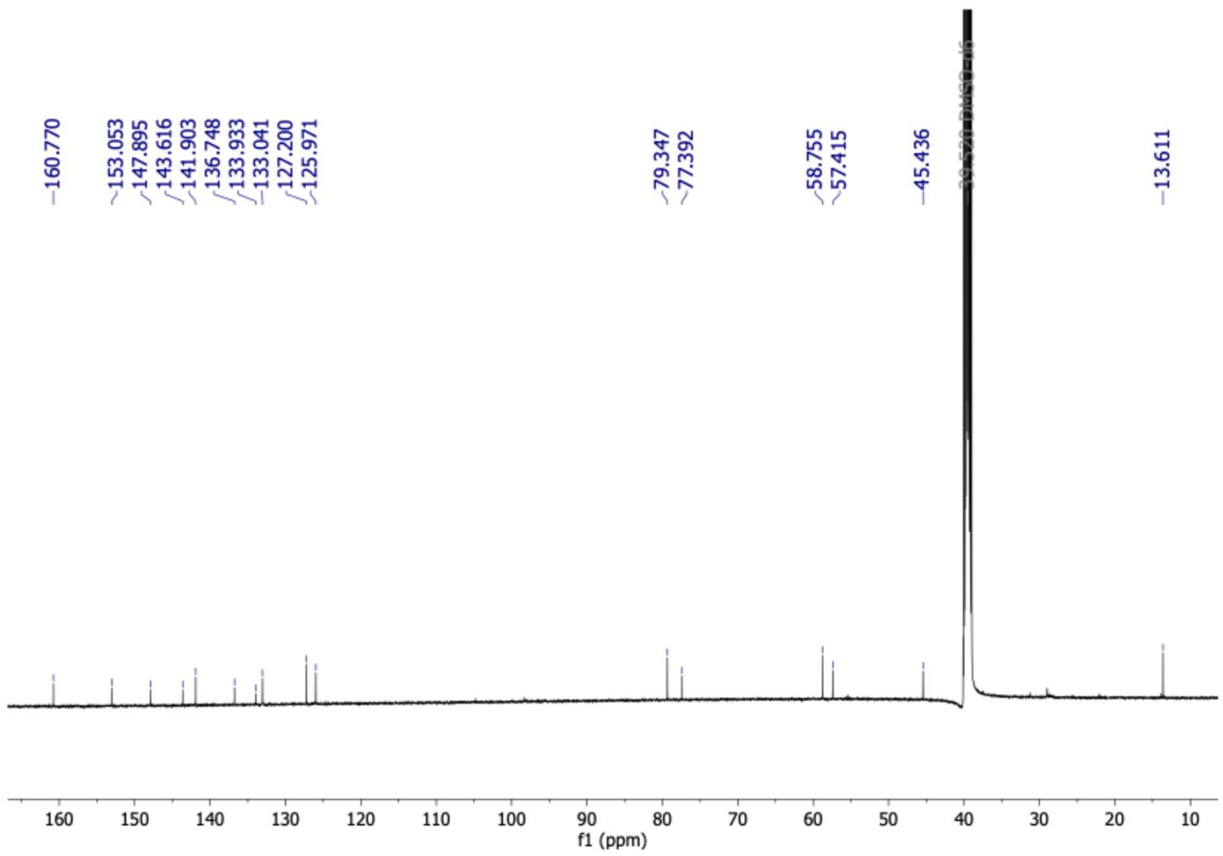

NMR spectra of prodye 7c

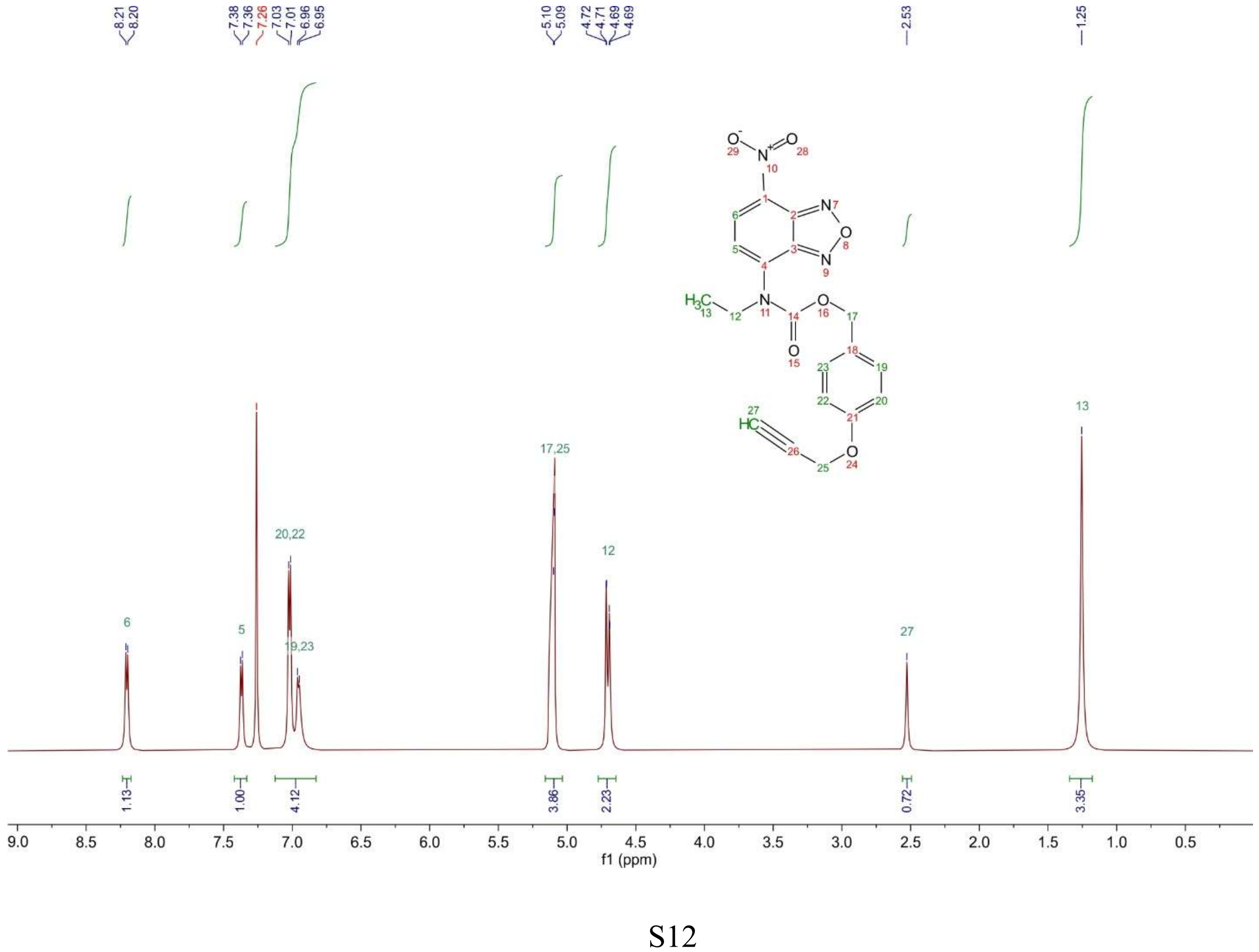

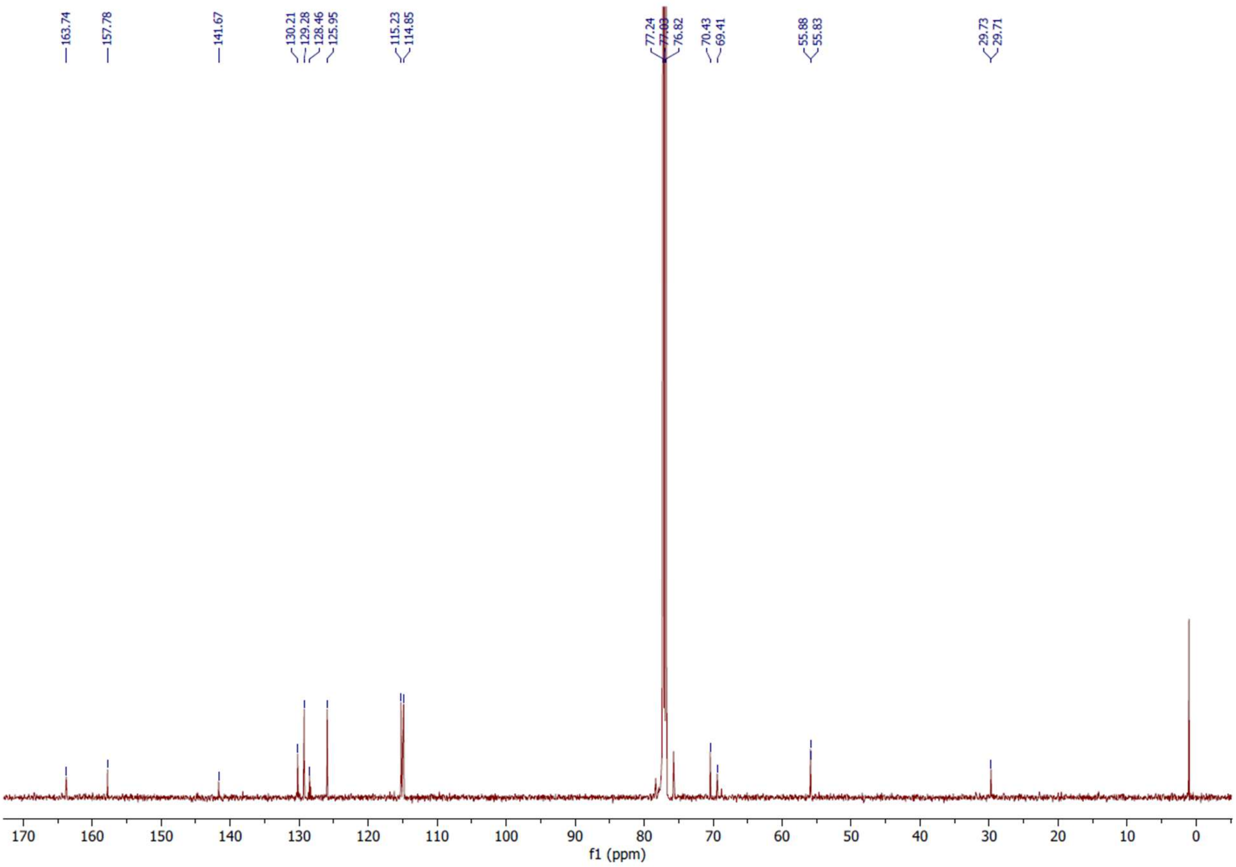
163.74
157.78
141.67
130.21
129.28
128.46
125.95
115.23
114.85
77.24
77.03
76.82
70.43
69.41
55.88
55.83
29.73
29.71
170 160 150 140 130 120 110 100 90 80 70 60 50 40 30 20 10 0
f1 (ppm)

NMR spectra of compound 7d

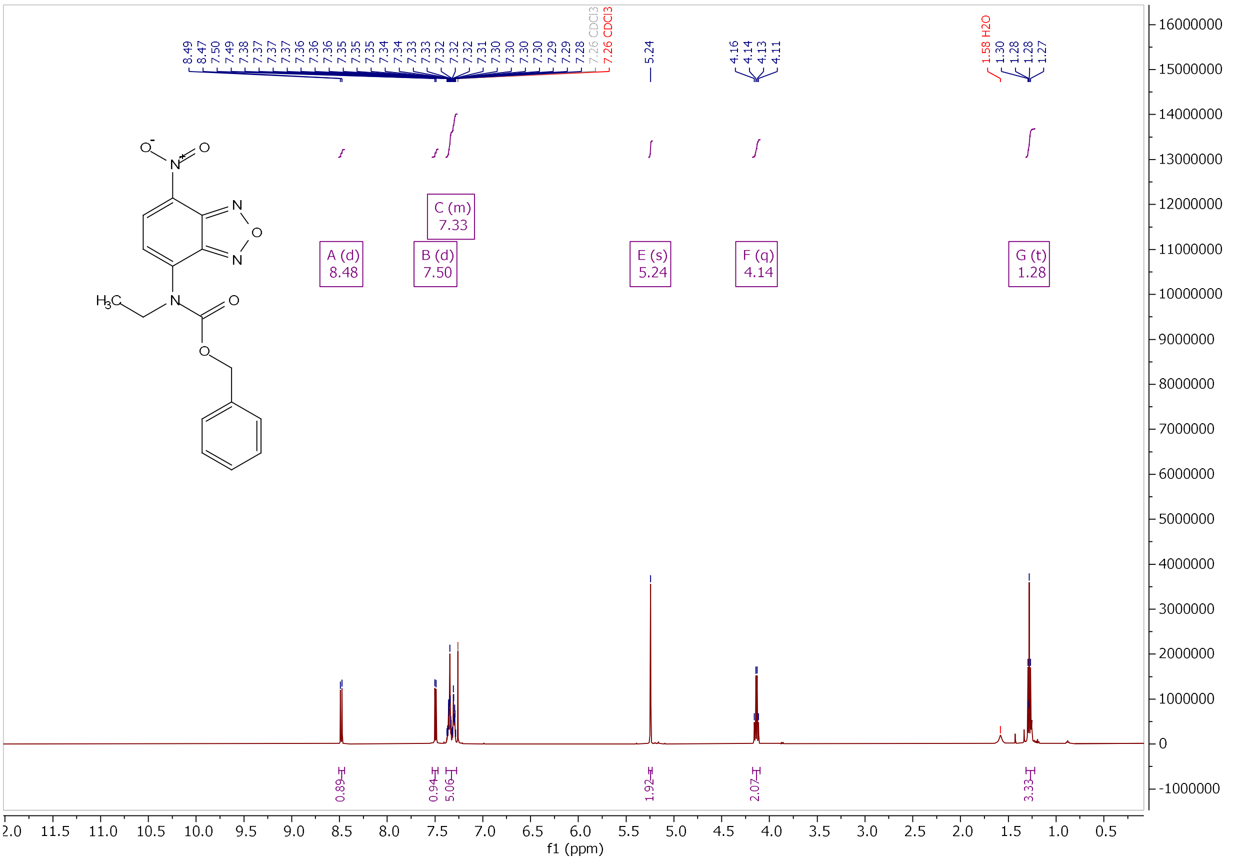

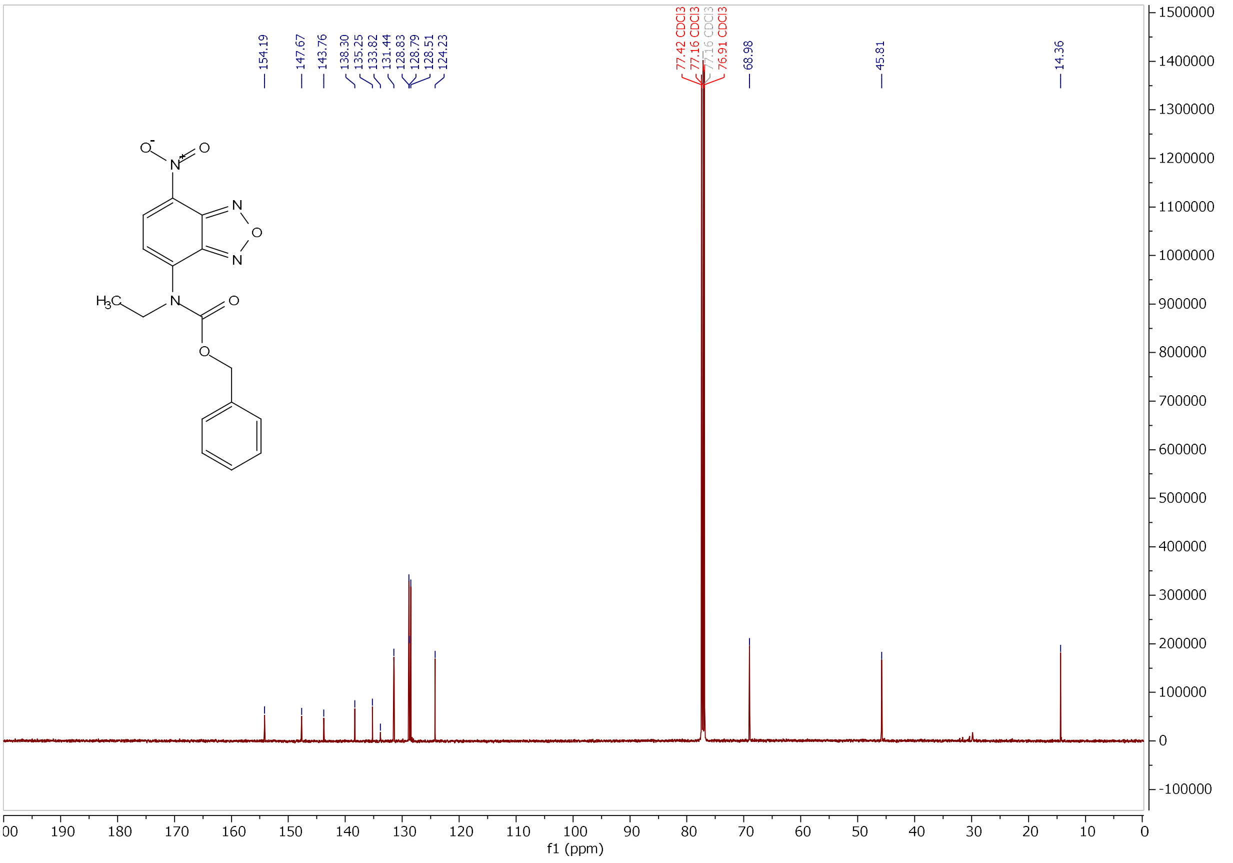

NMR Spectra of compound 3 (*see* Scheme S2)

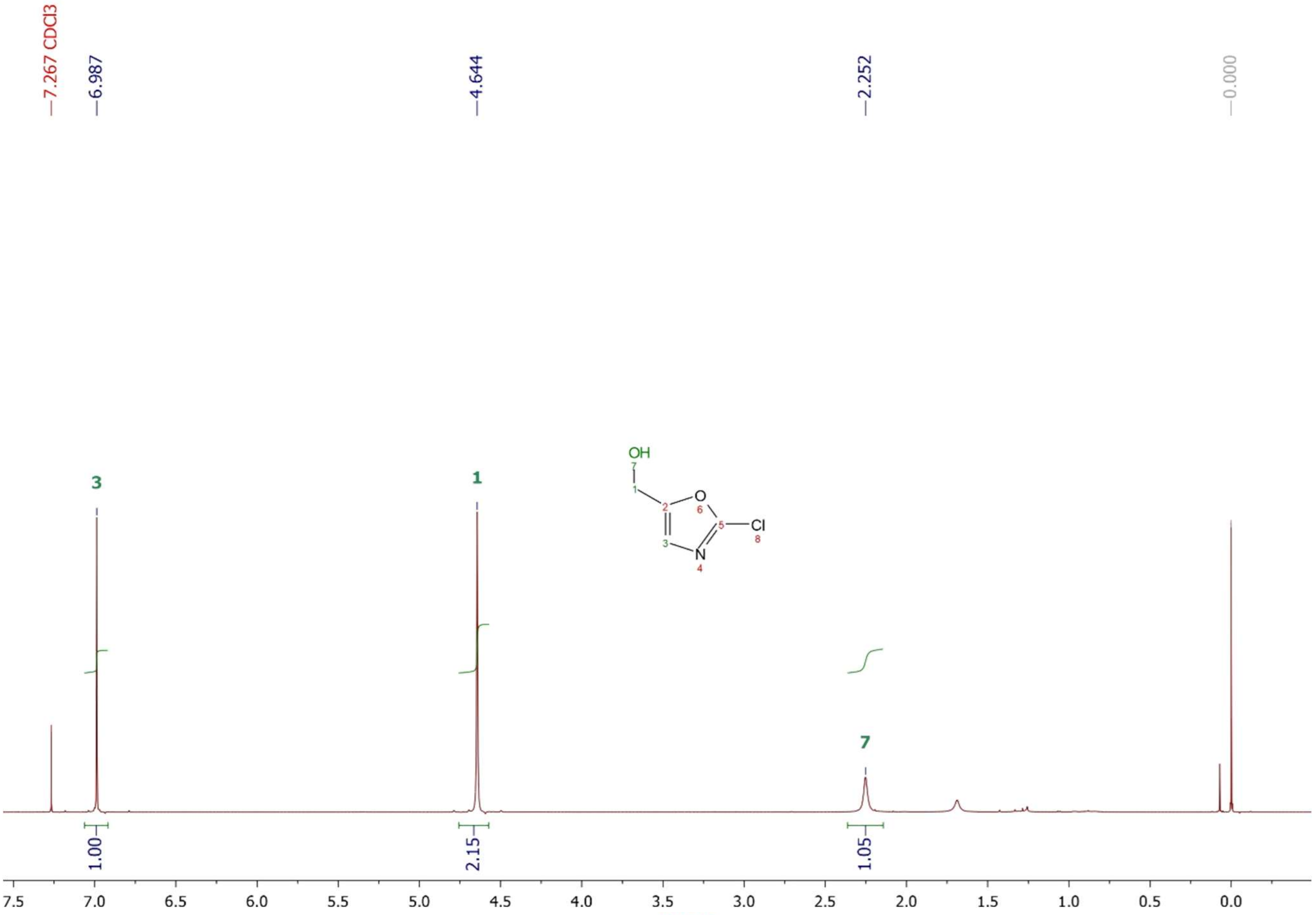

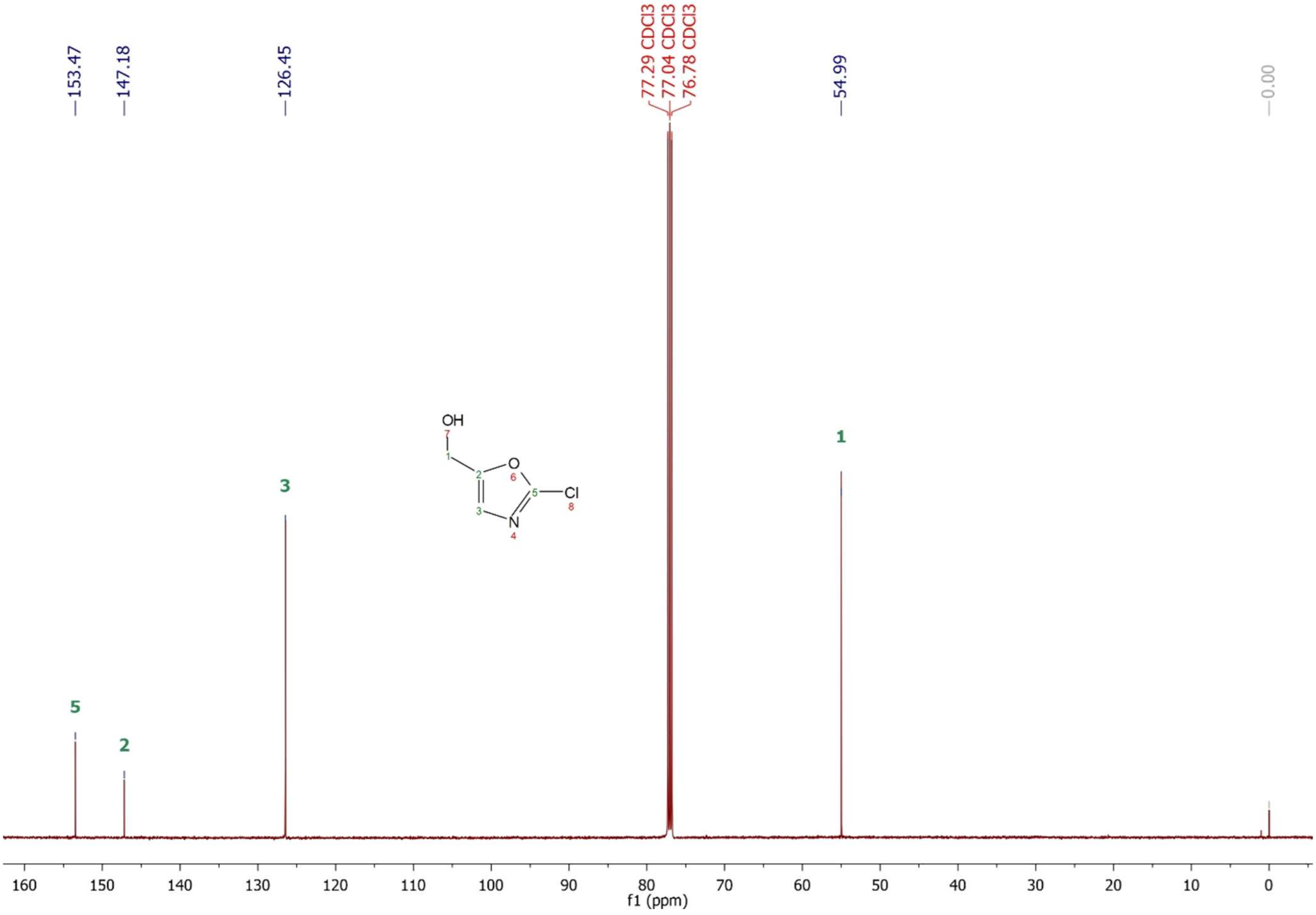

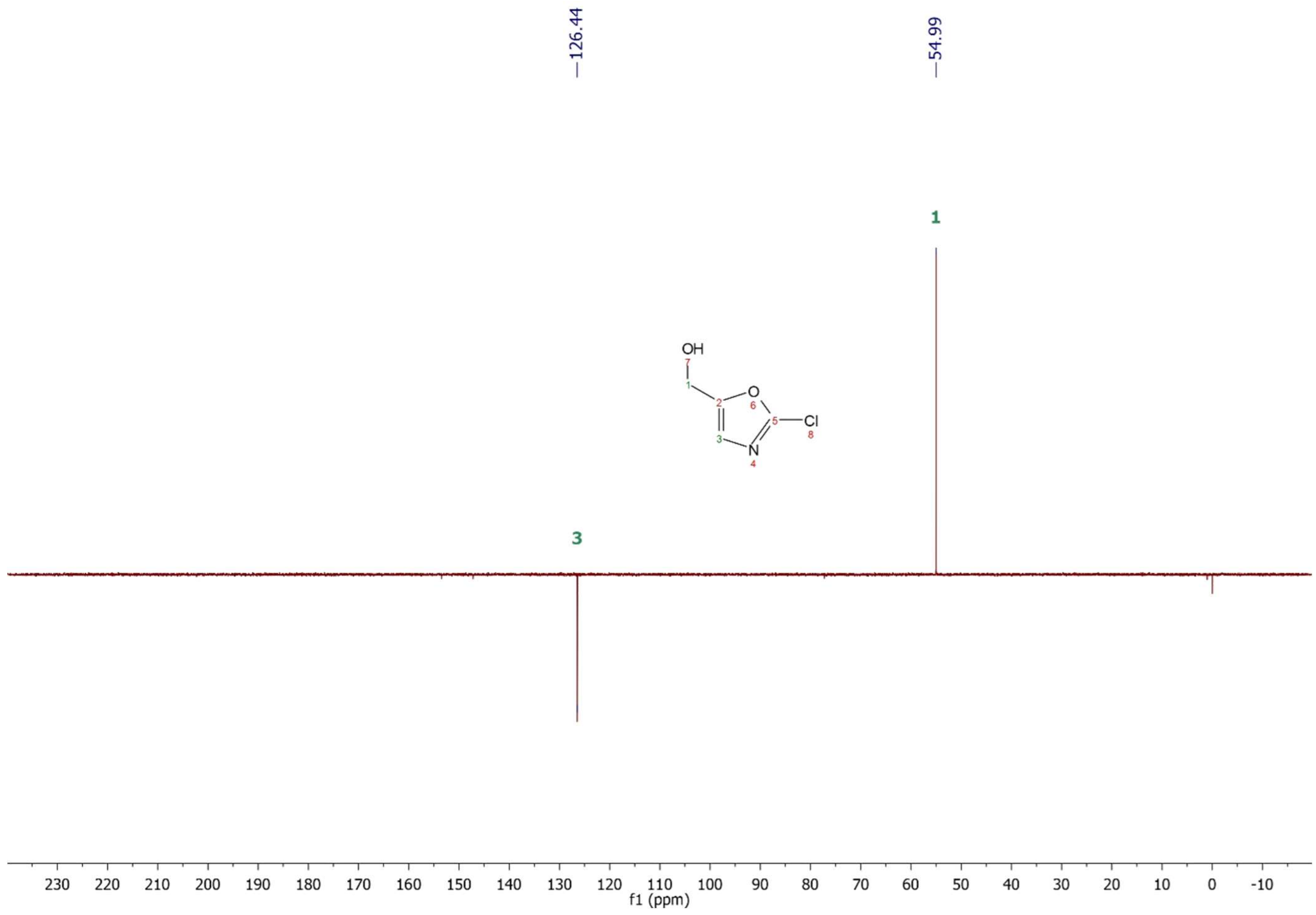

—126.44
—54.99
1
OH
7
1
O
2
6
5
Cl
8
3
N
4
3
230
220
210
200
190
180
170
160
150
140
130
120
110
100
90
80
70
60
50
40
30
20
10
0
-10
f1 (ppm)

NMR Spectra of compound 4 (*see* Scheme S2)

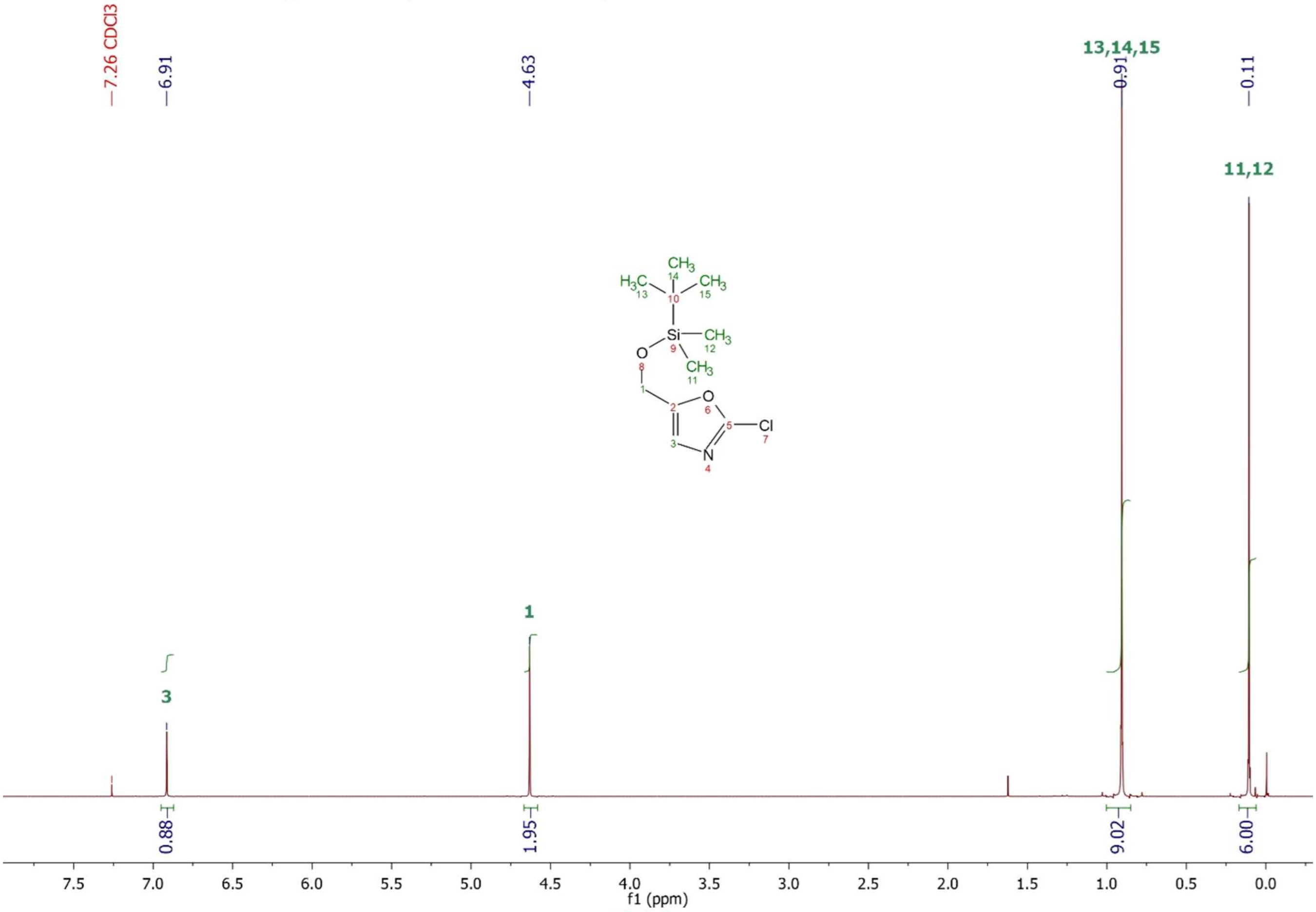

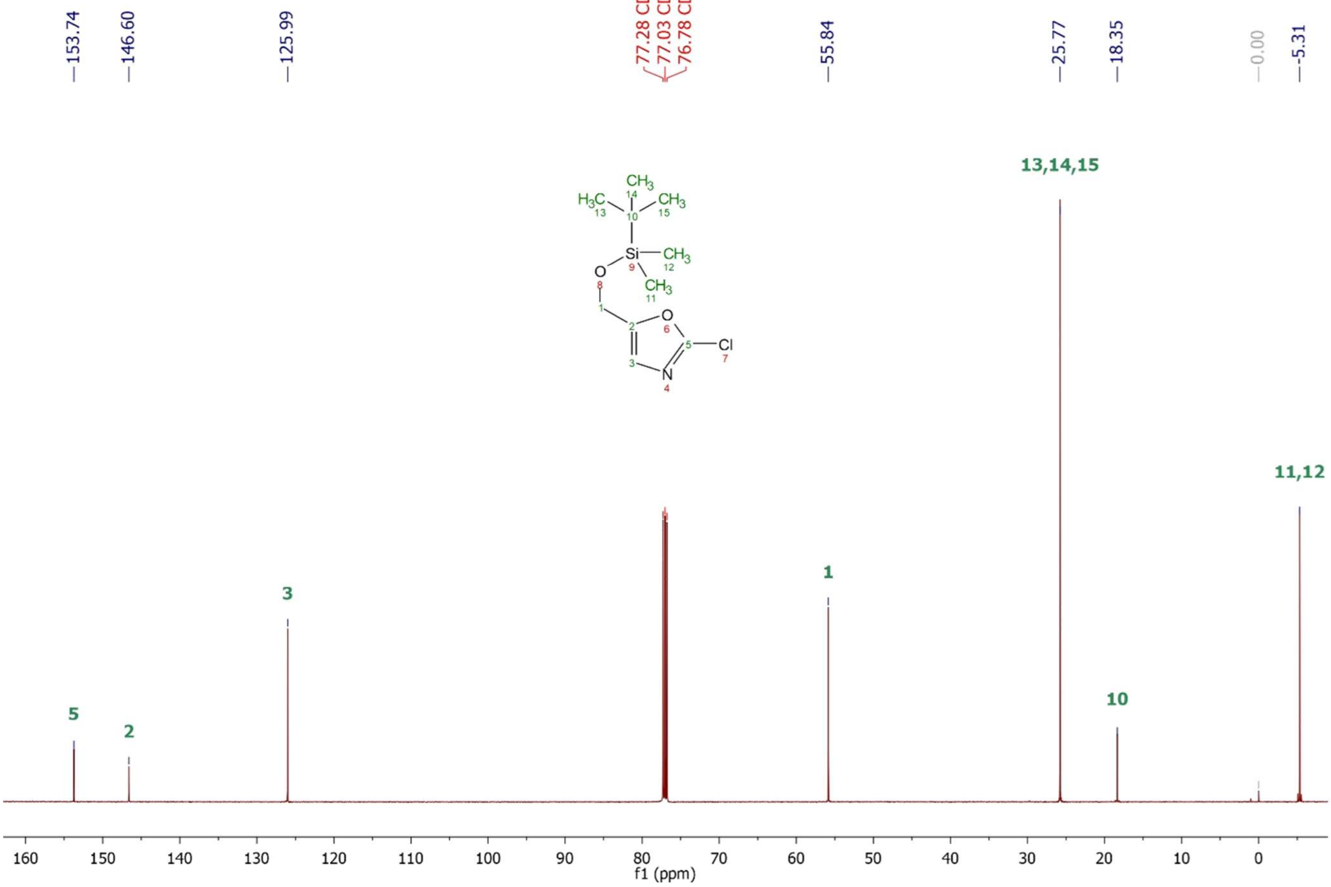

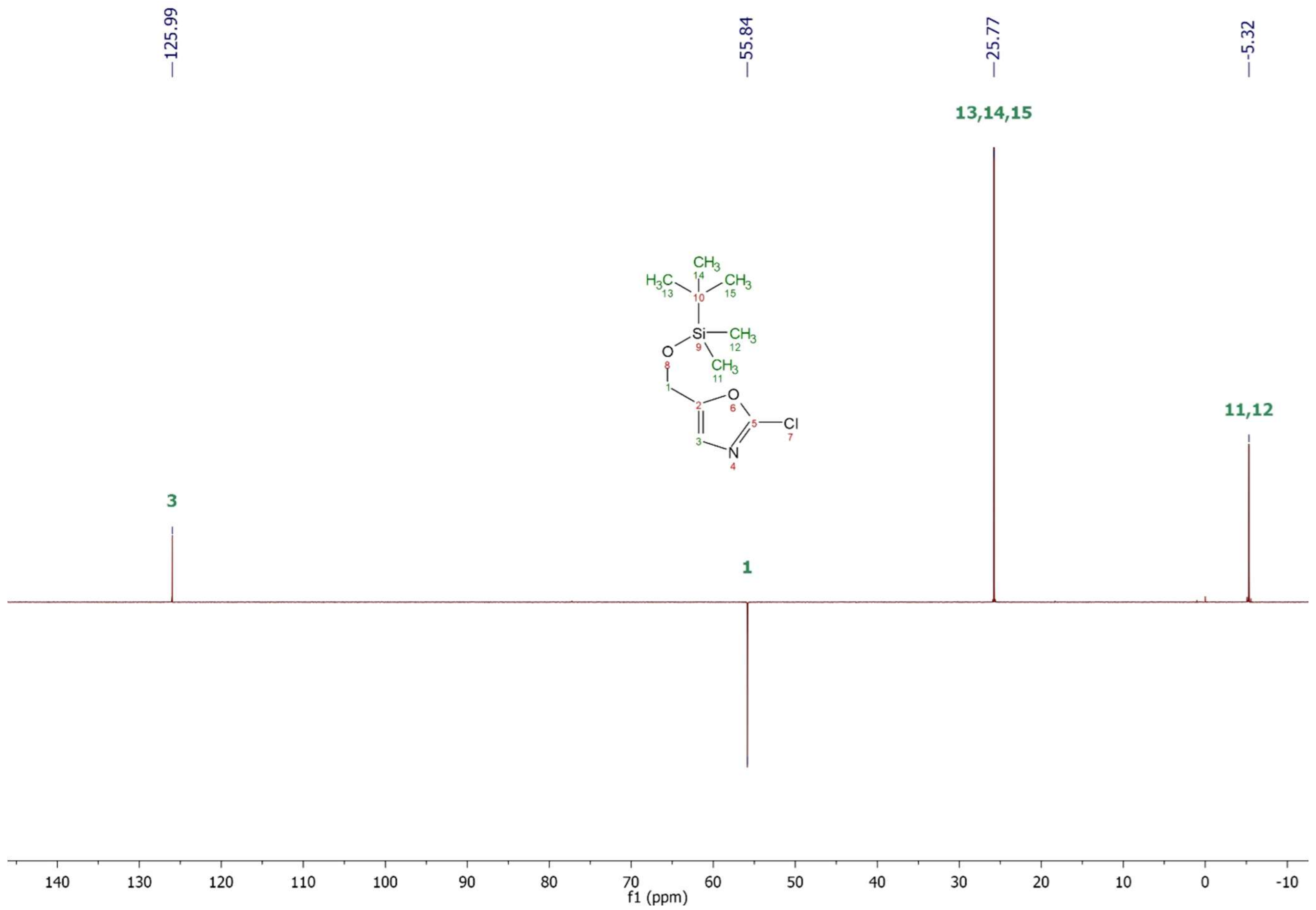

—125.99
—55.84
—25.77
—-5.32
13,14,15
11,12
3
1
140
130
120
110
100
90
80
70
60
50
40
30
20
10
0
-10
f1 (ppm)

NMR Spectra of compound 5 (*see* Scheme S2)

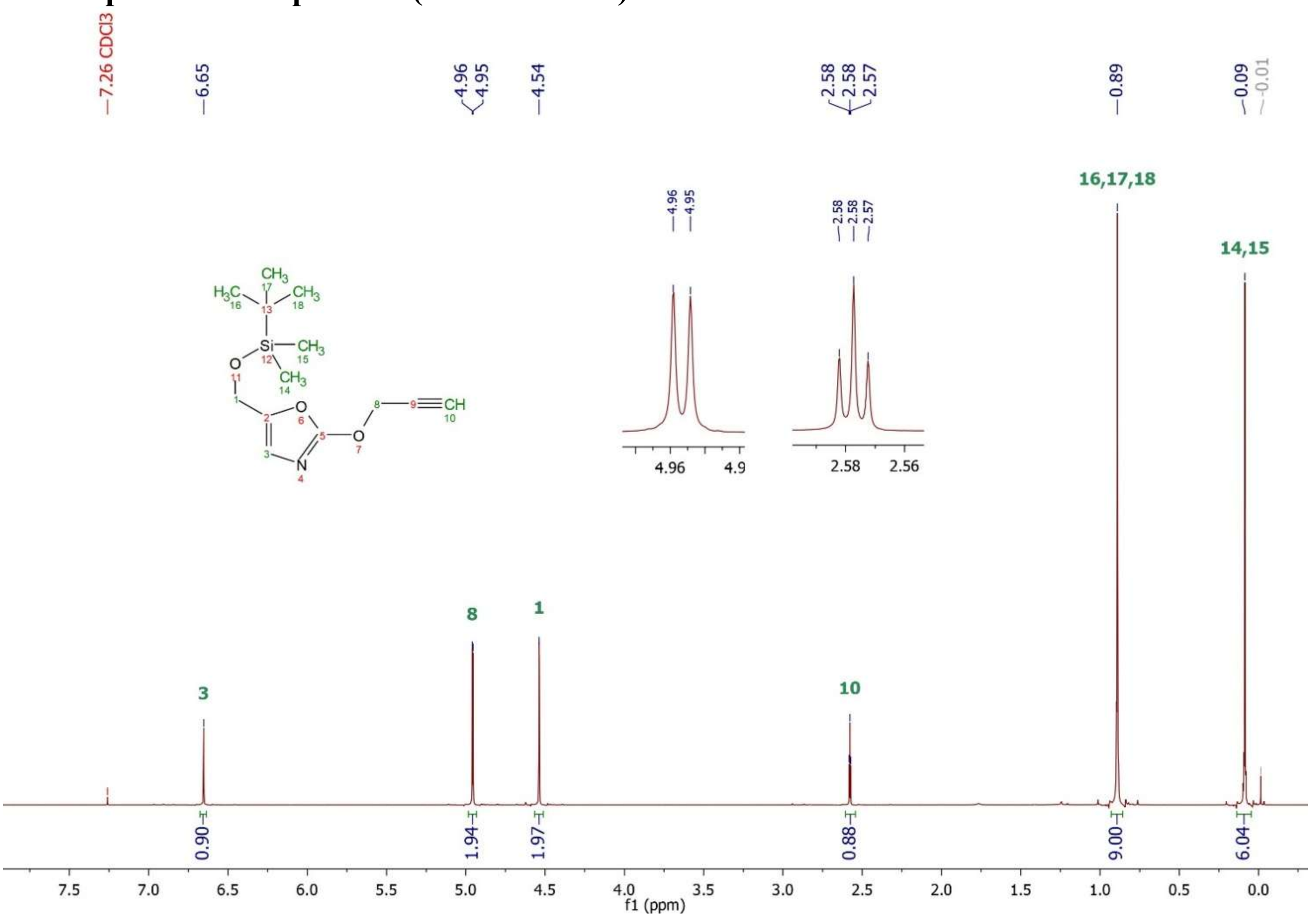

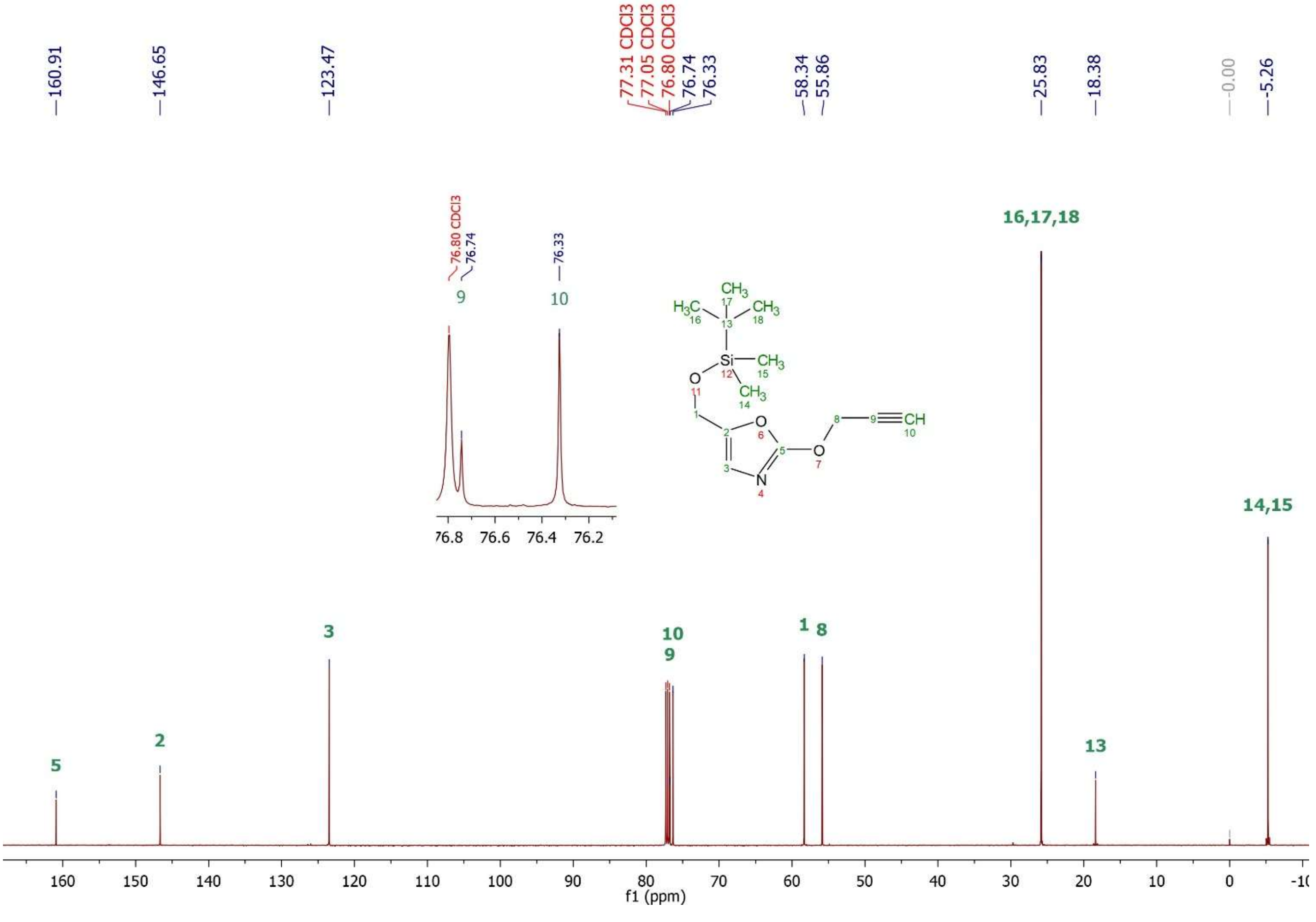

NMR Spectra of compound 6 (*see* Scheme S2)

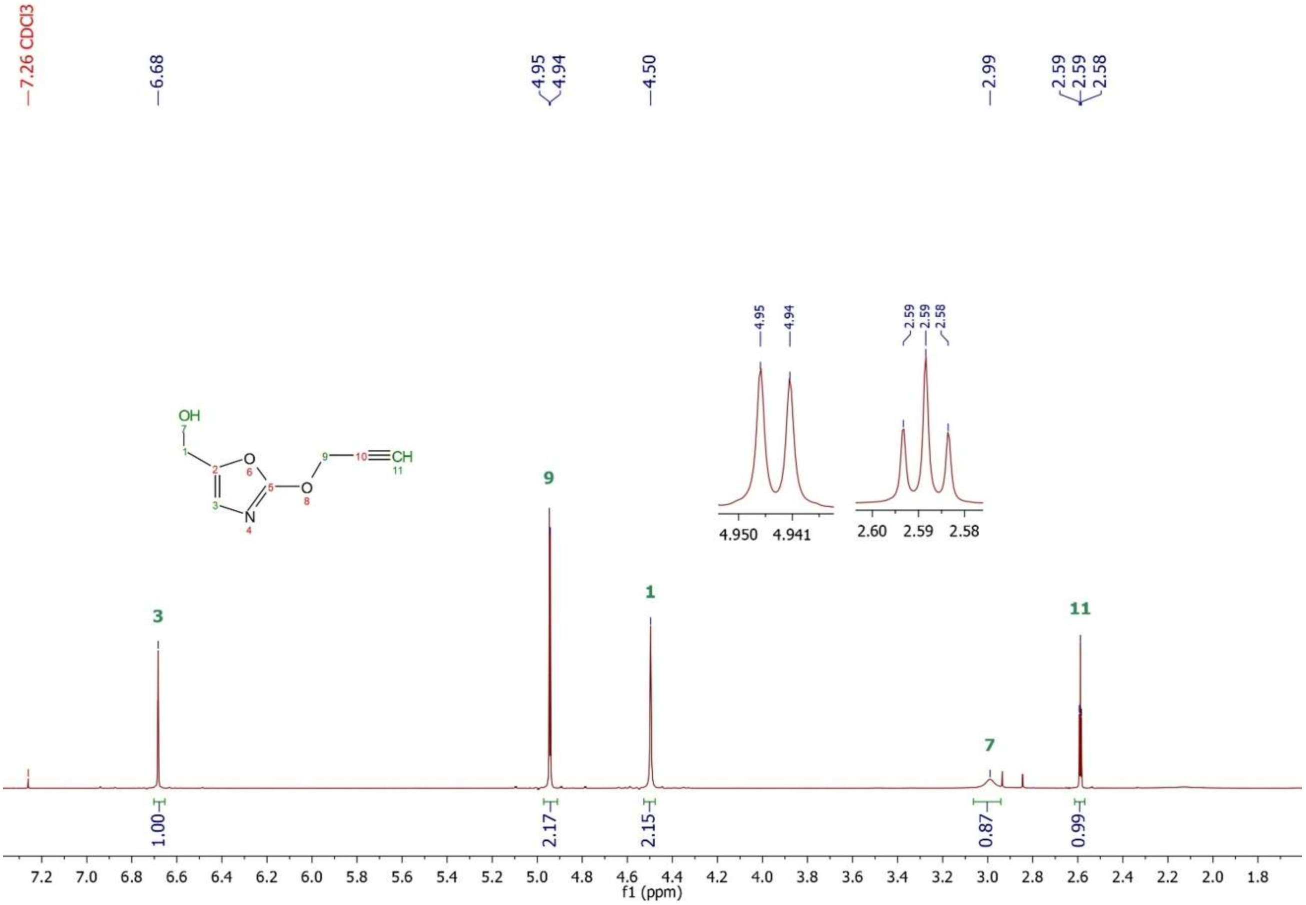

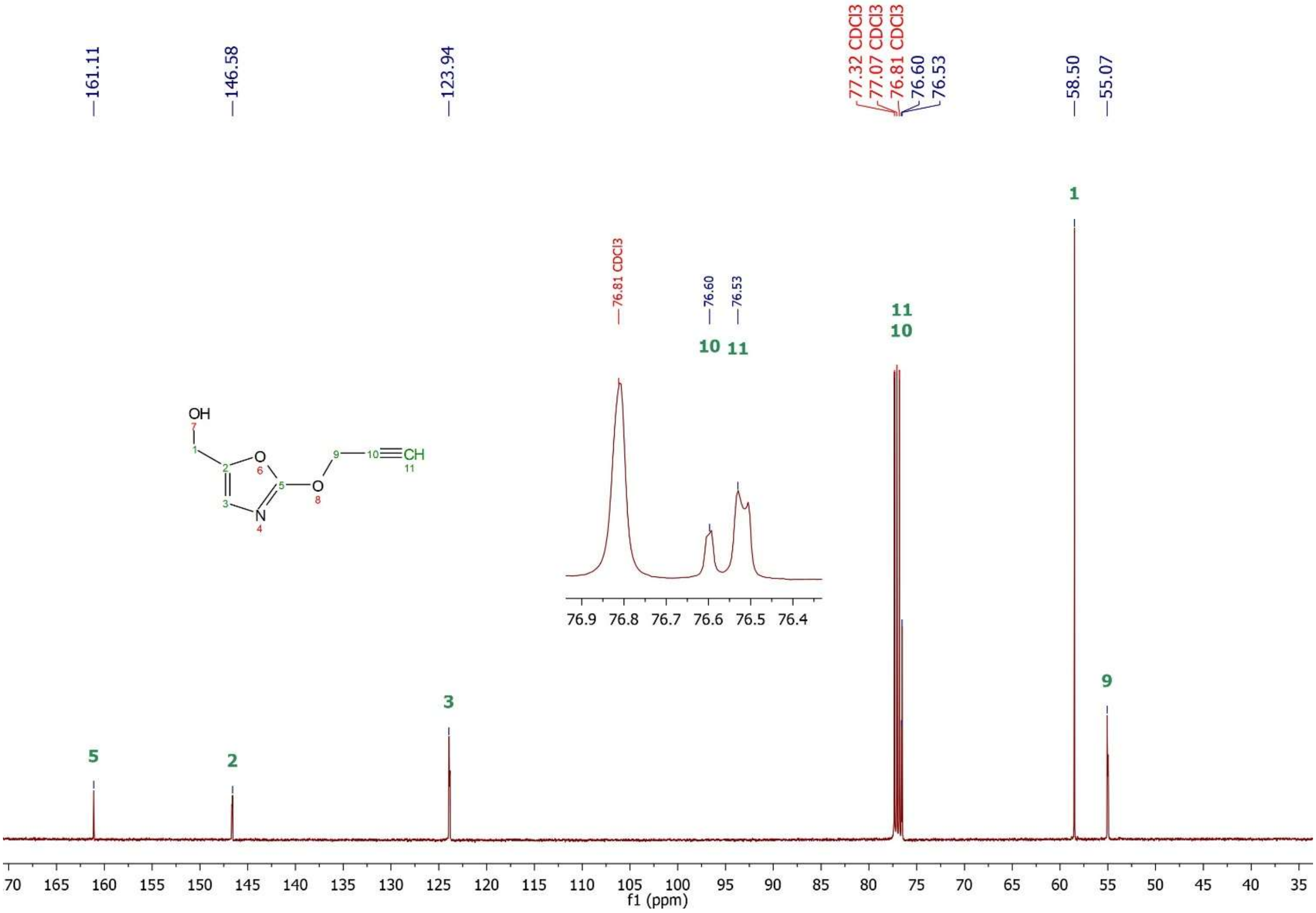

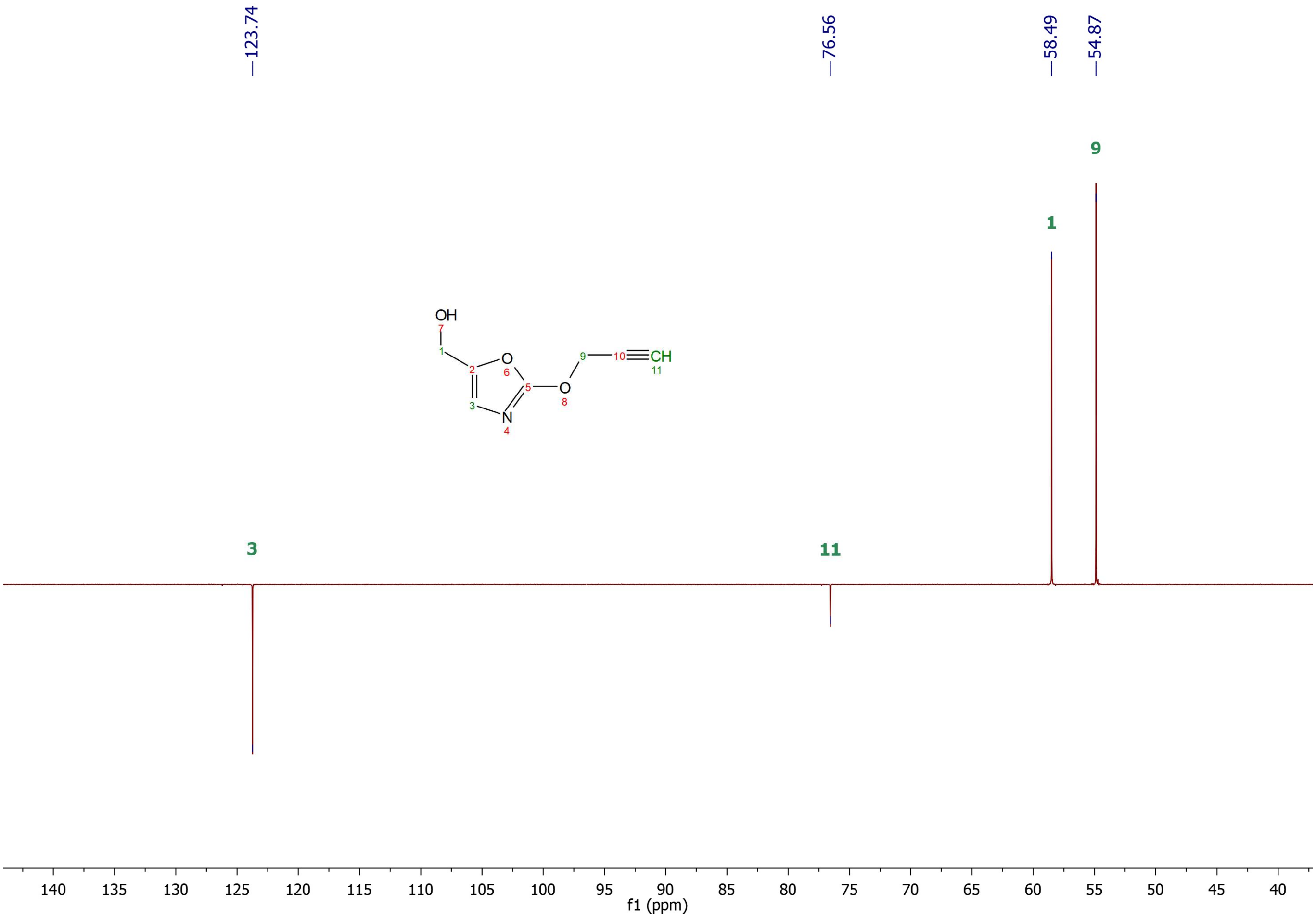

123.74
76.56
58.49
54.87
9
1
3
11
OH
7
1
2
O
6
5
3
N
4
O
8
9
10
CH
11
140 135 130 125 120 115 110 105 100 95 90 85 80 75 70 65 60 55 50 45 40
f1 (ppm)

NMR Spectra of compound (*see* Scheme S2)

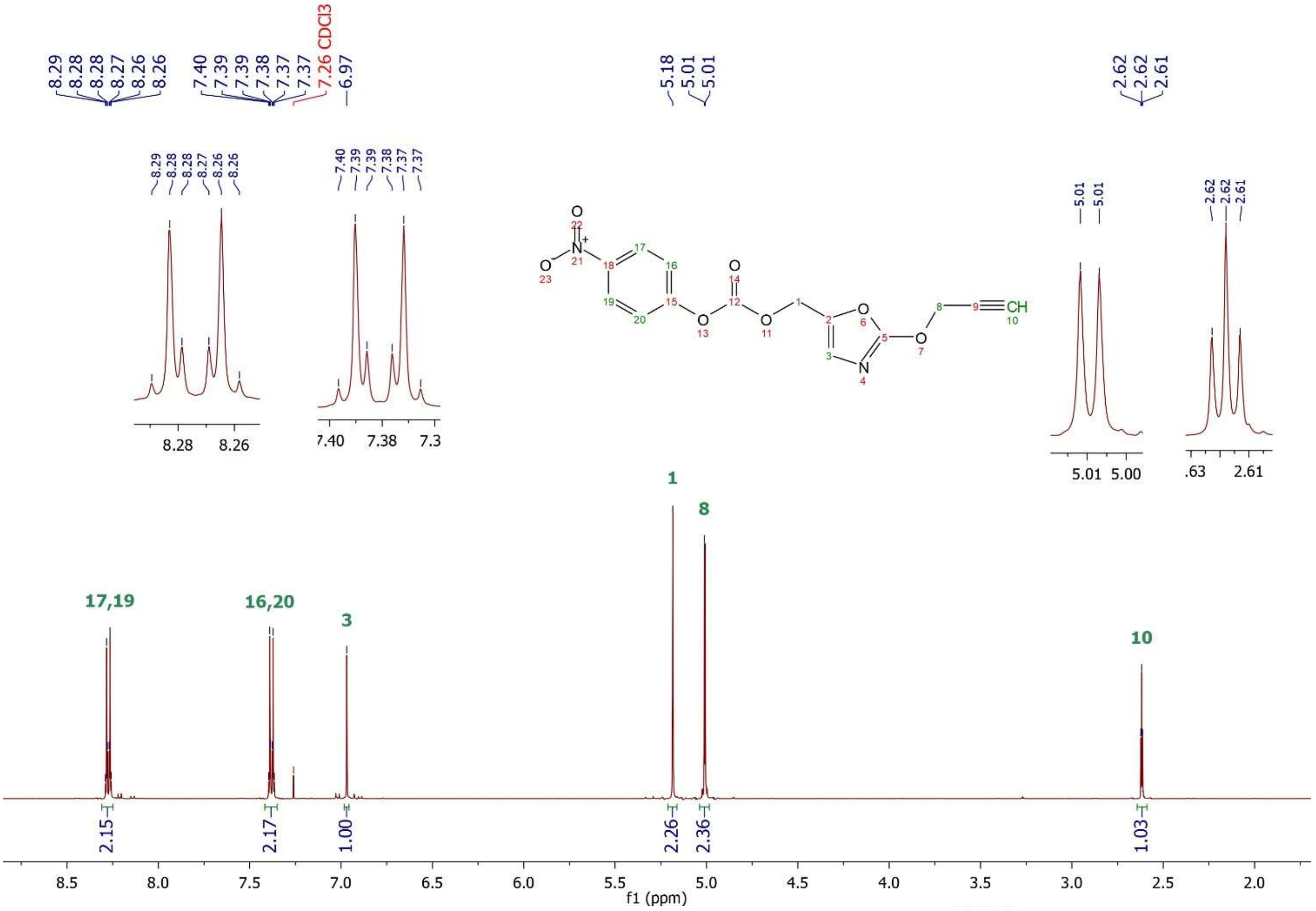

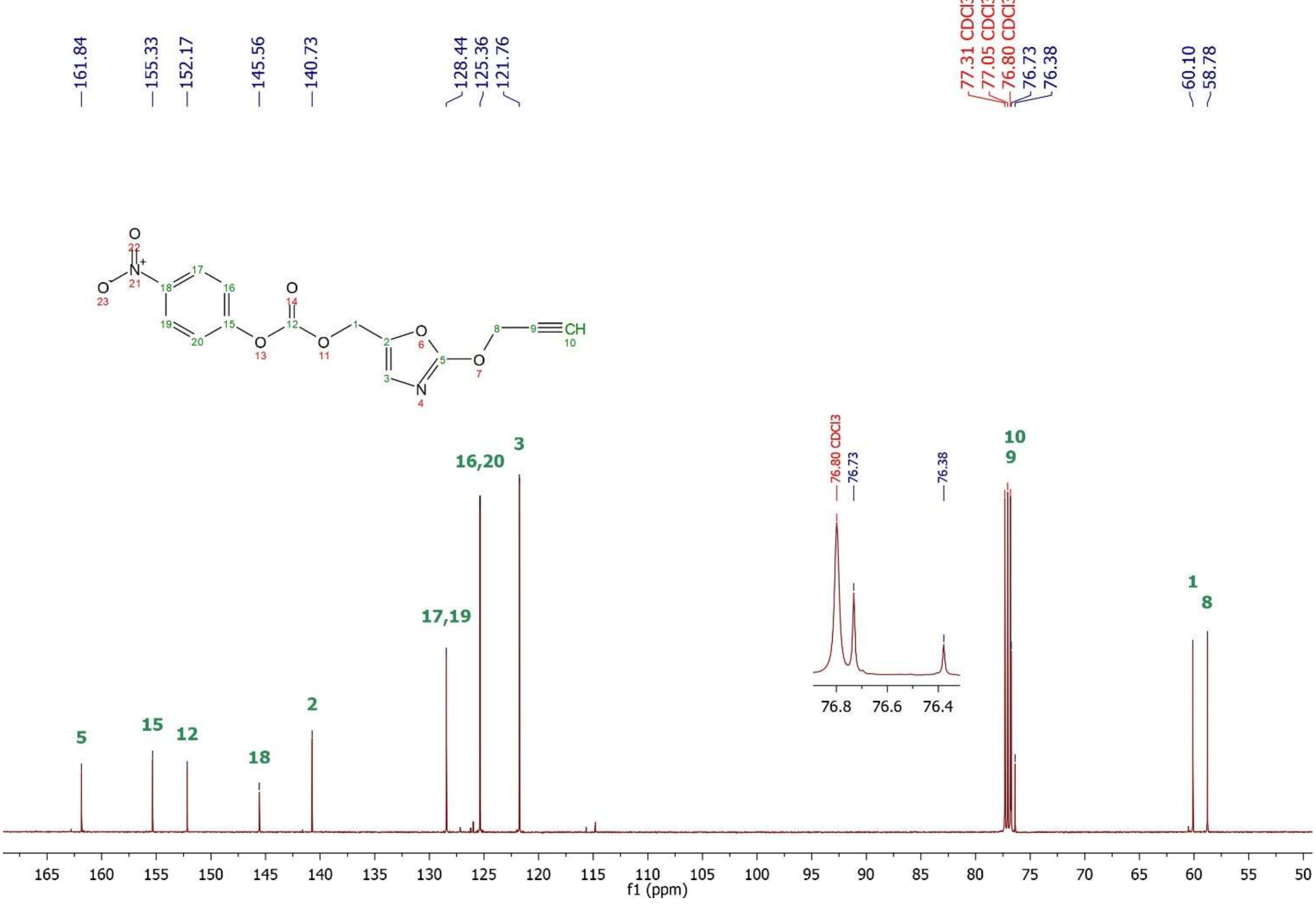

NMR Spectra of prodrug 9b POxOC-DOX (*see* Scheme S2)

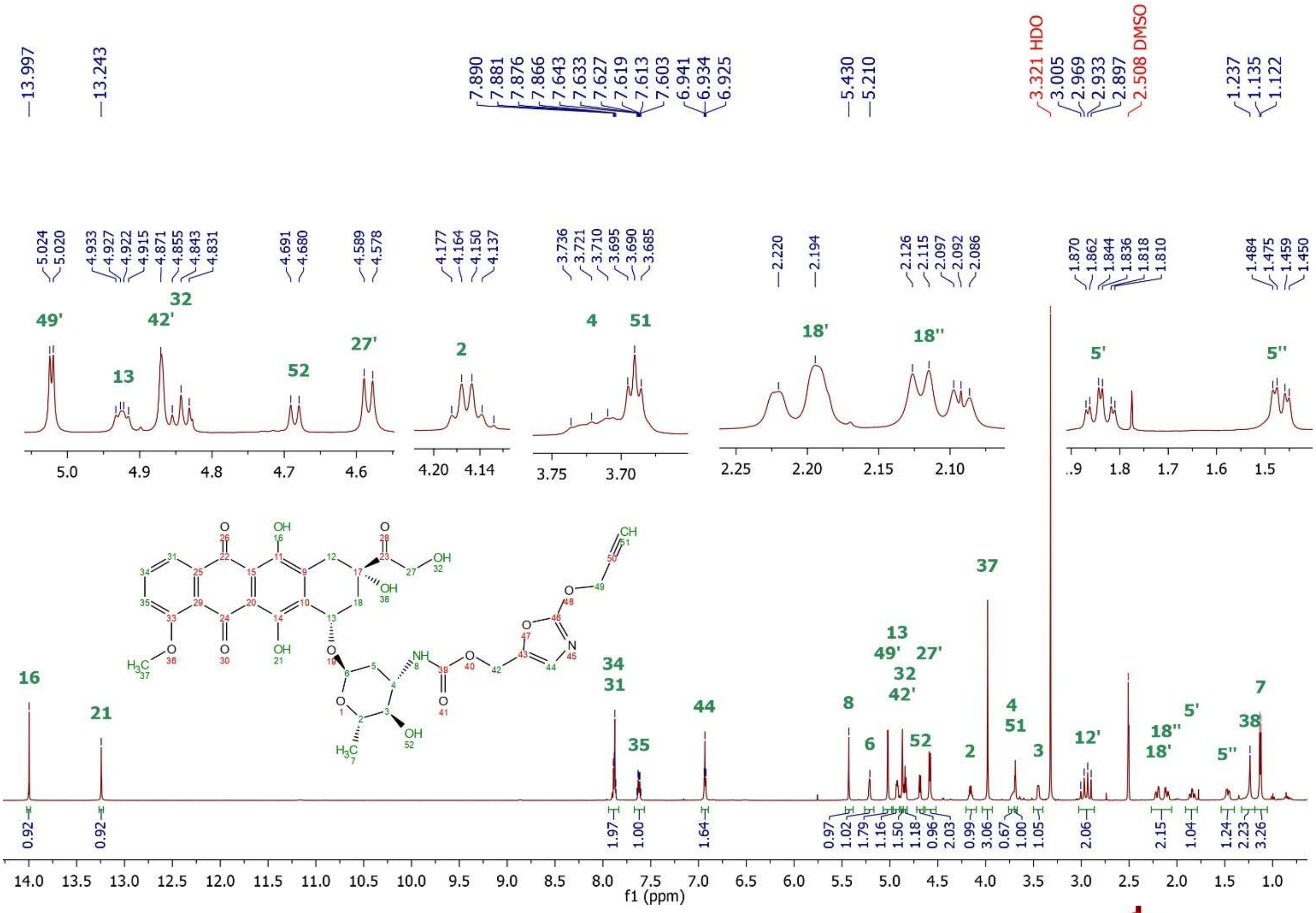

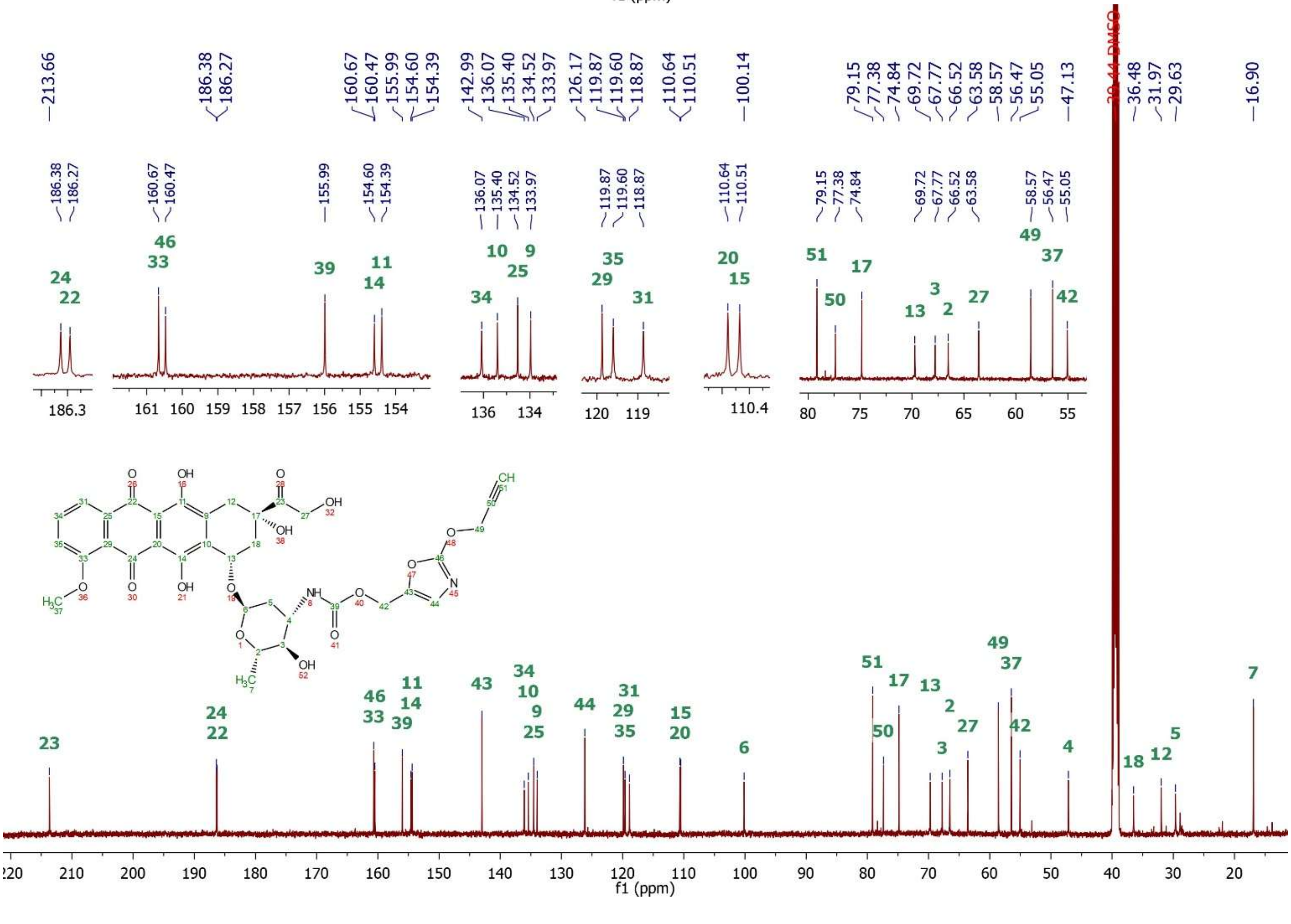

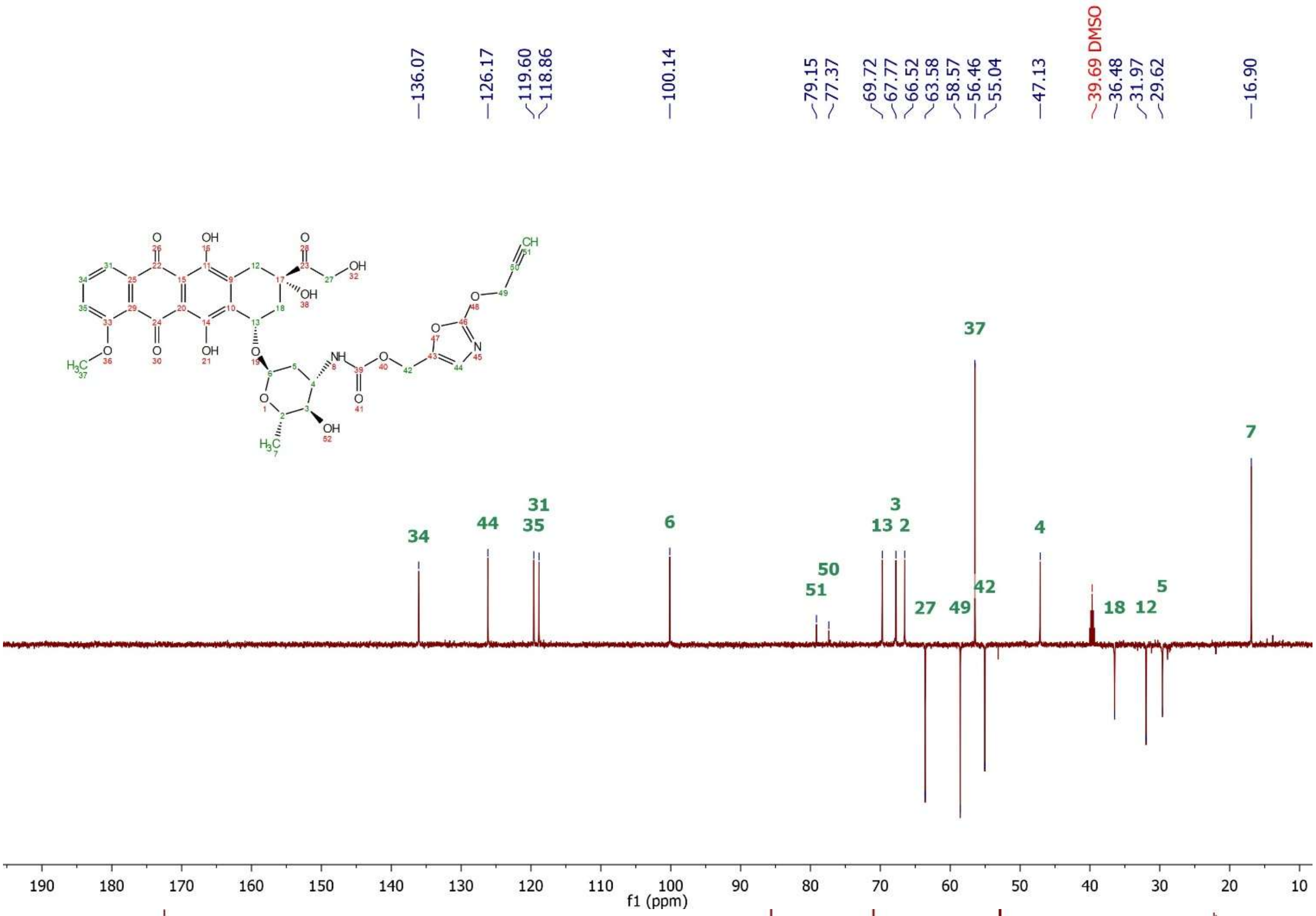
136.07
126.17
119.60
118.86
100.14
79.15
77.37
69.72
67.77
66.52
63.58
58.57
56.46
55.04
47.13
39.69 DMSO
36.48
31.97
29.62
16.90
37
7
34
44
31
35
6
3
13 2
4
50
51
42
5
27 49
18 12
f1 (ppm)

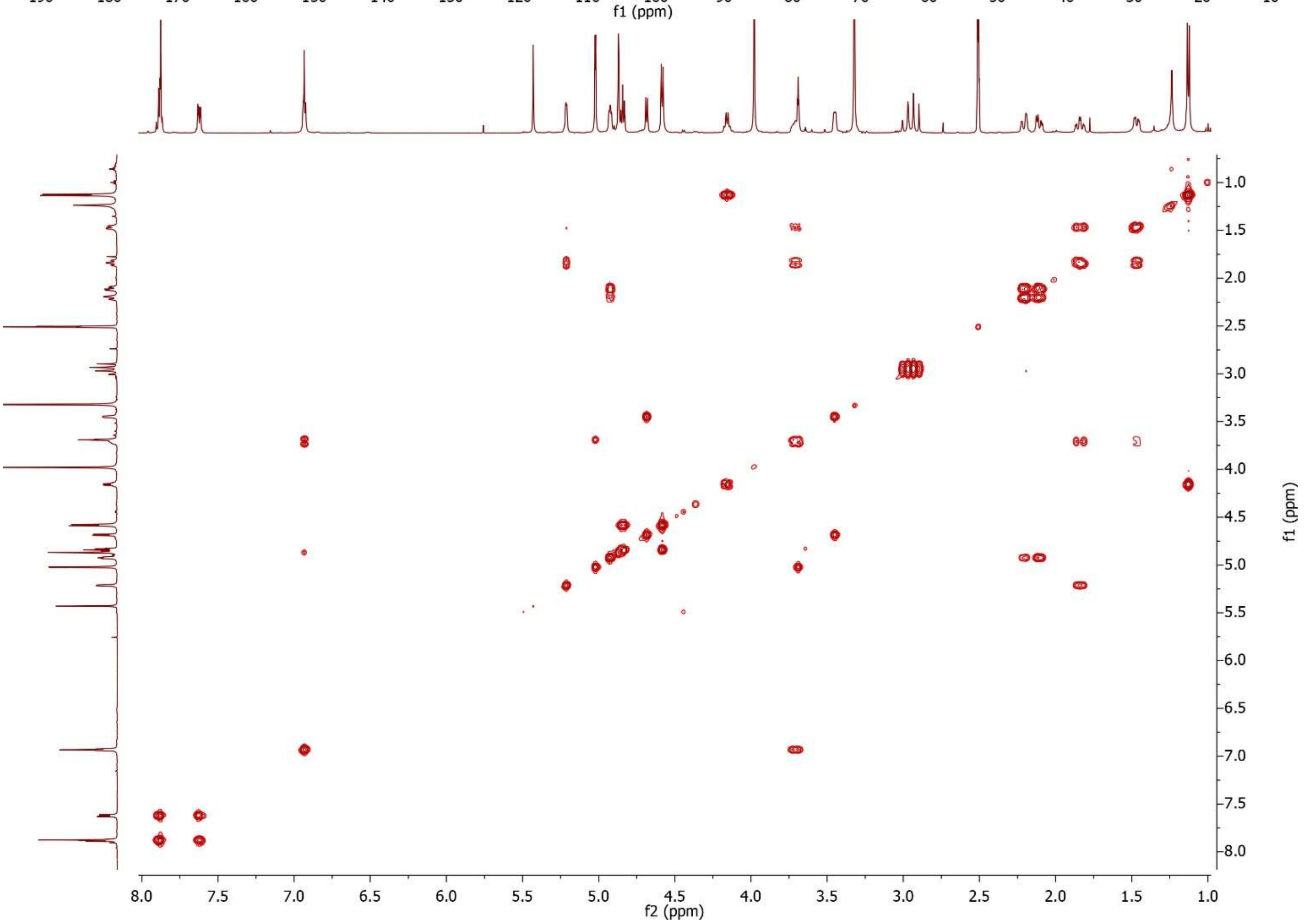
f2 (ppm)
f1 (ppm)

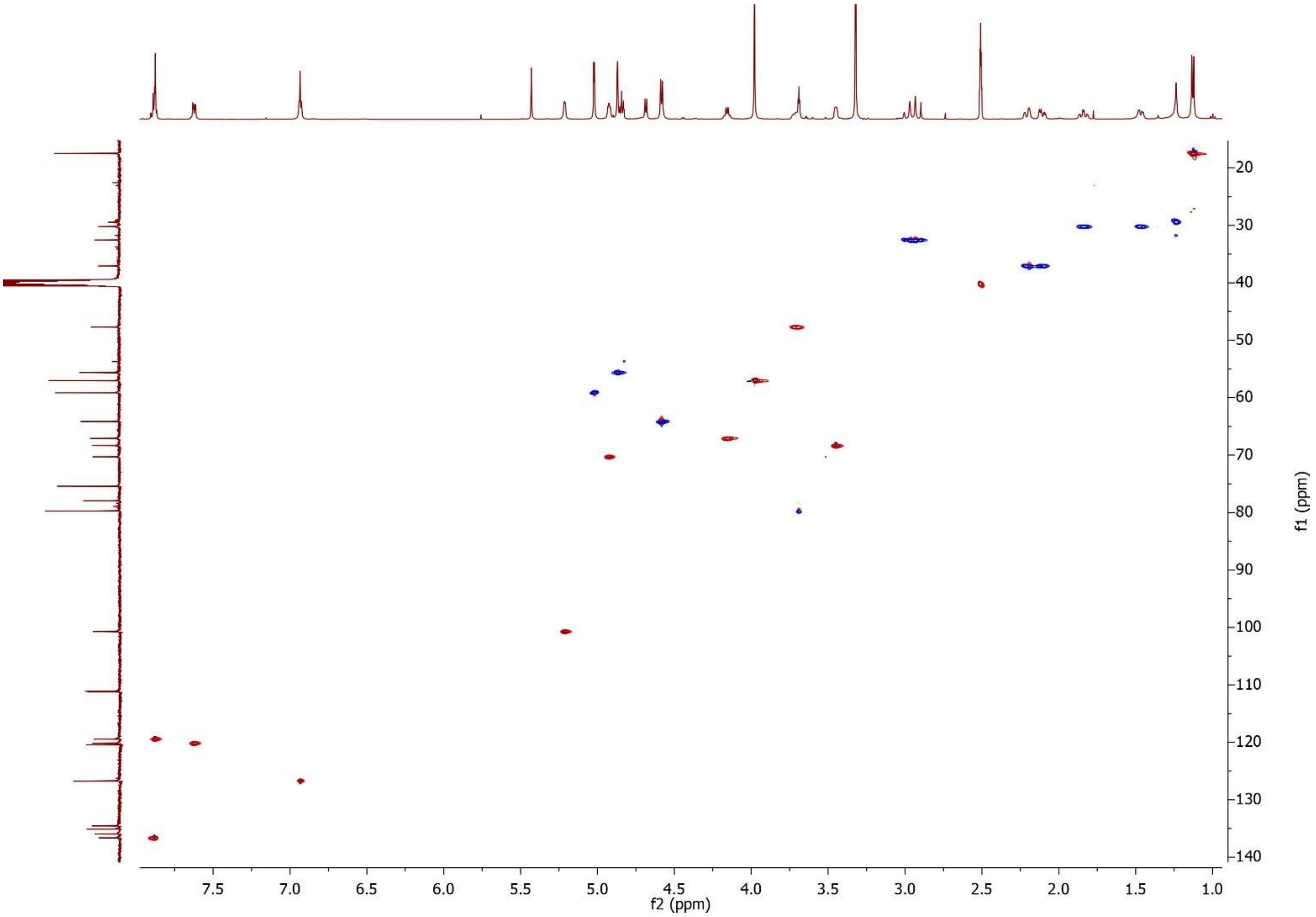
f2 (ppm)
f1 (ppm)

Fluorogenic reaction with Pd@Au and lipo-Pd@Au. 1 mL solution containing the desired concentration of **Pd@Au** or **lipo-Pd@Au** (0.02, 0.04, 0.08, 0.1 mg/mL) and 100 µM of **Poc-NBD** was prepared in an Eppendorf tube in PBS. The mixtures were irradiated for 15 min, operating at 1 W cm^{-2}. From this initial screen (see **Figure 4b and S8**), the concentration of 0.08 mg/mL NPs was selected for further testing in the absence (PBS) and presence of serum (PBS + 10% FBS) using 100 µM of **Poc-NBD**. Reactions were irradiated at room temperature for 15 min using a NIR laser and fluorescence intensity measured at different time points in a PerkinElmer EnVision 2101 multilabel reader (Ex/Em: 485/535 nm). The conversion values were calculated from fluorescence intensity measurements at λex/em = 485/535 nm using the fluorescence intensity of **1** (100 µM) as 100%. Negative controls: **2** (100 µM) with or without plasmonic NPs (0.08 mg/mL). Each experiment was performed at least in triplicate, and the values given correspond to the mean value ± SD of $n \geq 3$. The concentration of NBD (**1**) product (µM) was calculated based on the standard curve of NBD. DSPE-engineered **lipo-Pd@Au** were tested alongside to determine the effect of serum on freestanding **Pd@Au** and **lipo-Pd@Au**. The results are shown in **Figure S11b.**

Recyclability study of Pd@Au and lipo-Pd@Au. Pd@Au or **lipo-Pd@Au,** as appropriate, (0.08 mg) were added to a 1 mL solution of prodye **2** (**Poc-NBD)** at 100 µM in serum (PBS + 10% of FBS). The mixtures were irradiated at room temperature for 15 min (1 W cm^{-2}) using a NIR laser and reactions fluorescence intensity measured at 24 h by in a PerkinElmer EnVision 2101 multilabel reader (Ex/Em: 485/535 nm). **Pd@Au** or **lipo-Pd@Au** (0.08 mg) were recovered by centrifugation (8000 rpm, 10 min) and washed with distilled water. A freshly-prepared solution of **Poc-NBD** at 100 µM in serum was added to the **Pd@Au** or **lipo-Pd@Au**, the mixtures shaken were irradiated using a NIR laser (1 W cm^{-2}), and fluorescence measured at 15 min. This cycle was repeated 8 times. The results are shown in the **Figure S11c**.

HPLC-Monitored Kinetic Studies. Stock solutions of pro/drug (10 mM in DMSO) were diluted into 10% PBS/water to 200 µM. They were then diluted 1:1 into in 10% PBS/water with and without (-ve control) Pd@Au-lipo NPs (160 ng/mL in 10% PBS/water). Final concentration pro/drugs = 100 µM in 10% PBS/water+1% DMSO, Pd@Au-lipo NPs = 80 ng/mL. Reactions were kept at 37 ºC until each time point, then centrifuged at 21300 g for 3 mins, and a 100 µL aliquot removed and directly submitted for LC-MS analysis. Reaction vials were briefly sonicated and vortexed to ensure NPs were resuspended, and replaced at 37 ºC until the next time point. Samples were analysed by HPLC-MS using an Agilent Infinity II system (Poroshell 120 EC-C18 column) with binary mixtures made of A (0.1% formic acid, 99.9% H2O) and B (0.1% formic acid, 99.9% MeOH), flow = 1.0 mL/min), injection volume 20 µL, monitoring at 254 nm. Gradient: A/B = 50:50 to 5:95 over 4 min followed by a 1 min isocratic period A/B = 5:95. The curve in the main manuscript **Figure 7b** was generated by fitting the data to an exponential decay, $y = y_0 + A1*\exp(-(x-x_0)/t_1)$, using *Origin*. k was calculated as 1/t1.

3. BIOLOGICAL STUDIES

Cell culture. Human breast adenocarcinoma MDA-MB-231 cells, human colon carcinoma HCT116 cells (purchased from ECACC), and mammary epithelial MCF-10A cells were cultured in culture media supplemented with serum (10 % of FBS) and L-glutamine (2 mM). HCT116 cells were cultured in McCoy's 5A medium. MDA-MB-231 cells were cultured in DMEM media. MCF-10A cells were cultured in DMEM/F-12 (Sigma Cat No. 51448C) supplemented with 5% horse serum (Sigma Cat No. H1270), 10 ng/mL EGF (Sigma Cat No. E9644), 0.5 ug/mL hydrocortisone (Sigma Cat No. H6909), 1 ng/mL cholera toxin (Sigma Cat No. C8052), and 10 µg/mL insulin (Sigma Cat No. 19278). Each cell line was checked for mycoplasma before use and maintained in normoxic conditions at 37 ^{o}C and 5% CO_2. Cells were seeded in a 96-well plate at 20.000 cells/well for MDA-MB-231 or 30.000 cell/wells for HCT116 and MCF-10A and incubated for 24 h before treatment.

Study of the biocompatibility of lipo-Pd@Au. The tolerability of cells to **lipo-Pd@Au** were tested by performing dose-response studies in HCT116, MDA-MB-231, and MCF-10A cells. Cells were seeded in a 96-well plate at a density of 1.500 cells/well for MDA-MB-231 or 3.000 cell/wells for HCT116 and MCF-10A, then incubated for 24 h following for 5d of treatment. Each well was then replaced with 100 µL of fresh media containing **lipo-Pd@Au** at 0.2, 0.4, 0.6, 0.8, 1 and 1.2 mg/mL for all cell lines. After 7 days, PrestoBlue™ cell viability reagent (10 % *v*/*v*) was added to each well and the plate incubated for 90 min. Fluorescence emission was detected using a PerkinElmer EnVision 2101 multilabel reader (Ex/Em: 540/590 nm). Experiments were performed in both technical and biological triplicates. All conditions were normalized to the untreated cells (100 %). The results are shown in **Figure S12.**

Study of cell uptake of lipo-Pd@Au by ICP-MS. Cells were seeded in a 96-well plate at 20.000 cells/well for MDA-MB-231, or 30.000 cell/wells for HCT116 and MCF-10A and incubated for 24 h before treatment. An intracellular metal content study was carried out by incubating each cells with **lipo-Pd@Au** (80 µg/mL of metal content) for 6h. After the media containing **lipo-Pd@Au** were removed and the adhered cells were washed twice with PBS, cells were detached by trypsinization and centrifuged to further eliminate extracellular NPs by discarding the supernatant. Cell pellets were digested (2 % aqua regia) and analyzed by ICP-MS. Quantification of Au and Pd content inside the cell (nmol of metal/cell). Error bars: ±SD, n = 3. The results are shown in **Figure S15.**

Study of the biocompatibility of prodrug 9b and DOX in MDA-MB-231 cells & Cytotoxicity dose-response curves.

Human breast adenocarcinoma cells (MDA-MB-231) were cultured in DMEM with GlutaMAX, supplemented with serum (10 % FBS). Each cell line was checked for mycoplasma before use and maintained in normoxic conditions at 37 ºC and 5% CO_2.

Cells were seeded in a 96-well plate format at 2,000 (MDA-MB-231) cells / well and incubated for 48 h before treatment. Each well was then replaced with fresh media (95 µL) followed by the compound of interest (5 µL in 2% v/v DMSO / medium at 20× the final concentration). Control wells were incubated with DMSO (0.1 % v/v). Cells were then incubated 5 d. Cell viability was measured on day 5 by adding PrestoBlue® cell viability reagent (10 µL) to each well and incubating for 90 min. Fluorescence emission was detected using a Tecan *infinite 200Pro* multilabel reader (Ex/Em: 540 nm/590 nm). All conditions were normalized to DMSO control wells (100%), media alone (0% viability) and curves fitted with GraphPad Prism 9 using a dose-response Inhibition model. Error bars: ±SD, n = 3. The results are shown in **Figure S24.**

Bioorthogonal uncaging of prodrug 8-9a-b and photothermal therapy in cell culture. HCT116, and MDA-MB-231 cells were seeded in 96-well plates (HTC116 30.000 cells/well; MDA-MB-231 20.000 cells/well) and incubated for 24 h before treatment. Then, **lipo-Pd@Au** were added (80 µg/mL) to the cells and incubated for 6 h at 37 °C and 5% CO_2. After incubation, cells were washed twice with PBS to eliminate extracellular NPs and thus, to evaluate only the effects of intracellular NPs. Each of the desired wells was then replaced with fresh media containing: **8** (100µM) or **9a-b** (10µM); and incubated for 1 h at 37 °C and 5% CO_2. Then, each wells were irradiated at room temperature for 5 min (1 W cm^{-2}) using a NIR laser. Cells were washed twice with PBS, fresh medium was added and incubated for 24 h at 37 °C and 5% CO_2. Cells were incubated with **5FU** (100µM) or **DOX** (10 µM) for 2 d. All experiments, including the untreated cells, contained 0.1% v/v of DMSO and were performed in both technical and biological triplicates. PrestoBlue™ cell

viability reagent (10% v/v) was added to each well and the plates were incubated for 90 min. Fluorescence emission was detected and results normalized as described above.

Quantification and Statistical Analysis

All statistical methods used in the paper are described in the figure legends and, where indicated, additional details are provided in the method details. Statistics were computed using GraphPad Prism 9.

4. *IN VIVO* EXPERIMENTS

Zebrafish husbandry. Animals were bred and raised at the Institute of Genetics and Cancer, University of Edinburgh, in accordance with the Animals in Scientific Procedures Act 1986, amended in 2013, European Directive 2010/63/EU and under British Home Office project licences PP7283023 and P8F7F7E52. Embryos were obtained through natural spawning from the transparent casper or *fli1:GFP* casper mutant fish (mitfa$^{w2/w2}$; mpv17$^{a9/a9}$, Tg(*fli1:GFP*))[9, 10], as indicated. Embryos were maintained at low density (~50-100/50ml E3 zebrafish medium composed for 5mM NaCl, 0.17mM KCl, 0.33 mM $CaCl_2$, 0.33 mM $MgSO_4$) at 28 °C with a 14h light/10h dark cycle. For xenograft studies, on reaching larval stage 2 days post-fertilisation (dpf), animals were microinjected with cancer cells at room temperature then maintained at 32 °C (to allow survival of human cells) as before, until 5 dpf.

Colon Cancer Zebrafish Xenograft Experiments. Injection range of 1-3 nl in the yolk sac or into PVS, as indicated, of internalized **lipo-Pd@Au** into HCT116 cancer cells (catalytically active, **lipo-Pd@Au/cells**) or **cancer cells** (catalytically inactive, **cells**; negative control) was carried out at 2 dpf. **Cell Culture**: HCT116 cells at 70-80% confluence cultured in T-75 flask were incubated with fresh McCoy's 5A media with **lipo-Pd@Au** (0.08 mg/mL). Control T-75 flask was processed without NPs (cancer cells). After 12 h, the media was removed from the flask, washed with PBS (3 times), and incubated with Hoechst 33342 (Abcam, ab228551) (1:10000) in PBS for 10 min at room temperature in darkness. Where applicable, this was followed by incubation with DiD (Vybrant DiD, Invitrogen) (final dye concentration of 5 µM) in medium at 37 °C for 20 min. After this period, cells were dissociated with trypsin at 37 °C, followed by the addition of two volumes of pre-warmed complete culture media to inactivate trypsin. Cells were centrifuged for 4 min at 300g and re-suspended in complete cell culture medium. Neubauer chamber cell counts were performed to assess the cell density of viable cells. Cell suspension was centrifuged one last time for 4 minutes at 300 g and res-suspended in PBS/5% FCS to a final concentration of 0.5×10^6 cells/µL. **Zebrafish Xenograft Injection.** Larvae were anaesthetised with ethyl 3-aminobenzoate methanesulfonate solution (0.1 g/L, Tricaine, MS-222) in PBS and positioned on 2.5% agarose mould prior injection. Then, **lipo-Pd@Au/cells** or **cells**, as appropriate, were microinjected into the yolk sac of 48 hours post fertilization (hpf) anesthetized zebrafish larvae using Intrafil borosilicate glass capillaries attached to Picospritzer III microinjection dispense system. After injection, xenografts were transferred from anaesthetics to fresh E3 medium and placed at 32 °C to allow human cell lines implantation. After 2 hours post injection (hpi), injected zebrafish were screened for succesful transplantation and sorted into classes according to the tumour size. Xenografts were then randomly distributed into the different treatment groups. **Zebrafish Xenograft Prodye (7a-b) Administration**. Xenografts were randomly distributed in two groups: A) **Cells** (HCT116 cells + Hoechst): 1% DMSO in E3 or 2.5 µM of **7a** or **7b** in E3 with 1% DMSO were added and incubated approx. 18 hours prior NIR irradiation (30 sec, 1 W cm^{-2}); and B) **Lipo-Pd@Au/cells** (HCT116 cells + lipo-Pd@Au + Hoechst): 1% DMSO in E3, 2.5 µM of **7a** or **7b** in E3 with 1% DMSO and then NIR irradiation (30 sec, 1 W cm^{-2}). Control experiments without NIR irradiation were also carried out. The zebrafish were then mounted in 1% low melting point agarose on glass bottom 6-well-plate and imaged using an Olympus FV3000 Confocal Laser Scanning Microscope. The setting of the confocal microscope was as follows: blue channel, λexc= 405 nm and λem= 460-490 nm; green channel, λexc= 488 nm and λem= 514-553 nm. **Zebrafish Xenograft Prodrug (8) Administration.** Xenografts were randomly distributed in two groups: A) **Cells** (HCT116 cells + DiD + Hoechst): 1% DMSO in E3, 200 µM of **8** in E3 with 1% DMSO and then NIR irradiation (30 sec, 1 W cm^{-2}); and B) **Lipo-Pd@Au/cells** (HCT116 cells + lipo-Pd@Au + DiD + Hoechst): 1% DMSO in E3, 200 µM of **8** in E3 with 1% DMSO and then NIR irradiation (30 sec, 1 W cm^{-2}). Control experiments without NIR irradiation were also carried out. The zebrafish were then imaged using a confocal microscope as above. The setting of the confocal microscope was as follows: blue channel, λexc= 488 nm and λem= 514-553 nm; red channel, λexc= 644 nm and λem= 650-750 nm. Settings for acquisition and reconstruction were identical in all images. Image analyses were performed in Fiji/ImageJ.

Breast Cancer Zebrafish Xenograft Experiments. 200-300 MDA-MB-231 cancer cells with internalised **Lipo-Pd@Au** (catalytically active, **Lipo-Pd@Au** /cells) or MDA-MB-231 cells alone (catalytically inactive, cells; negative control) were injected into PVS of 2 dpf zebrafish larvae. **Cell Culture**: MDA-MB-231 cells at 70-80% confluence cultured in T-75 flask were incubated with fresh DMEM media with **Lipo-Pd@Au** (0.08 mg/mL). Control T-75 flask was processed without NPs (cancer cells). After 4 h, the media was removed from the flask, washed with PBS (3 times), and incubated with Hoechst 33342 (1:10000) in PBS for 10 min at room temperature in darkness. This was followed by incubation

with DiD (final dye concentration of 5 μM) in medium at 37 °C for 20 min. After this period, cells were dissociated with trypsin at 37 °C, followed by the addition of two volumes of pre-warmed complete culture media to inactivate trypsin. Cells were centrifuged for 4 min at 300g and re-suspended in complete cell culture medium. Neubauer chamber cell counts were performed to assess the cell density of viable cells. Cell suspension was centrifuged one last time for 4 minutes at 300 g and res-suspended in PBS/5% FCS to a final concentration of 0.5 × 10^6 cells/μL. **Zebrafish Xenograft Injection**: Larvae were anaesthetised with ethyl 3-aminobenzoate methanesulfonate solution (0.1 g/L, Tricaine, MS-222) in PBS and positioned on 2% agar mould prior injection. Then, **Lipo-Pd@Au**/cells or cells, as appropriate, were microinjected into the PVS of 2 dpf anesthetized zebrafish larvae using borosilicate glass capillaries attached to Picospritzer III microinjection dispense system. After injection, xenografts were transferred from anaesthetics to fresh E3 medium and placed at 32°C to allow human cell lines implantation. After 2 hours post injection (hpi), injected zebrafish were screened for succesful transplantation and sorted into classes according to the tumour size. Xenografts were then randomly distributed into the different treatment groups. **Zebrafish Xenograft Prodrug** (**9b**) Administration: Xenografts were randomly distributed in two groups: A) Cells (MDA-MB-231 cells + DiD + Hoechst): 1% DMSO in E3, 10 μM of **9b** in E3 with 1% DMSO and then incubated for 38h; and B) **Lipo-Pd@Au**/cells (MDA-MB-231 cells + **Lipo-Pd@Au** + DiD +Hoechst): 1% DMSO in E3, 10 μM of **9b** in E3 with 1% DMSO and then incubated for 38h (4dpf). Control experiments without prodrug were also carried out. The zebrafish were then imaged using a confocal microscope as above. The setting of the confocal microscope was as follows: blue channel, λexc= 405 nm and λem= 430-470 nm; greed channel, λexc= 488 nm and λem= 575-612 nm; red channel, λexc= 647 nm and λem= 650-750 nm. Settings for acquisition and reconstruction were identical in all images. Image analyses were performed in Fiji/ImageJ.

Imaging and tumour size/number cancer cells Quantification. All images were obtained in an Olympus FV3000 Confocal Laser Scanning Microscope, generally at 20x and 40x objectives. Z-stack acquisition was performed to capture optical sections through the zebrafish embryos, with step sizes optimized for the resolution and depth for each zebrafish. The number of cells was quantified with ImageJ software Cell counter plugin. The number of total HCT116 or MDA-MB-231 cells (Hoechst 33342) = AVG (3 slices Zfirst, Zmiddle, Zlast) x total number slice/1.5.

Statistical Analysis: GraphPad Prism software (version 9.5.1) was used for statistical analyses. All datasets were tested for normal distribution. Normally distributed datasets were analyzed with unpaired t test or one-way analysis of variance (ANOVA) followed by post-hoc test for multiple comparisons. Non-normally distributed datasets were analyzed by Mann-Whitney test or Kruskal-Wallis tests, followed by post-hoc test for multiple comparisons. For all the statistical analysis, P-value (P) is from a two-tailed test with a confidence interval of 95%. Statistical differences were considered significant whenever P<0.05 and statistical output was represented by stars as follows: non-significant (ns)>0.05, *≤0.05, **≤0.01, ***≤0.001 and ***≤0.0001. All the graphs presented the results as Avg ± standard error of the mean (SEM).

5. SUPPLEMENTARY REFERENCES

(1) Adam, C.; Bray, T. L.; Perez-Lopez, A. M.; Tan, E. H.; Rubio-Ruiz, B.; Baillache, D. J.; Houston, D. R.; Salji, M. J.; Leung, H. Y.; Unciti-Broceta, A. A 5-FU Precursor Designed to Evade Anabolic and Catabolic Drug Pathways and Activated by Pd Chemistry In Vitro and In Vivo. *Journal of Medicinal Chemistry* **2022**, *65* (1), 552-561, Article. DOI: 10.1021/acs.jmedchem.1c01733.

(2) Ortega-Liebana, M. C.; Porter, N. J.; Adam, C.; Valero, T.; Hamilton, L.; Sieger, D.; Becker, C. G.; Unciti-Broceta, A. Truly-Biocompatible Gold Catalysis Enables Vivo-Orthogonal Intra-CNS Release of Anxiolytics. *Angewandte Chemie-International Edition* **2022**, *61* (1), e202111461, Article. DOI: 10.1002/anie.202111461.

(3) Bray, T. L.; Salji, M.; Brombin, A.; Perez-Lopez, A. M.; Rubio-Ruiz, B.; Galbraith, L. C. A.; Patton, E. E.; Leung, H. Y.; Unciti-Broceta, A. Bright insights into palladium-triggered local chemotherapy. *Chemical Science* **2018**, *9* (37), 7354-7361. DOI: 10.1039/c8sc02291g.

(4) Ortega-Liebana, M. C.; Hueso, J. L.; Arenal, R.; Santamaria, J. Titania-coated gold nanorods with expanded photocatalytic response. Enzyme-like glucose oxidation under near-infrared-illumination. *Nanoscale* **2017**, *9* (5), 1787-1792. DOI: 10.1039/c6nr06300d.

(5) Zheng, Z.; Tachikawa, T.; Majima, T. Plasmon-Enhanced Formic Acid Dehydrogenation Using Anisotropic Pd-Au Nanorods Studied at the Single-Particle Level. *J. Am. Chem. Soc.* **2015**, *137* (2), 948-957. DOI: 10.1021/ja511719g.

(6) Su, G.; Jiang, H.; Zhu, H.; Lv, J.-J.; Yang, G.; Yan, B.; Zhu, J.-J. Controlled deposition of palladium nanodendrites on the tips of gold nanorods and their enhanced catalytic activity. *Nanoscale* **2017**, *9* (34), 12494-12502, Article. DOI: 10.1039/c7nr04046f.

(7) Braun, J.; Ortega-Liebana, M.; Unciti-Broceta, A.; Sieber, S. A Pd-labile fluoroquinolone prodrug efficiently prevents biofilm formation on coated surfaces. *ORGANIC & BIOMOLECULAR CHEMISTRY* **2024**, *22*, 1998-2002. DOI: 10.1039/d4ob00014e.

(8) Perez-Lopez, A. M.; Rubio-Ruiz, B.; Sebastian, V.; Hamilton, L.; Adam, C.; Bray, T. L.; Irusta, S.; Brennan, P. M.; Lloyd-Jones, G. C.; Sieger, D.; et al. Gold-Triggered Uncaging Chemistry in Living Systems. *Angewandte Chemie-International Edition* **2017**, *56* (41), 12548-12552. DOI: 10.1002/anie.201705609.

(9) Lawson, N. D.; Weinstein, B. M. In vivo imaging of embryonic vascular development using transgenic zebrafish. *Developmental Biology* **2002**, *248* (2), 307-318, Article. DOI: 10.1006/dbio.2002.0711.

(10) White, R. M.; Sessa, A.; Burke, C.; Bowman, T.; LeBlanc, J.; Ceol, C.; Bourque, C.; Dovey, M.; Goessling, W.; Burns, C. E.; et al. Transparent adult zebrafish as a tool for in vivo transplantation analysis. *Cell Stem Cell* **2008**, *2* (2), 183-189, Article. DOI: 10.1016/j.stem.2007.11.002.

7. SUPPLEMENTARY FIGURES

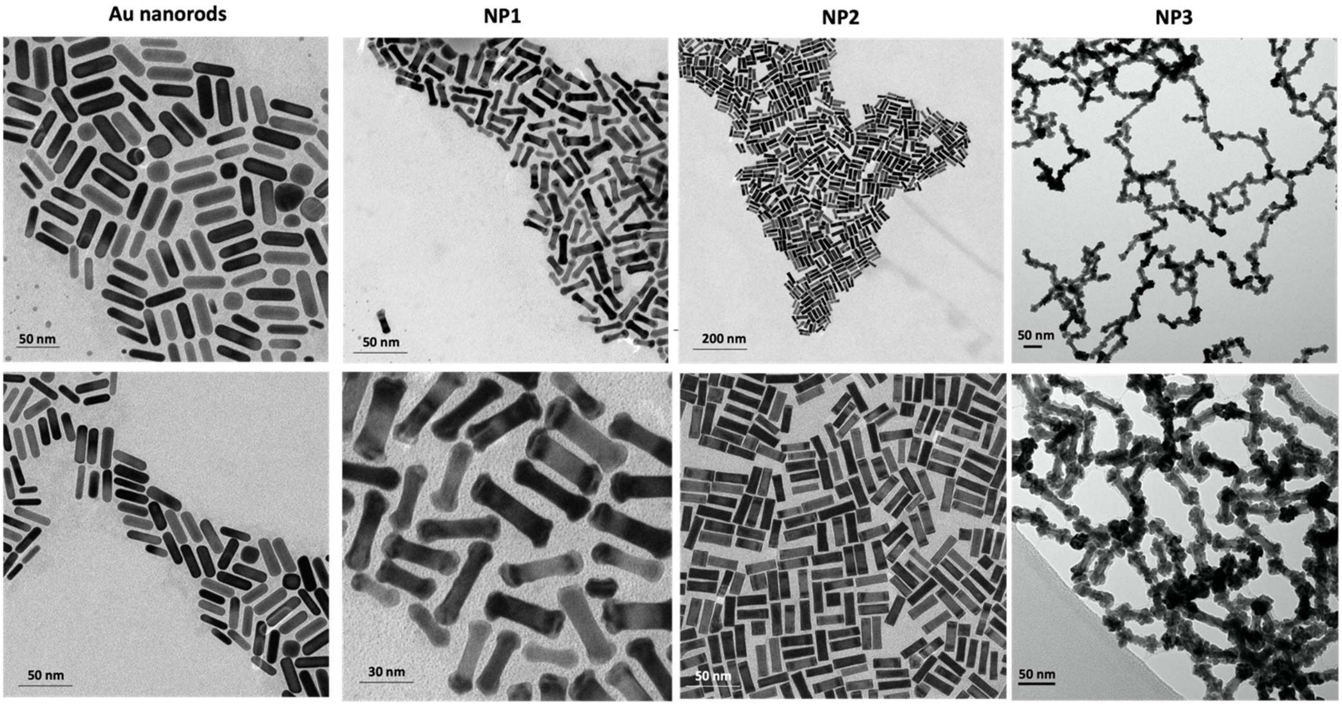

Figure S1. TEM characterization. TEM images of Au nanorods, NP1, NP2 and NP3 at different magnifications.

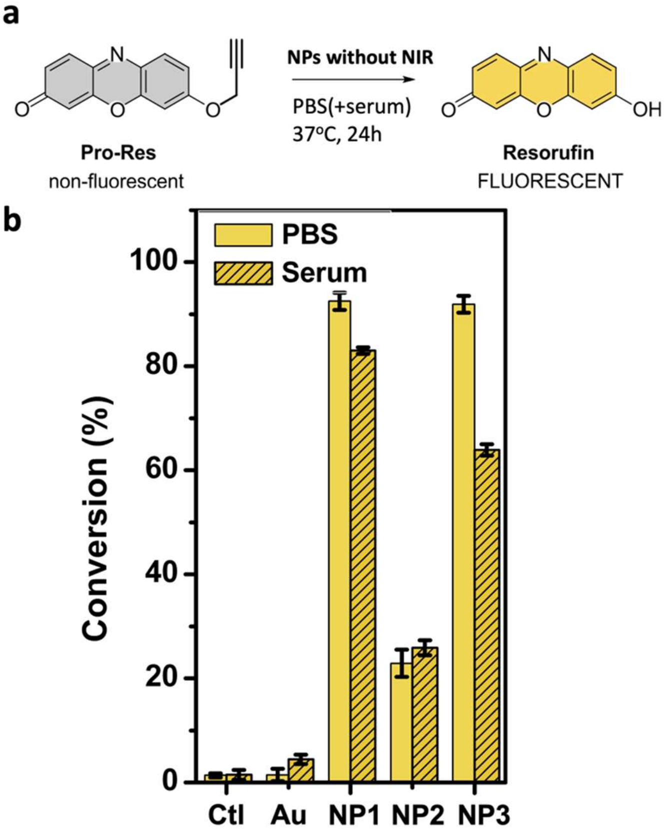

Figure S2. Catalytic properties of Au nanorods and bimetallic NP1, NP2 and NP3. a, Fluorogenic assay: non-fluorescent **Pro-Res** (100 μM) and **Au nanorods** (Au) or **NP1-3** (40 μg/mL) in PBS and PBS + 10% FBS (serum). **b**, Comparative study of the conversion efficiencies after 24h incubation at 37 °C without NIR irradiation. The conversion values were calculated from fluorescence intensity measurements at $\lambda_{ex/em}$ = 550/580 nm using a standard curve of the fluorescence intensity of resorufin. Negative controls: **Pro-Res** (100 μM) without catalysts. The error bars are ± SD (n = 3).

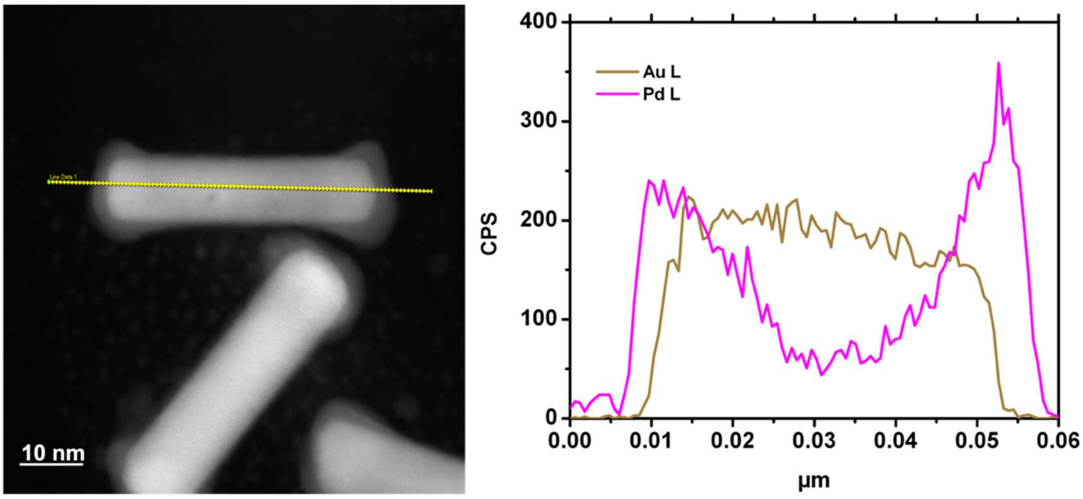

Figure S3. HAADF-STEM images and corresponding STEM-EDS analysis: line scanning elemental profiles recorded along the line as marked in the NP1. CPS = counts per second.

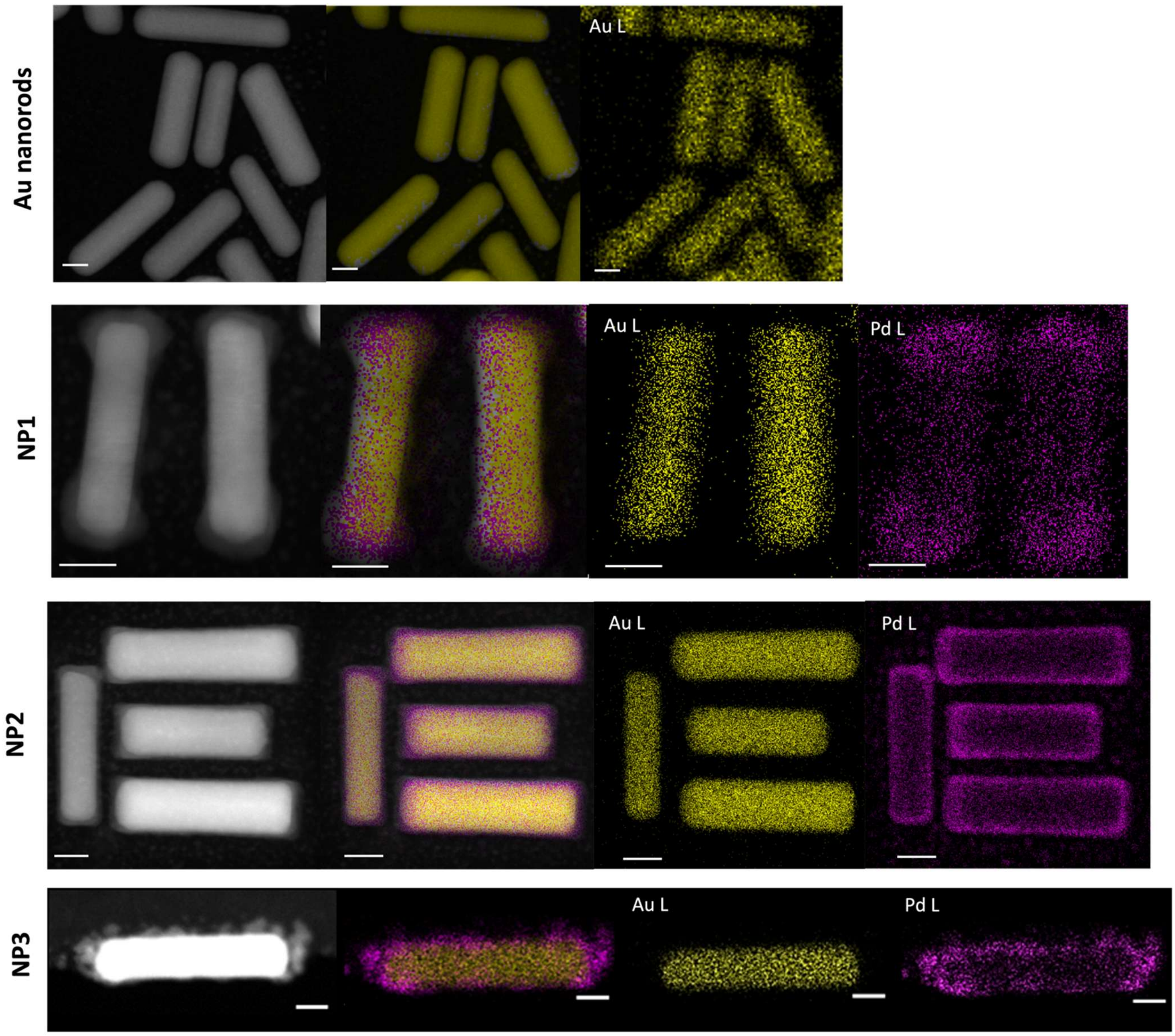

Figure S4. HAADF-STEM images and corresponding STEM-EDS elemental maps of Au + Pd, Au and Pd: of Au nanorods, NP1, NP2 and NP3, respectively. The scale bars in all the images represent 10 nm.

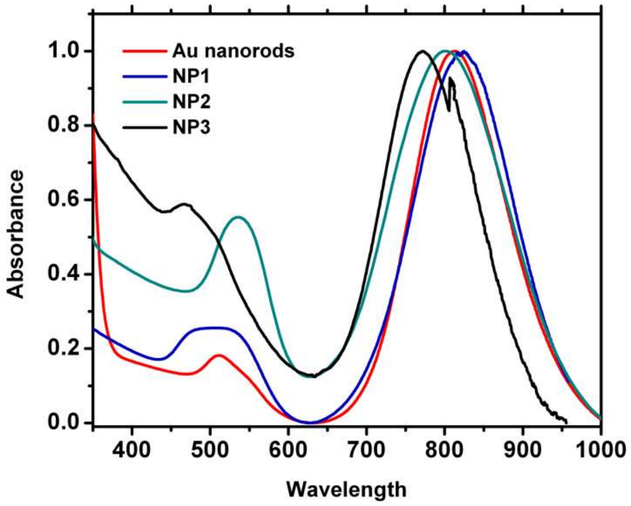

Figure S5. UV-Visible-NIR absorption spectra of as-synthesized **Au nanorods, NP1, NP2** and **NP3**, showing a maximum surface plasmon resonance peak at approx. 810 nm, 820 nm, 800 nm, and 770 nm, respectively.

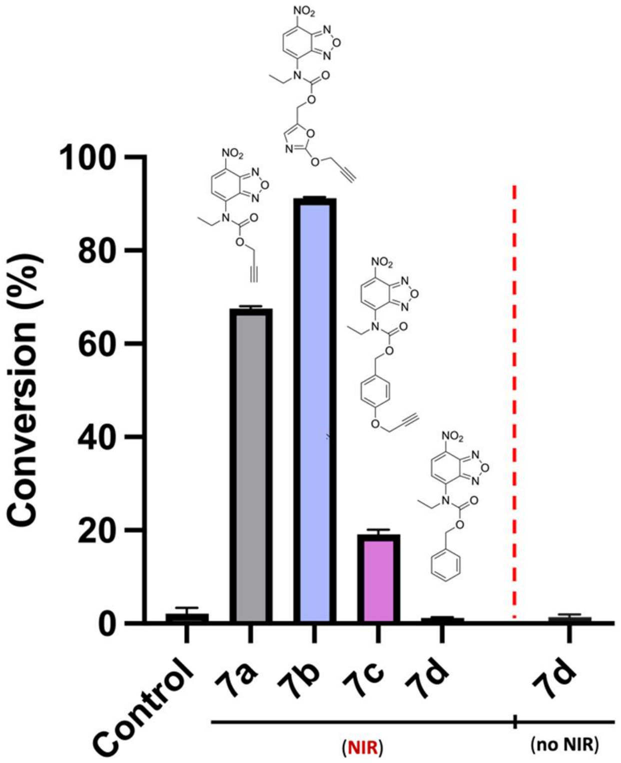

Figure S6. Fluorogenic reaction of **7a-d** (100 μM) and lipo-Pd@Au (80 μg/mL) in serum (pH 7.4) with NIR irradiation (5 min); and for **7d**, the reaction without NIR after 3 days of incubation at 37°C in serum.

.

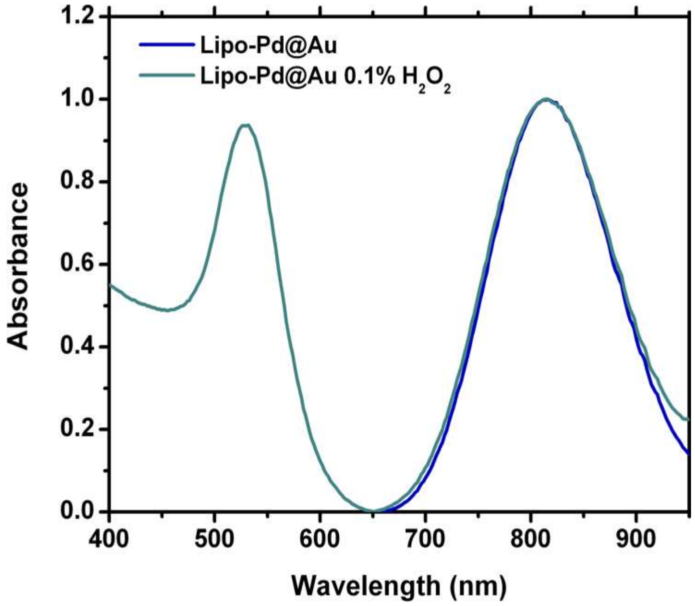

Figure S7. UV-vis-NIR absorption spectra of **lipo-Pd@Au** in water and in water containing 0.1% of H_2O_2 after 3 days of incubation.

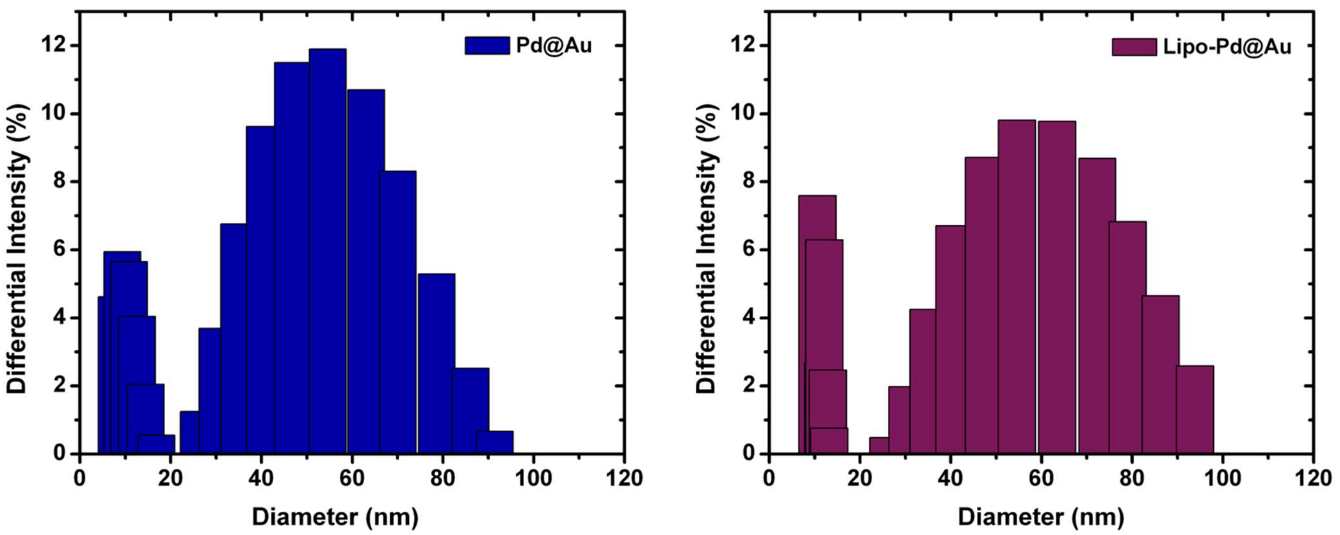

Figure S8. DLS size distribution of **Pd@Au** and **lipo-Pd@Au.**

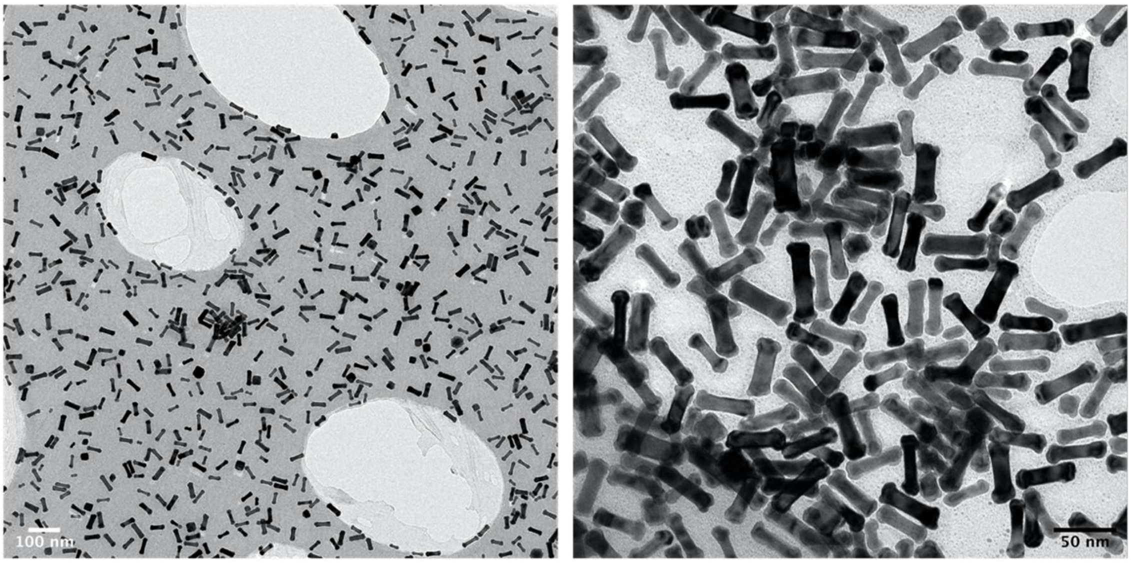

Figure S9. TEM characterization. TEM images of **lipo-Pd@Au.**

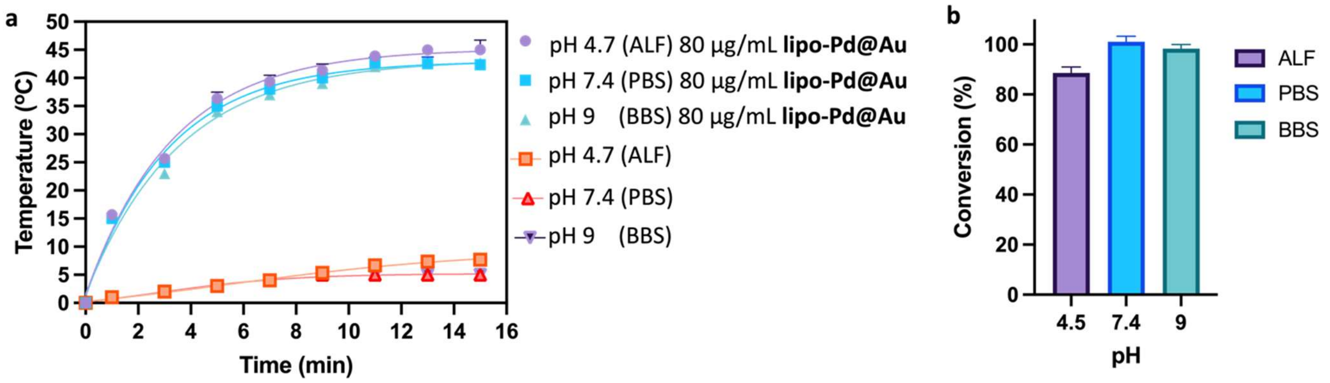

Figure S10. a, Heating efficiency of **lipo-Pd@Au** (80 μg/mL) in artificial lysosomal fluid (ALF, pH 4.5), PBS (pH 7.4) and borate buffered saline (pH 9) with irradiation of 808 nm wavelength laser ($1W/cm^2$) for 15 min. **b**, Fluorogenic reaction of **7b** (100 μM) and **lipo-Pd@Au** (80 μg/mL) in different media after 10 min of NIR irradiation. Conversion (%) was measured from fluorescence intensity measurements (λex/em= 485/535 nm) and calculated using a standard curve of 1.

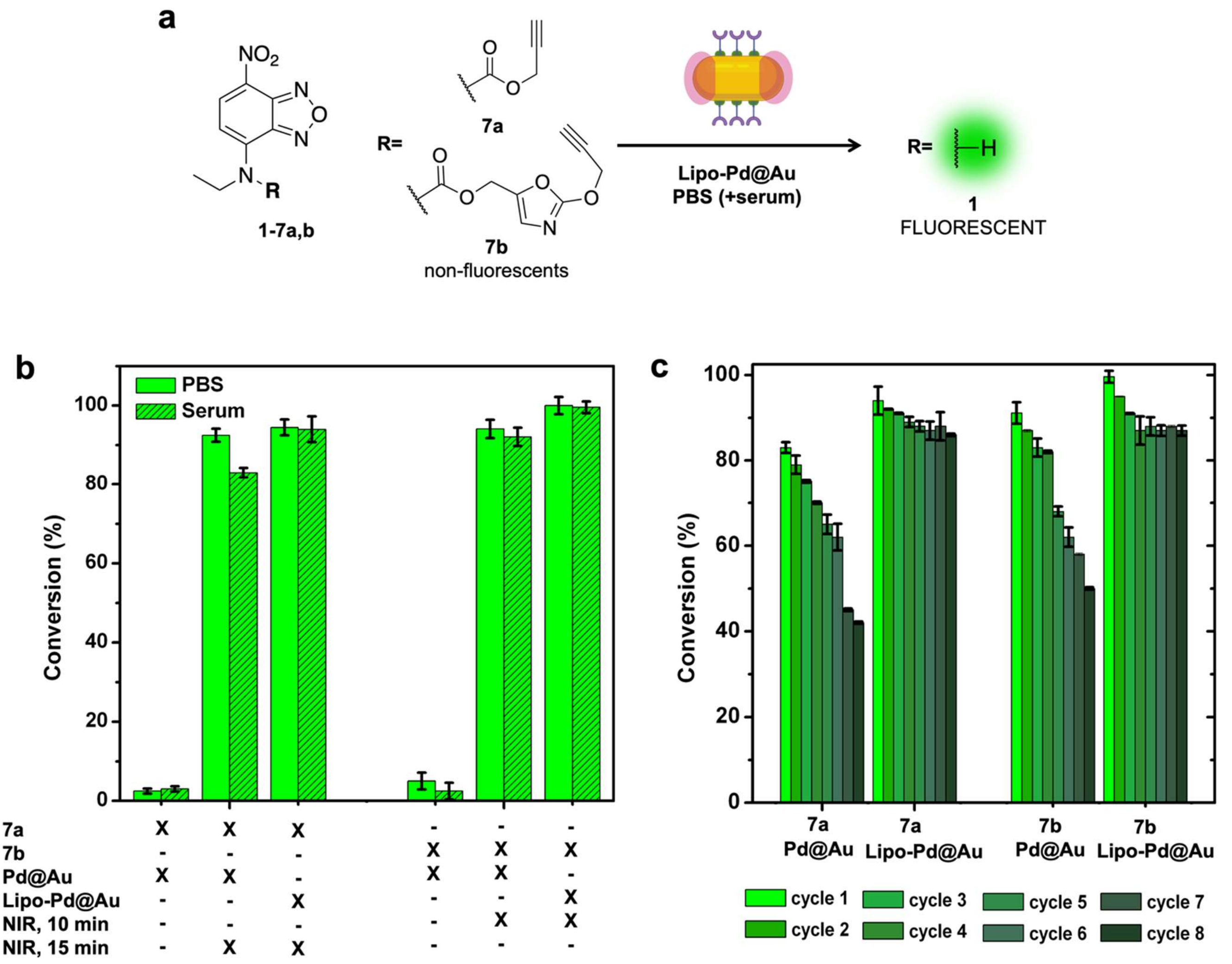

Figure S11. a, Plasmonic Pd@Au-triggered conversion of prodyes **7a,b** into dye **1. b**, Fluorogenic reaction of **7a** or 7b (100 μM) and Pd@Au or lipo-Pd@Au (80 μg/mL) in PBS and serum (pH 7.4) with or without NIR irradiation. Conversion (%) was measured at different time points from fluorescence intensity measurements (λex/em= 485/535 nm) and calculated using a standard curve of **1. c**, **Recycling test. Pd@Au** or **lipo-Pd@Au** (80 μg/mL) in serum (pH 7.4) were recovered after each reaction cycle and re-used. Conversion (%) was measured at 15 min of NIR irradiation.

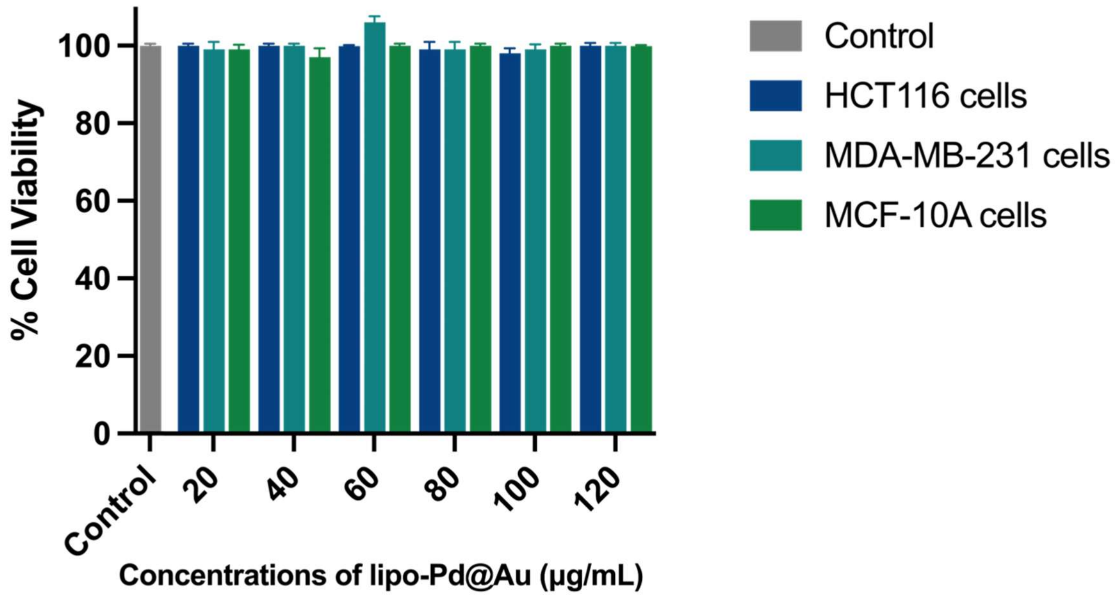

Figure S12. Cell viability study of the biocompatibility of lipo-Pd@Au in MDA-MB-231 cells, HCT116 cells, and MCF-10A cells. Cell viability study after 7 d treatment with 20, 40, 60, 80, 100 and 120 µg/mL of **lipo-Pd@Au** in different cell lines. Error bars: ± SD from n = 3.

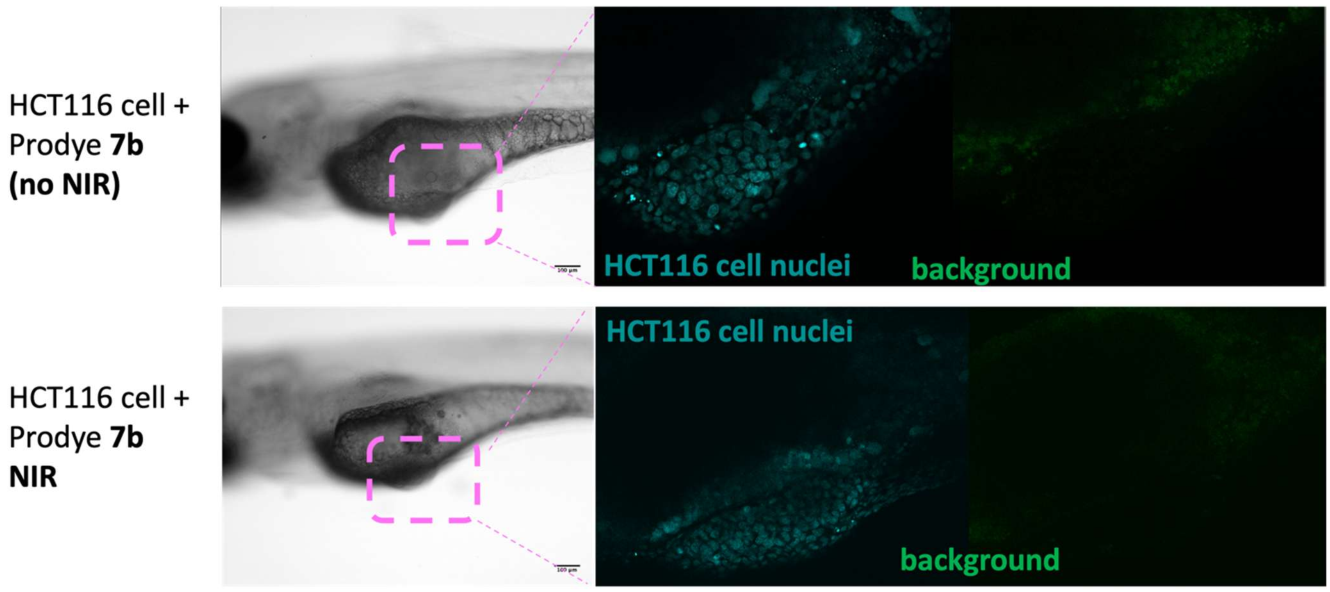

Figure S13. Negative controls of prodye decaging experiments in zebrafish xenografts in the absence of of lipo-Pd@Au. HCT116 cells were fluorescently labeled with Hoechst 33342 (shown in blue) and injected into the PVS in 2-dpf zebrafish larvae. Zebrafish xenografts were randomly distributed into treatment groups, treated with **7b** in E3 medium and analyzed after without and with NIR irradiation by confocal microscopy. Confocal analysis of the **non-release** of green fluorescent dye **1** from precursor **7b** in the HTC116 xenograft of a zebrafish larvae loaded **without lipo-Pd@Au** and Hoechst 33342 without NIR irradiation (top) or with NIR irradiation for 30 s (bottom). N=6. Scale bars = 100 µm.

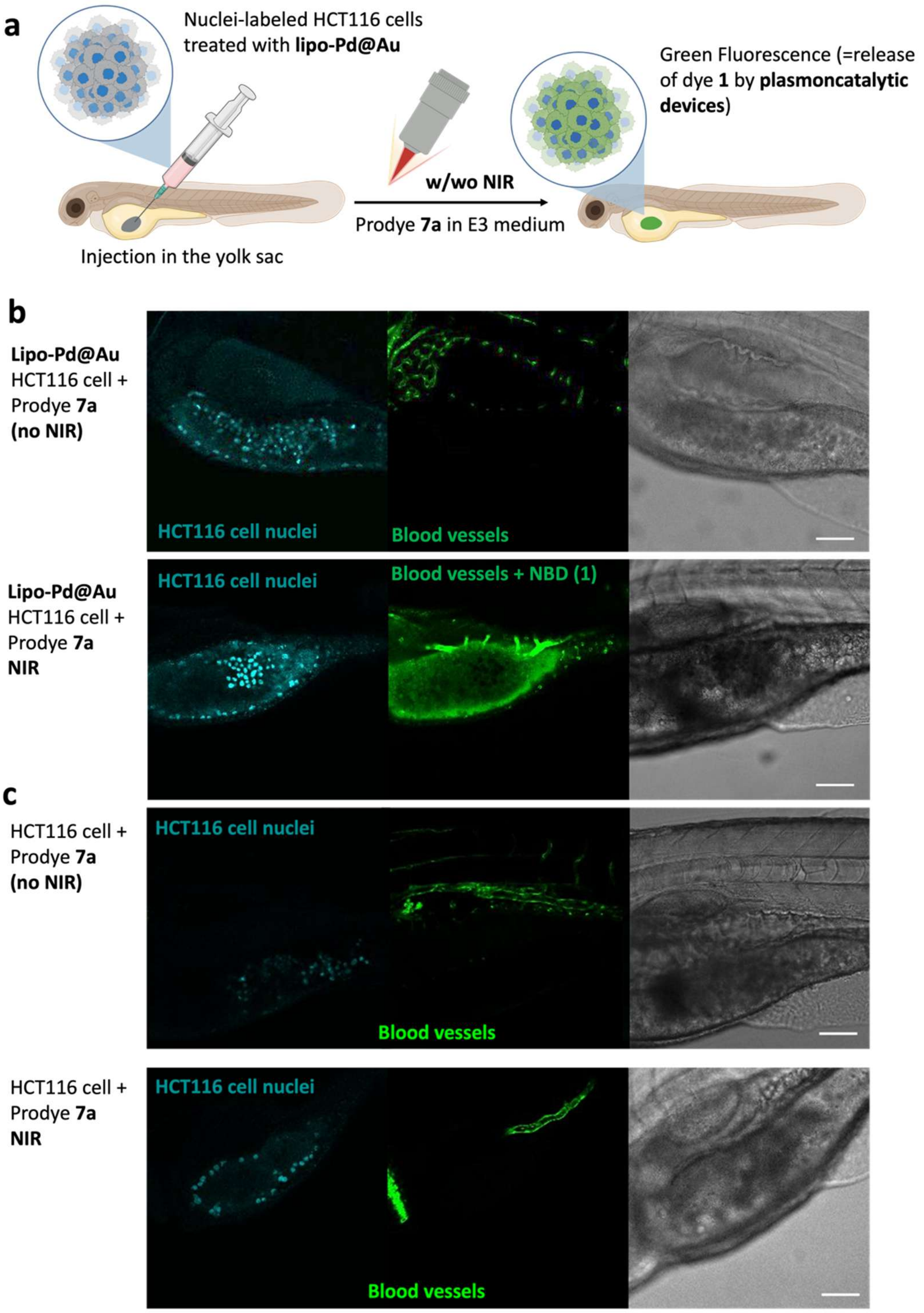

Figure S14. a, NIR-triggered plasmonic lipo-Pd@Au-mediated prodye decaging in zebrafish xenografts, created with BioRender.com**.** HCT116 cells were pre-incubated with lipo-Pd@Au, fluorescently labeled with Hoechst 33342 (in cyan) and injected into the yolk sac in 2-dpf (*fli1:GFP*) zebrafish larvae**.** Zebrafish xenografts were randomly distributed into treatment groups, treated with 7a in E3 medium and analyzed after NIR irradiation by confocal microscopy. **b**, Confocal analysis of the release of green fluorescent dye 1 from precursor **7a** in the HCT116 xenograft of a zebrafish larvae loaded with lipo-Pd@Au and Hoechst without NIR irradiation (top) or with NIR irradiation for 30 s (bottom). N= 4. Scale bars = 100 µm. **c**, Negative controls of prodye decaging experiments in zebrafish xenografts in the absence of of lipo-Pd@Au. HCT116 cells were fluorescently labeled with Hoechst 33342 (shown in blue) and injected into the yolk sac in 2-dpf (*fli1:GFP*) zebrafish larvae. Zebrafish xenografts were randomly distributed into treatment groups, treated with **7a** in E3 medium and analyzed after without and with NIR irradiation by confocal microscopy. Confocal analysis of the **non-release** of green fluorescent dye **1** from precursor **7a** in the HTC116 xenograft of a zebrafish larvae loaded **without lipo-Pd@Au** and Hoechst 33342 without NIR irradiation (top) or with NIR irradiation for 30 s (bottom). N=4. Scale bars = 100 µm.

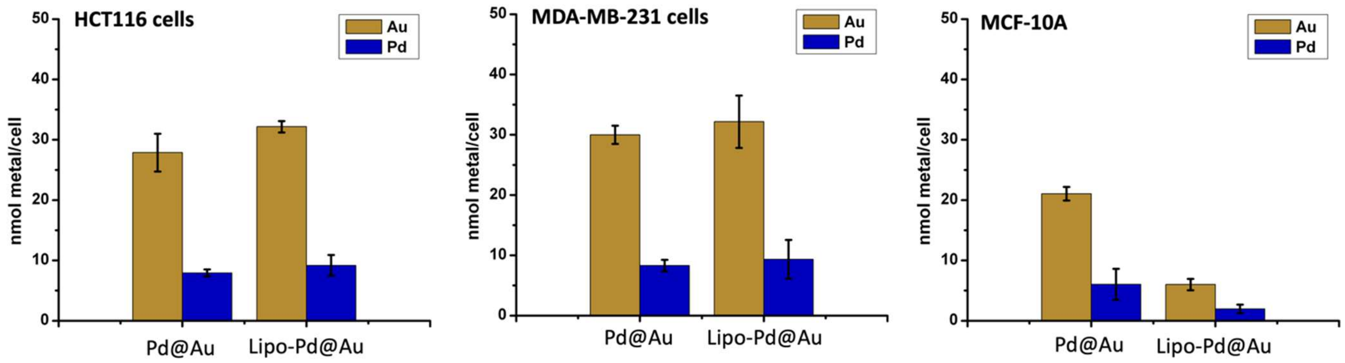

Figure S15. Cell uptake of Pd@Au and lipo-Pd@Au. Quantification of Au and Pd content inside the cell (nmol of metal/cell). Analysis performed by ICP-MS. Error bars: ±SD, n = 3.

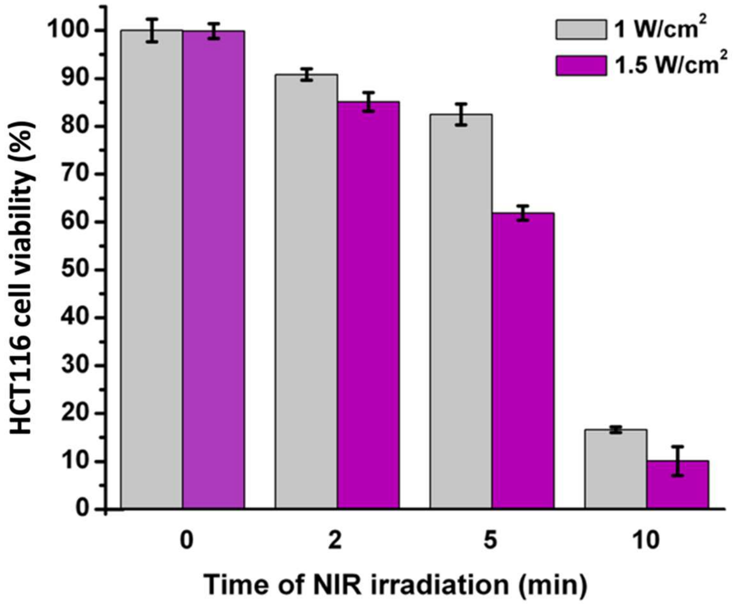

Figure S16. Photothermal effect in HCT116 cells pre-treated with **lipo-Pd@Au** (80 µg/mL) after NIR irradiation at two laser power densities (1.0 and 1.5 W/cm^2) for 2, 5 and 10 min. PrestoBlue viability assay was performed 24 h after NIR irradiation. Error bars: ±SEM, n=3.

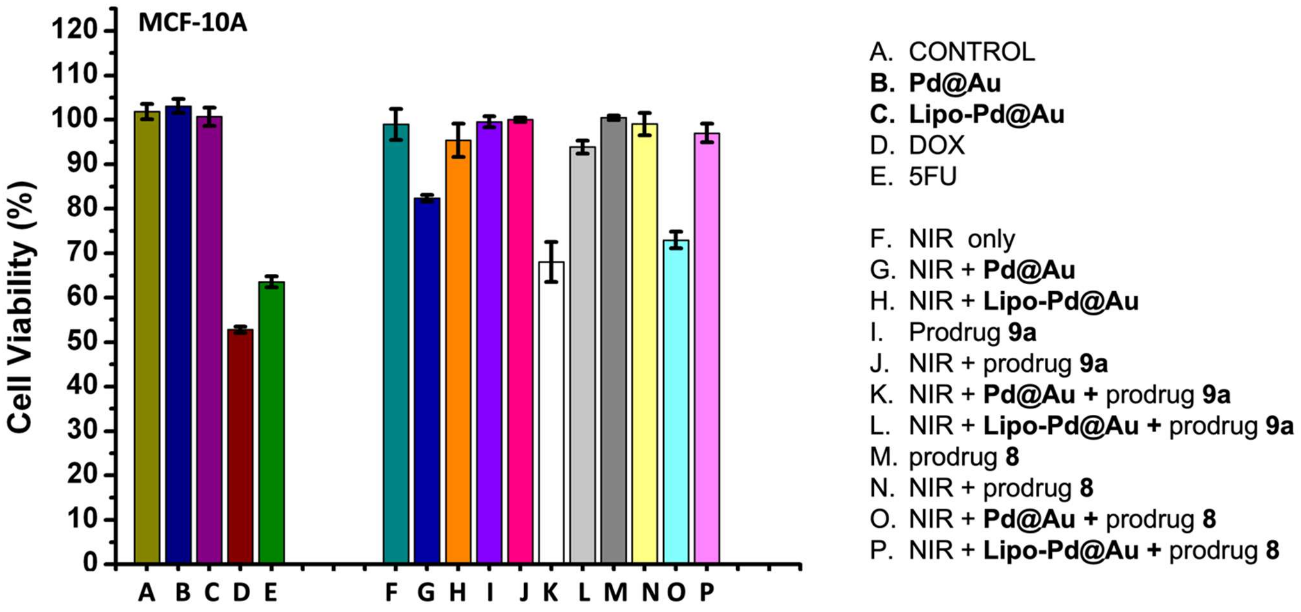

Figure S17. Combined photothermal chemotherapy by plasmonic effects and **lipo-Pd@Au**-mediated conversion of prodrug **8** into **5FU** or prodrug **9a** into **DOX** after NIR irradiation in MCF-10A cells under different treatment conditions. [Prodrug **8** /5FU]= 100 µM and [Prodrug **9a** /DOX]= 10 µM. Cells were pre-treated with **Pd@Au** or **Lipo-Pd@Au** for 6 h before prodrug addition, followed by 5 min of NIR irradiation. PrestoBlue viability assay was performed 24 h after NIR irradiation. Error bars: ±SEM, n=3.

Figure S18. Solubility data of prodrugs 9a-c.

9a	9b	9c
1mg/0.2mL PBS = insoluble at r.t	1mg/0.2mL PBS = partially soluble at 40ºC with stirring	1mg/0.2mL PBS = insoluble at r.t
1mg/0.5mL PBS = insoluble at 40ºC with vigorous stirring	1mg/0.4mL PBS = partially soluble at 40ºC with stirring	1mg/0.5mL PBS = insoluble at 40ºC with vigorous stirring
1mg/1mL PBS = insoluble at 40ºC, even after vigorous stirring	1mg/0.6mL PBS = mostly soluble at 40ºC with stirring	1mg/1mL PBS = insoluble at 40ºC, even after vigorous stirring

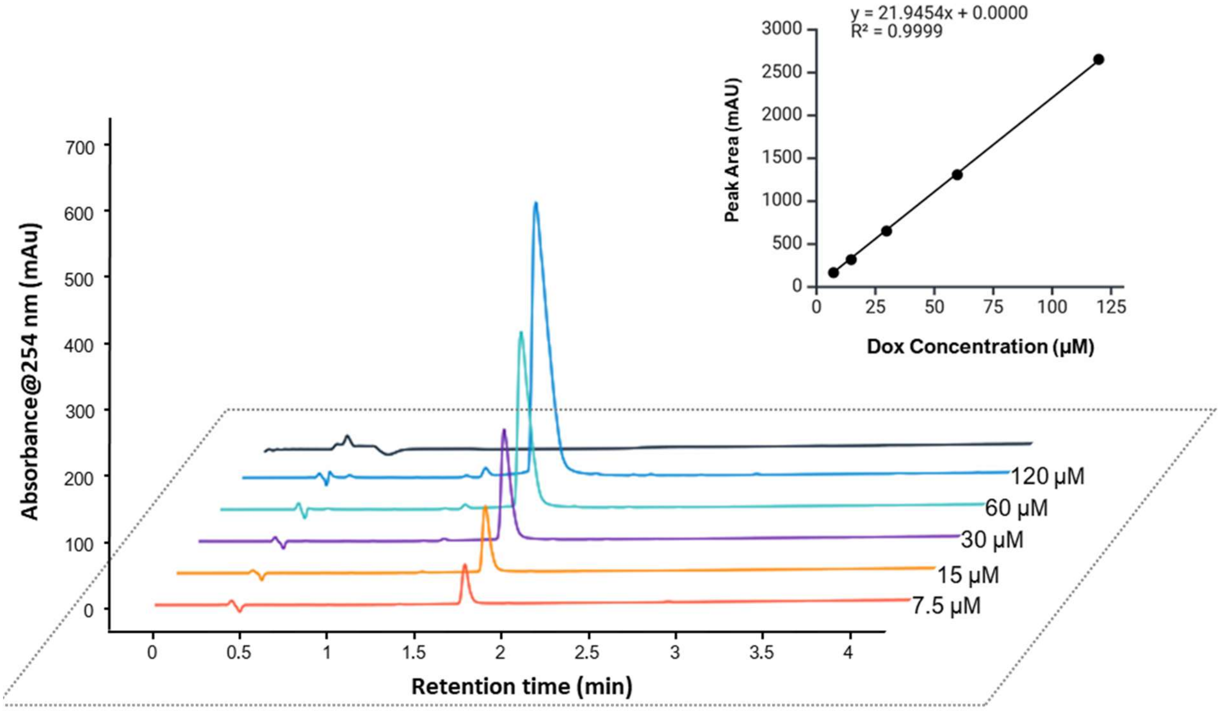

Figure S19. Doxorubicin calibration curve (inset) and HPLC traces of doxorubicin solutions used for the calibration curve. Stock solutions of Doxorubicin (7.5-120 µM in 10% PBS, 1% DMSO) were analysed by LC-MS. The area of the doxorubicin peak (at 1.8 min) was used to calculate the concentration of Doxorubicin released into solution by each prodrug.

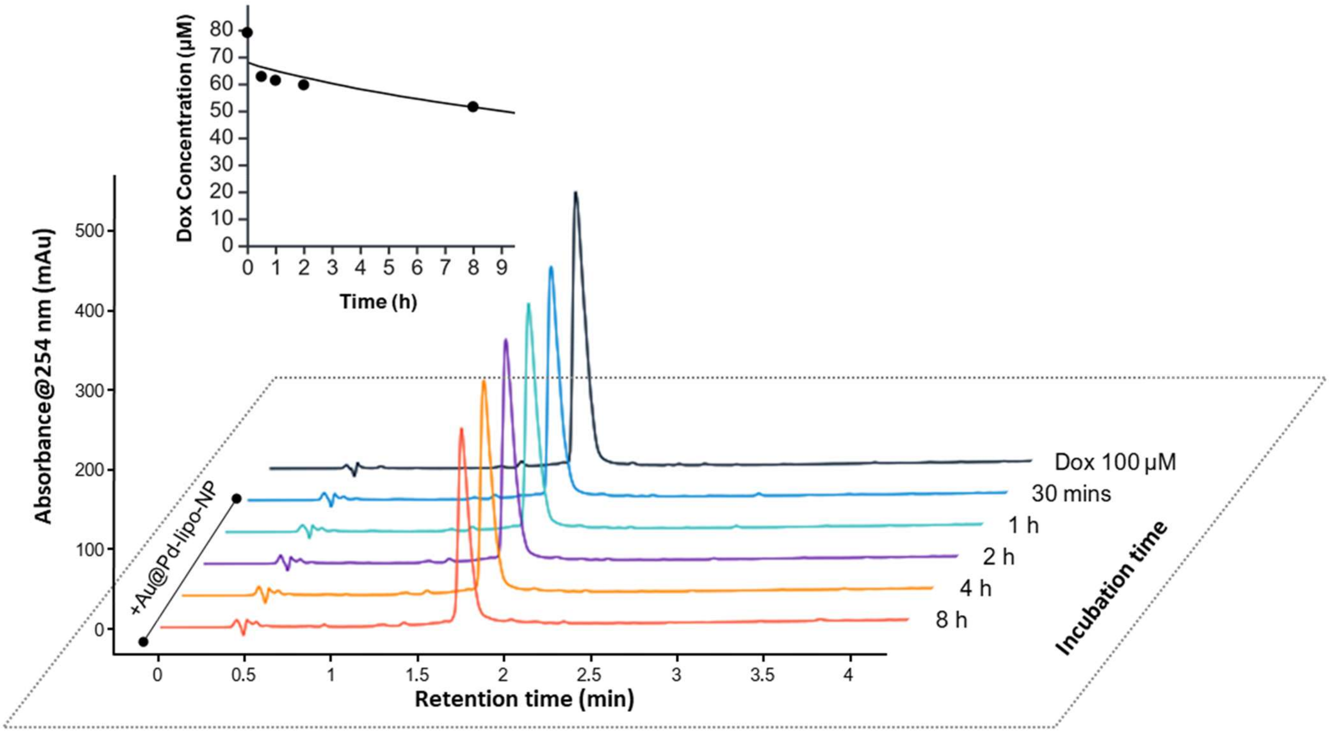

Figure S20. Doxorubicin + Pd@Au-lipo NPs control kinetics experiment. HPLC traces of reaction solutions. The area of the doxorubicin peak (at 1.8 min) was used to calculate the Doxorubicin concentration (see inset) from calibration curve **Figure S17**. Compound retention times: Doxorubicin: 1.8 min (found m/z = 544.5, calc'd = 544.15 $[M+H]^+$, 1088.1, cal'd = 1087.4 $[2M+H]^+$).

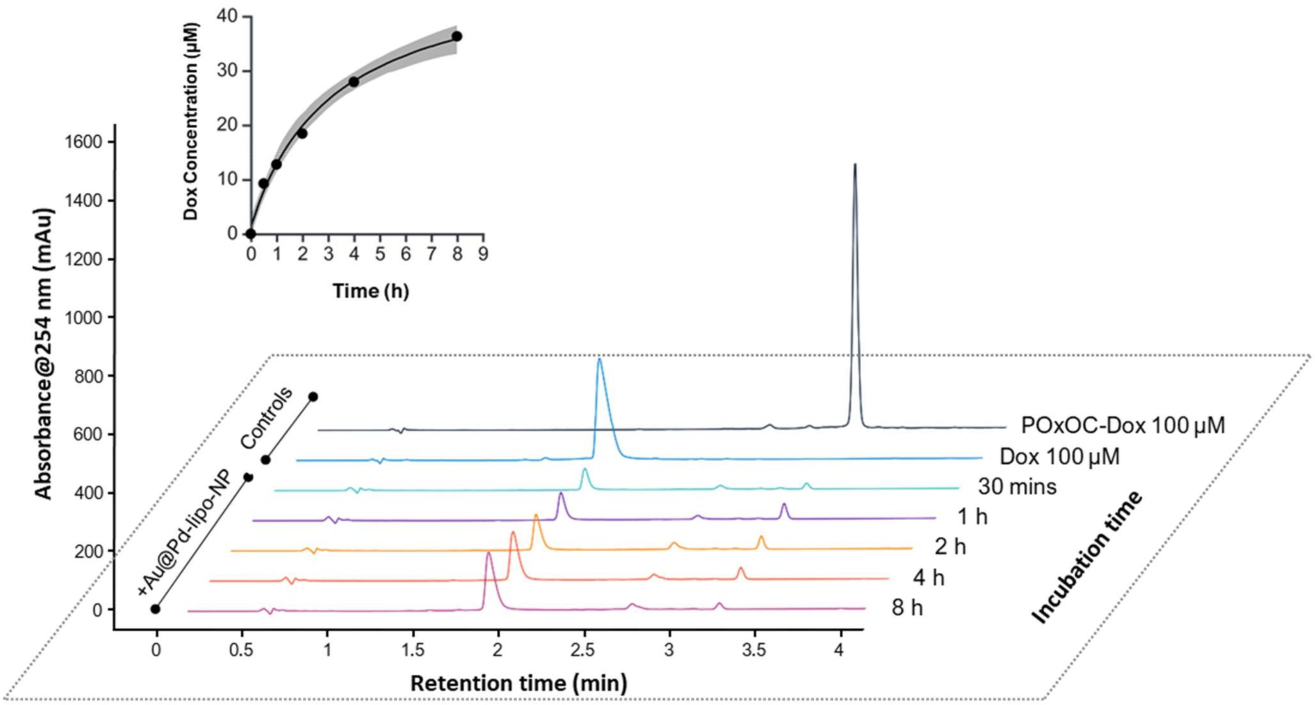

Figure S21. 9b + Pd@Au-lipo kinetics experiment. HPLC traces of reaction solutions. The area of the doxorubicin peak (at 1.8 min) was used to calculate the Doxorubicin concentration (see inset) from calibration curve **in Figure S17**. Compound retention times: Doxorubicin: 1.8 min (m/z = 544.5, calc'd = 544.15 $[M+H]^+$); de-propargylated intermediate: 2.6 min (m/z = 707.6; calc'd: 707.17 $[M+Na]^+$), **9b**: 3.1 min (m/z =745.7, calc'd = 745.19 $[M+Na]^+$).

Figure S22: Fitted exponential decay values for kinetic experiments
First order exponential decay: $y = y_0 + A_1*\exp(-(x\text{-}x0)/t_1)$, $k = 1/t_1$

	9a		9b		9c	
y_0	11.0	±0.4	38.6	±3.6	7.13	0.33
x_0	0.0445	±8×10^6	-0.0463	±8×10^6	0.500	0.00
A_1	-10.9	±1×10^7	-37.5	±1×10^8	-7.17	0.33
**t_1**	**6.70**	**±0.59**	**3.06**	**±0.76**	**29.8**	**3.94**
apparent *k*	0.15		0.33		0.03	
Reduced Chi-Sqr	0.08		4.52		0.06	
R-Square (COD)	1.00		0.99		0.99	
Adj. R-Square	0.99		0.97		0.99	

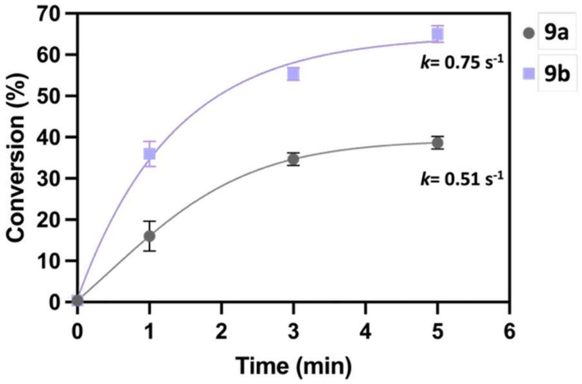

Figure S23. Study of the conversion kinetics for **9a** and **9b** prodrugs under NIR irradiation.

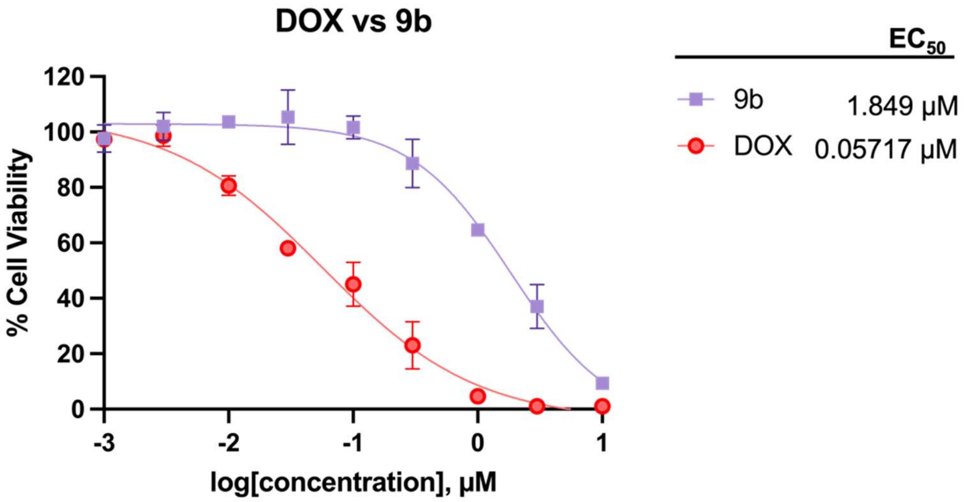

Figure S24. Dose-response curves of **DOX and 9a-c** in MDA-MB-231 cells (5 days treatment).

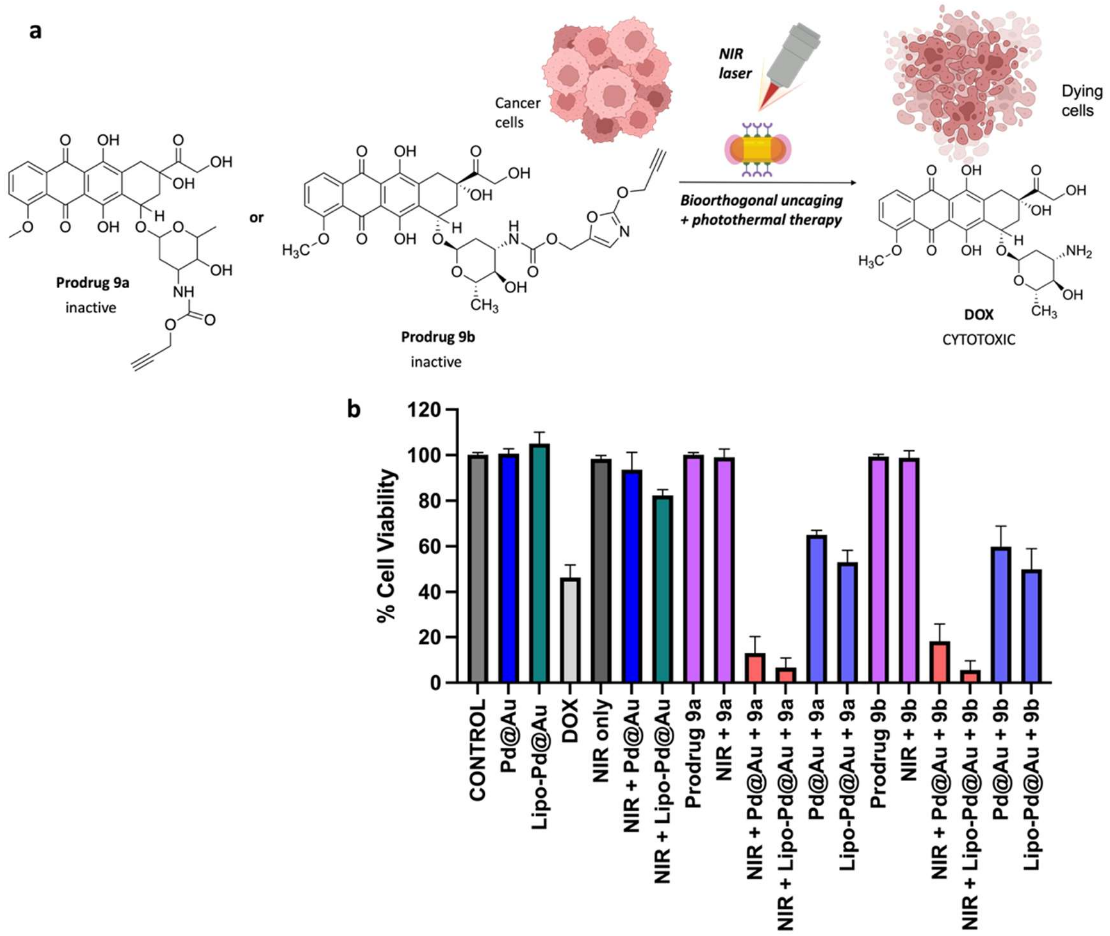

Figure S25. a, Combined photothermal chemotherapy by plasmonic effects and lipo-Pd@Au-mediated conversion of prodrug **9a** or **9b** into **DOX** after NIR irradiation. **b**, Cell viability assay in MDA-MB-231 breast cancer cells under different treatment conditions. Cells were pre-treated with **Lipo-Pd@Au** for 6 h before prodrug addition, followed by either 5 min of NIR irradiation or no irradiation. PrestoBlue viability assay was performed 24 h after NIR irradiation. Error bars: ±SEM, n=3.
